# Design, Synthesis, and In Vivo Evaluation of C1-Linked 4,5-Epoxymorphinan Haptens for Heroin Vaccines

**DOI:** 10.3390/molecules27051553

**Published:** 2022-02-25

**Authors:** Agnieszka Sulima, Fuying Li, Jeffrey Brian Morgan, Phong Truong, Joshua F. G. Antoline, Therese Oertel, Rodell C. Barrientos, Oscar B. Torres, Zoltan Beck, Gregory H. Imler, Jeffrey R. Deschamps, Gary R. Matyas, Arthur E. Jacobson, Kenner C. Rice

**Affiliations:** 1 Drug Design and Synthesis Section, Molecular Targets and Medications Discovery Branch, Intramural Research Program, National Institute on Drug Abuse and the National Institute on Alcohol Abuse and Alcoholism, National Institutes of Health, Department of Health and Human Services, 9800 Medical Center Drive, Bethesda, MD 20892, USA; agnieszka.sulima@nih.gov (A.S.); fuyingli1982@163.com (F.L.); brian.morgan.fpc@gmail.com (J.B.M.); ptruong30044@yahoo.com (P.T.); antoline.joshua@epa.gov (J.F.G.A.); 2 Laboratory of Adjuvant and Antigen Research, US Military HIV Research Program, Walter Reed Army Institute of Research, 503 Robert Grant Avenue, Silver Spring, MD 20910, USA; toertel968@gmail.com (T.O.); rodellcbarrientos@gmail.com (R.C.B.); oscarbuenatorres@gmail.com (O.B.T.); zoltan.beck@gmail.com (Z.B.); gmatyas@hivresearch.org (G.R.M.); 3 U.S. Military HIV Research Program, Henry M. Jackson Foundation for the Advancement of Military Medicine, 6720A Rockledge Drive, Bethesda, MD 20817, USA; 4 Center for Biomolecular Science and Engineering, Naval Research Laboratory, Washington, DC 20375, USA; gregory.imler@nrl.navy.mil (G.H.I.); jeff.deschamps@verizon.net (J.R.D.)

**Keywords:** 4,5-epoxymorphinan, hapten, heroin, vaccine, opioid use disorder

## Abstract

In our continuing effort to develop effective anti-heroin vaccines as potential medications for the treatment of opioid use disorder, herein we present the design and synthesis of the haptens: 1-AmidoMorHap (**1**), 1-AmidoMorHap epimer (**2**), 1 Amido-DihydroMorHap (**3**), and 1 Amido-DihydroMorHap epimer (**4**). This is the first report of hydrolytically stable haptenic surrogates of heroin with the attachment site at the C1 position in the 4,5-epoxymorophinan nucleus. We prepared respective tetanus toxoid (TT)–hapten conjugates as heroin vaccine immunogens and evaluated their efficacy in vivo. We showed that all TT–hapten conjugates induced high antibody endpoint titers against the targets but only haptens **2** and **3** can induce protective effects against heroin in vivo. The epimeric analogues of these haptens, **1** and **4**, failed to protect mice from the effects of heroin. We also showed that the in vivo efficacy is consistent with the results of the in vitro drug sequestration assay. Attachment of the linker at the C1 position induced antibodies with weak binding to the target drugs. Only TT-**2** and TT-**3** yielded antibodies that bound heroin and 6-acetyl morphine. None of the TT–hapten conjugates induced antibodies that cross-reacted with morphine, methadone, naloxone, or naltrexone, and only TT-**3** interacted weakly with buprenorphine, and that subtle structural difference, especially at the C6 position, can vastly alter the specificity of the induced antibodies. This study is an important contribution in the field of vaccine development against small-molecule targets, providing proof that the chirality at C6 in these epoxymorphinans is a vital key to their effectiveness.

## 1. Introduction

Approximately 130,000 Americans died from drug overdose due to heroin from 1999 to 2019, an average of more than four deaths for every 100,000 people [1]. The combined economic cost of opioid use disorder (OUD) and fatal overdose in the United States totaled ~$1.02 trillion, based on 2017 estimates [2]. Injection drug use also increases the risk of spreading HIV, hepatitis C virus, and other infectious diseases due to needle sharing among drug users. Recent reports suggest that the COVID-19 pandemic has accelerated drug overdose deaths, accounting for 81,000 deaths within the 12 months in the period ending May 2020 [3]; these overdose deaths appear to be increasing and current reports indicate that drug overdose deaths have reached the 100,000 level in the United States in the past year [3]. Clearly, the opioid epidemic is a severe public health burden, and we need to find novel and practical approaches to address this crisis.

**Figure 1:**
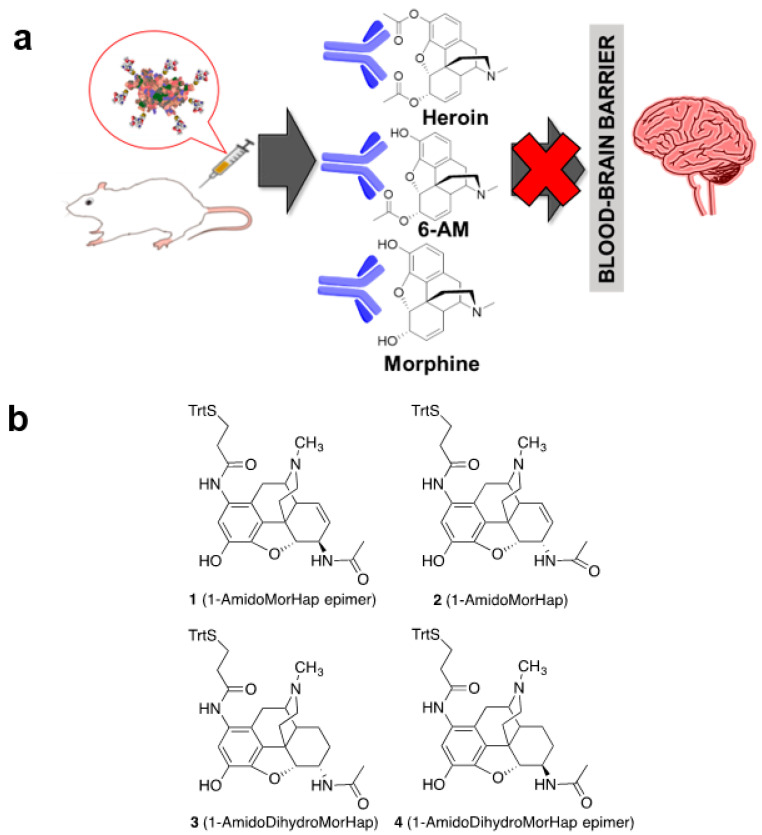
Proposed mechanism of vaccine interaction with heroin and its metabolites (**a**). Structure of haptens **1**–**4** (**b**).

Immunotherapy continues to emerge as a potential approach to address the incidence of fatal relapse for OUD [4,5,6,7]. Immunotherapy involves either the use of active or passive immunization through a vaccine or monoclonal antibodies (mAbs), respectively. The antibodies bind and sequester the drug in the blood, effectively preventing the drug to cross the blood–brain barrier, blocking its physiological effects (Figure 1a). There are three main components of vaccines against small-molecule drugs: (1) a molecular surrogate of the target drug called a hapten, (2) an immunogenic protein, and (3) an adjuvant. In this final formulation, the active immunogen is the protein–hapten conjugate. The design and efficacy of vaccines to drugs of abuse—for heroin [8,9,10], fentanyl [11,12], oxycodone [13], methamphetamine [14], morphine [15], and others [5]—have been reported and extensively studied in animals. It is known that hapten design impacts the specificity of the vaccine-induced antibodies [16,17,18,19]. Specificity is extremely vital for OUD therapy because not only the ability of the antibodies to bind the target drug effectively is considered but also the cross-reactivity to therapeutic drugs [5]. Vaccine-induced antibodies should not cross-react with existing drugs used to treat OUD [5]. To this end, it is extremely important to determine the optimum hapten design that would induce antibodies with the desired specificity.

In our continuing effort to develop effective anti-heroin vaccines [10,16,18,19,20,21,22,23,24,25], we have now designed a series of haptens (**1**–**4**, Figure 1b) based on the attachment of the linker to the C1 position in the 4,5-epoxymorophinan nucleus. Haptens have previously been examined with a linker attached to the nitrogen atom, as well as at the C3, C6, and C14 positions of the 4,5-epoxymorphinan nucleus [17,18,19]. Our studies of those compounds led to the proposal of a facial recognition hypothesis [16]. We have also reported that a hapten for an effective heroin vaccine was highly dependent on the specific substituent at the C6 position [19]; former work indicated that a C6-amido substituent was the most useful substituent of the several that we designed for a heroin vaccine [19]. We have now asked the question of whether the linker could be placed elsewhere on the 4,5-epoxymorphinan nucleus, at, for example, the C1 position. The facial recognition hypothesis would predict that the C1 was a feasible position for the attachment of the linker.

For a C1-linked molecule, we conceived of four possible molecular arrangements for the introduction of a C6-amido group. It could be introduced as a C6α or C6β substituent, with or without a double bond in the ring (i.e., a hapten based on morphine or dihydromorphine). We synthesized all four of these compounds, 1-AmidoMorHap epimer (*N*-((7*S*,7a*R*)-7-acetamido-9-hydroxy-3-methyl-2,3,4,4a,7,7a-hexahydro-1*H*-4,12-methanobenzofuro [3,2-*e*]isoquinolin-11-yl)-3-(tritylthio)propanamide, hapten (**1**), 1-AmidoMorHap (*N*-((7*R*,7a*R*)-7-acetamido-9-hydroxy-3-methyl-2,3,4,4a,7,7a-hexahydro-1*H*-4,12-methanobenzofuro[3,2-*e*]isoquinolin-11-yl)-3-(tritylthio)propanamide, hapten (**2**), 1 Amido-DihydroMorHap (*N*-((7*S*,7a*R*)-7-acetamido-9-hydroxy-3-methyl-2,3,4,4a,5,6,7,7a-octahydro-1*H*-4,12-methanobenzofuro[3,2-*e*]isoquinolin-11-yl)-3-(tritylthio)propanamide, hapten (**3**), and 1-AmidoDihydroMorHap epimer (*N*-((7*R*,7a*R*)-7-acetamido-9-hydroxy-3-methyl-2,3,4,4a,5,6,7,7a-octahydro-1*H*-4,12-methanobenzofuro[3,2-*e*]isoquinolin-11-yl)-3-(tritylthio)propanamide, hapten (**4**) (Figure 1).

## 2. Chemistry

### 2.1. Synthesis of Hapten ***1***

**Scheme 1:**
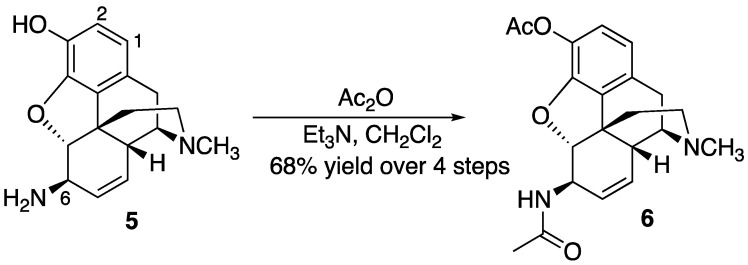
Synthesis of acetamido compound intermediate **6** from 6β-amino compound **5** [19].

Initially, the synthesis of *N*-((7*R*,7a*R*)-7-acetamido-9-hydroxy-3-methyl-2,3,4,4a,7,7a-hexahydro-1*H*-4,12-methanobenzofuro[3,2-*e*]isoquinolin-11-yl)-3-(tritylthio)propanamide (1-AmidoMorHap epimer, **1**, Figure 1) began with morphine as the starting material and followed the previously used route [19]. Briefly, the 6-amino compound **5** (Scheme 1) was prepared through 3-monoacetylation of morphine base using a modified Welsh procedure, followed by a Mitsunobu phthalimidation and removal of the phthalimide protection [19]. Simultaneous acetylation of the 3-hydroxyl and 6-amino groups gave the intermediate **6** at an overall yield of 68% over the four steps (Scheme 1). 

**Scheme 2:**
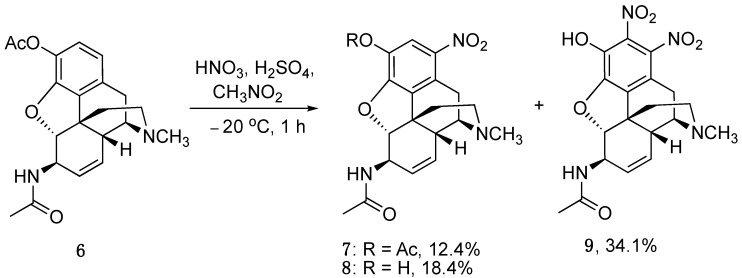
Over-nitration of intermediate **6**.

With **6** in hand, we sought to introduce an amino group at the C1 position via nitration on an activated C1, followed by reduction of the nitro group. Many nitration conditions were attempted. Neither nitronium tetrafluoroborate in DMSO at room temperature nor sodium nitrate in acetic acid gave any nitration, while sodium nitrite in TFA decomposed the substrate **6**. A classical HNO_3_/H_2_SO_4_ in nitromethane at −20 °C gave a mixture of the desired product **7**, phenol **8**, and over-nitrated product **9**, likely the result of nitration of phenol **8** after loss of the hydrolytically unstable acetate (Scheme 2). 

**Scheme 3:**
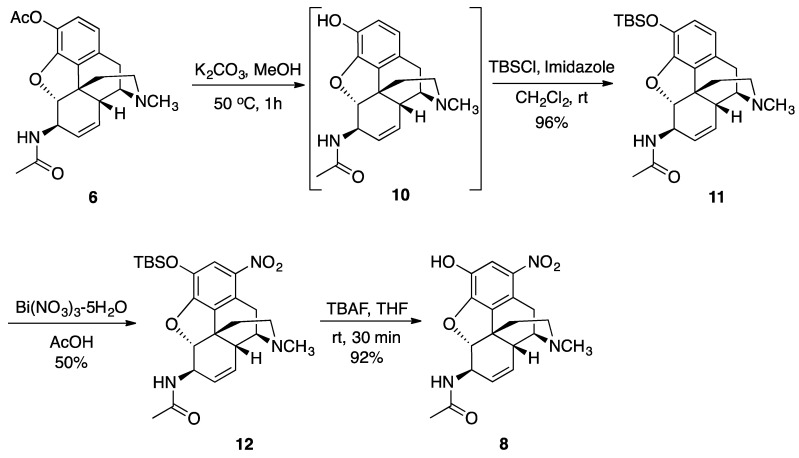
Synthesis of the 1-nitro intermediate **8**.

In an effort to avoid over-nitration, we opted to alter our C3 protection strategy (Scheme 3). Reasoning that the C2 position could be rendered sterically inaccessible using a bulky C3 protecting group, acetate **6** was hydrolyzed to free phenol **10** through basic methanolysis and re-protected as a TBS ether (**11**). Nitration with bismuth nitrate gave the desired mono-nitrated compound **12** in moderate yield. After nitration, the TBS group was removed with TBAF to give phenol **8** at a yield of 92%, a 44% overall yield from phenol **10**.

**Scheme 4:**
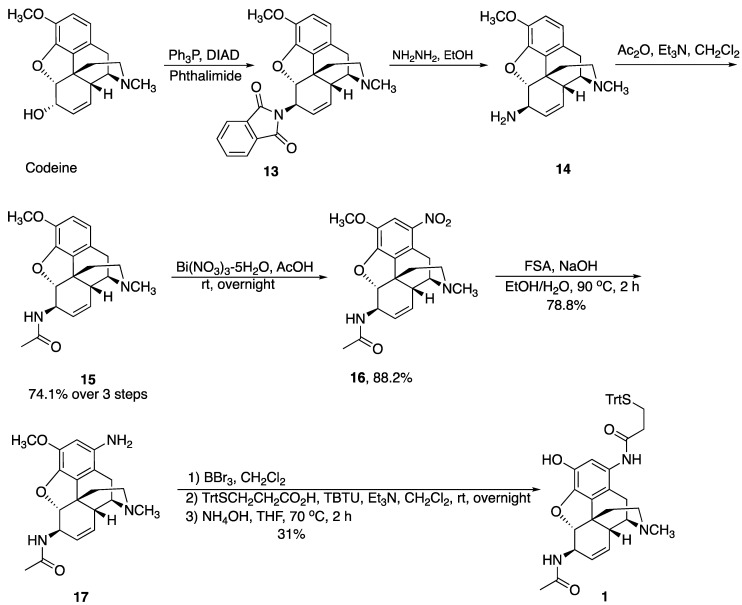
Synthesis of hapten **1** (1-AmidoMorHap epimer).

While effective, we thought that a more stable protective group at the C3 position would aid the nitration step, and that codeine, rather than morphine, would be a better starting material. We found that nitration with methyl ether gave a much better yield than that of acetate and TBS ether-protected substrate. Compound **15** was synthesized at a yield of 74% over 3 steps following the procedure used to prepare **6** (Scheme 4) [23]. 

**Figure 2:**
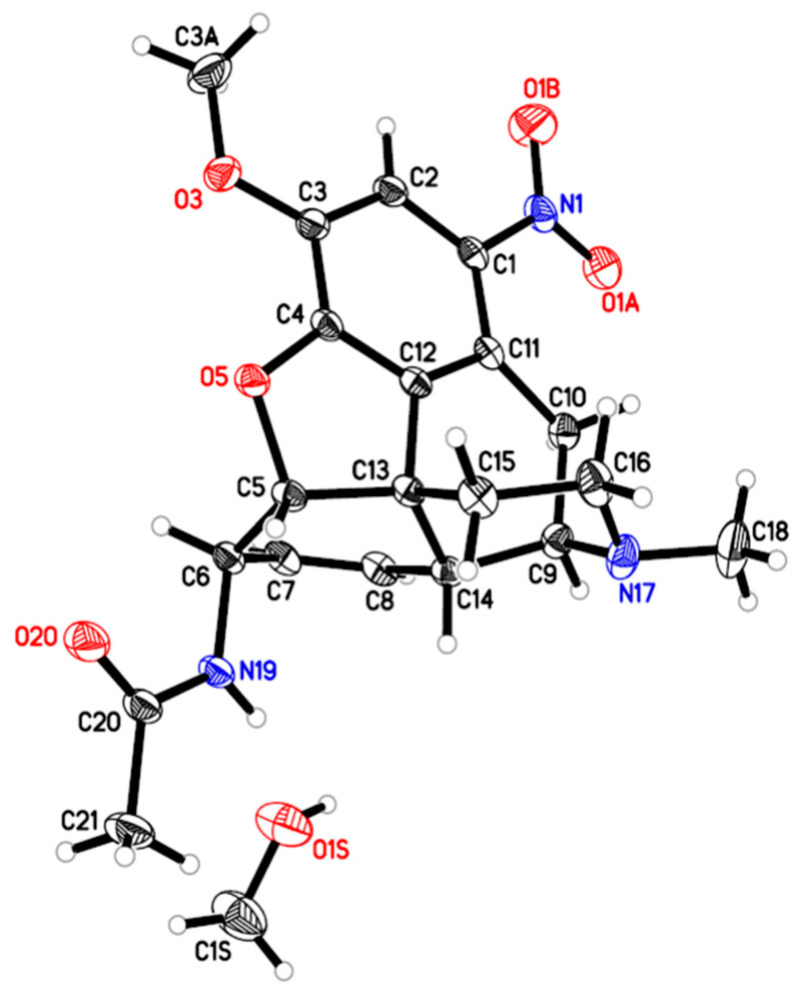
X-ray crystallographic structure determination of the absolute configuration of **16**. Thermal ellipsoids in the plot are at the 50% probability level.

With compound **15** as the substrate, the nitration using the nitric acid and sulfuric acid mixture in nitromethane at −20 to −10 °C still gave an over-nitrated product, while nitration with bismuth nitrate yielded the desired nitro compound **16** at a yield of 88% without over-nitration. The absolute configuration of **16** was confirmed by X-ray crystallographic analysis (Figure 2). The 1-nitro compound **16** was reduced with formamidinesulfinic acid (FSA) in basic solution to afford aniline **17**. The methyl ether was cleaved with BBr_3_ and the resulting hydrobromide salt was used directly without further purification due to its instability in solution. The resulting aminophenol was coupled with 3-(tritylthio)propanoic acid using *O*-(benzotriazol-1-yl)-*N*,*N*,*N*′,*N*′-tetramethyluronium tetrafluoroborate (TBTU) as the coupling reagent. Finally, the ester resulting from coupling with excess 3-(tritylthio)propanoic acid was hydrolyzed with NH_4_OH to give the desired hapten **1** at a yield of 31% (Scheme 4).

### 2.2. Synthesis of Hapten ***2***

**Scheme 5:**
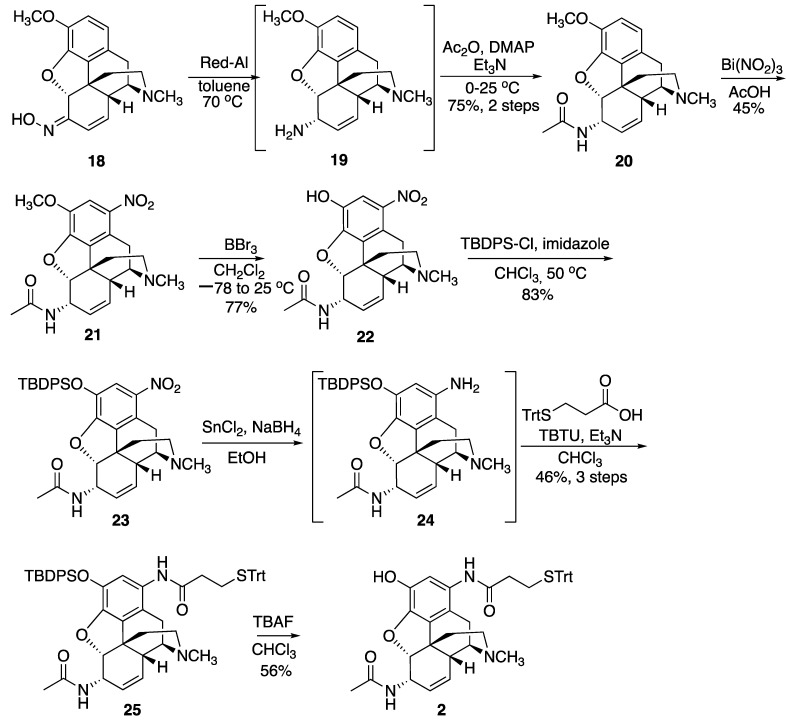
Synthesis of hapten **2** (1-AmidoMorHap).

The synthesis of hapten **2** (*N*-((7*S*,7a*R*)-7-acetamido-9-hydroxy-3-methyl-2,3,4,4a,7,7a-hexahydro-1*H*-4,12-methanobenzofuro[3,2-*e*]isoquinolin-11-yl)-3-(tritylthio)propanamide (1-AmidoMorHap, Figure 1) was accomplished in 8 steps from codeinone oxime (**18**, Scheme 5) [26]. Reduction of **18** with a five-fold excess of Red-Al in toluene at 70 °C afforded primary amine **19**, which was acylated crude to give amide **20** at a yield of 75% over 2 steps. Using a smaller excess of Red-Al significantly diminished the yield of the final acylated product. 

Following an analogous procedure to the synthesis of hapten **1**, nitration of **20** with bismuth nitrate hydrate gave **21** in modest yield (Scheme 5). Utilizing a similar reduction/deprotection sequence that was used in the synthesis of **1** afforded a highly unstable amino alcohol that degraded upon standing and during purification. Attempts to take the crude alcohol through the acylation/hydrolysis sequence afforded the desired hapten **2**, but the sequence was inconsistent, and the final hapten was extensively contaminated. Hypothesizing that the issue was oxidative instability and difficulty purifying the highly polar amino phenol, we sought to avoid having a free amino phenol present during the course of the synthesis, which necessitated replacing the methyl ether with a protecting group more amenable to late-stage removal. Cleavage of the C3-methyl (BBr_3_, −78–25 °C) followed by protection of the phenol as the *tert*-butyldiphenyl silyl ether gave **23** in good yield. Unfortunately, the silyl ether was labile under the strongly basic conditions of the FSA reduction. Switching to a tin chloride-mediated sodium borohydride reduction gave **24** followed by a TBTU coupling of the crude amine with the propionic acid linker affording amide **25** in moderate yield over two steps. Removal of the silyl group with tetrabutylammonium fluoride gave hapten **2** at a yield of 56%, a 6% overall yield from **18**. 

### 2.3. Synthesis of Hapten ***3***

**Scheme 6:**
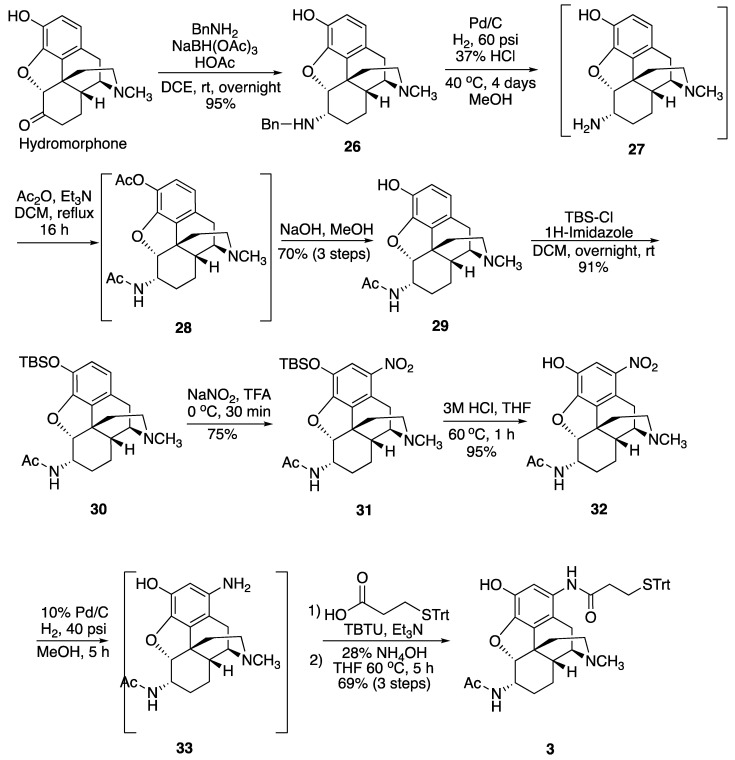
Synthesis of hapten **3** (1-AmidoDihydroMorHap).

The dihydro analog of hapten **2** (*N*-((7*S*,7a*R*)-7-acetamido-9-hydroxy-3-methyl-2,3,4,4a,5,6,7,7a-octahydro-1*H*-4,12-methanobenzofuro[3,2-*e*]isoquinolin-11-yl)-3-(tritylthio)propanamide, 1-AmidoDihydroMorHap, Figure 1) was synthesized from hydromorphone (Scheme 6). Reductive amination of hydromorphone with benzylamine gave secondary amine **26** in good yield. Hydrogenolysis of the benzyl group gave an intermediate (**27**) which underwent acylation to intermediate **28**. Basic hydrolysis of the aryl acetate **28** gave phenol **29** in 70% over three steps. The phenol was protected as the TBS ether (**30**) and nitrated under acidic conditions (NaNO_2_/TFA) to give the C1-nitro compound **31** (Scheme 6). 

Removal of the phenolic protecting group gave **32**, and the nitro moiety was reduced to the unstable amino compound **33** which was carried through a coupling/hydrolysis protocol to give hapten **3** in approximately 30% overall yield from hydromorphone.

### 2.4. Synthesis of Hapten ***4***

**Scheme 7:**
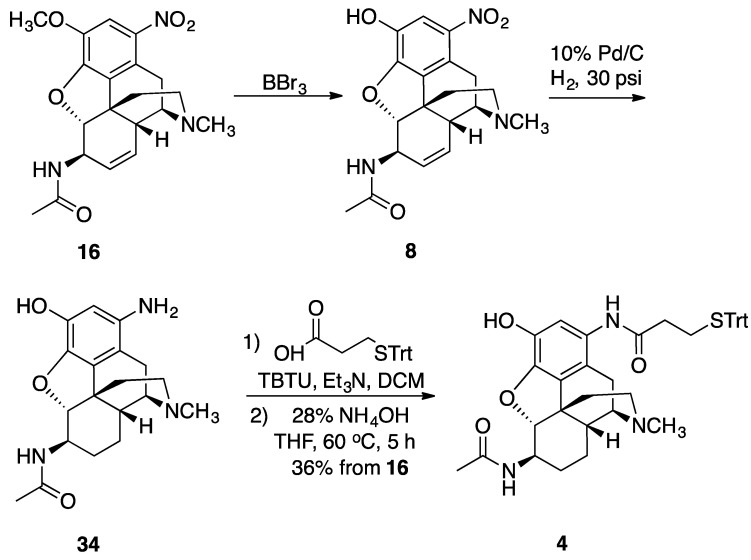
Synthesis of hapten **4** (1-AmidoDihydroMorHap epimer).

The epimer of 1-AmidoDihydroMorHap, *N*-((7*R*,7a*R*)-7-acetamido-9-hydroxy-3-methyl-2,3,4,4a,5,6,7,7a-octahydro-1*H*-4,12-methanobenzofuro[3,2-*e*]isoquinolin-11-yl)-3-(tritylthio)propanamide, Figure 1), was prepared in 7 steps from codeine at an overall yield of 19% (Scheme 7). Compound **16** (Scheme 4) served as the intermediate in the synthesis. Its absolute configuration was confirmed by X-ray crystallography (Figure 2). 

Compound **16** was subjected to BBr_3_ demethylation of the C3-methoxy moiety to give **8**, followed by formation of **34** by reduction of both the C-ring olefin and the C1-nitro to an amine using Pd/C. Coupling of the linker 3-(tritythio)propanoic acid and TBTU to the amine gave the desired compound hapten **4** at a yield of 36% over 3 steps from **16**.

## 3. Biology

### 3.1. Evaluation of TT–Hapten Conjugates In Vivo

**Figure 3:**
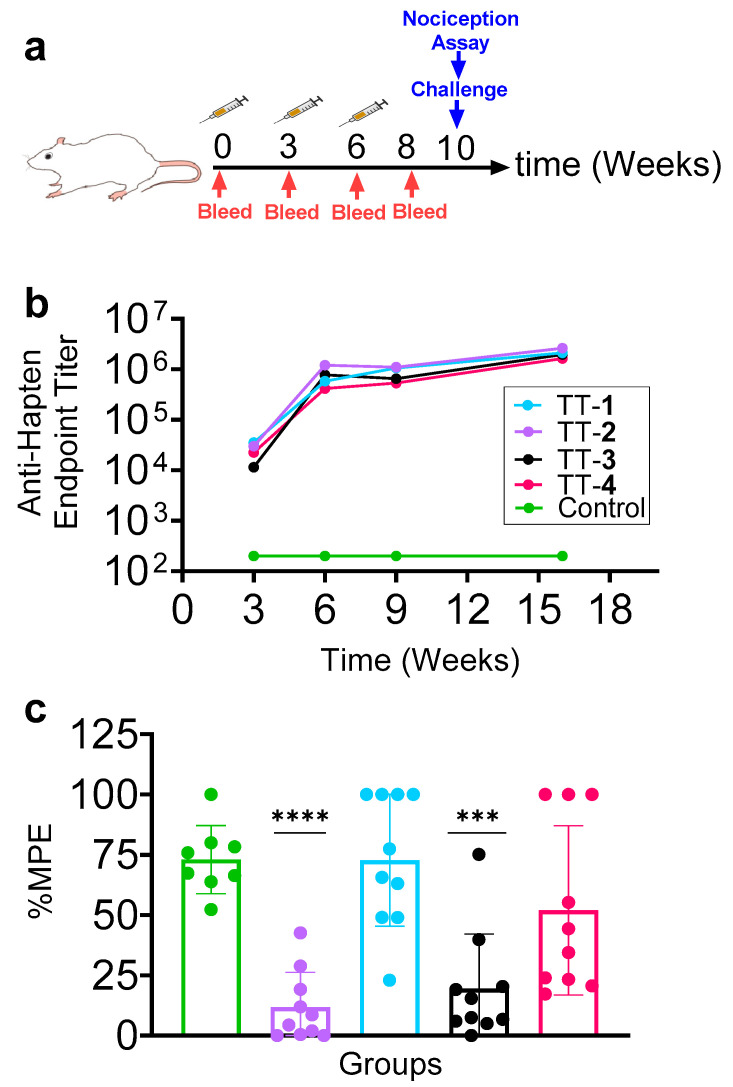
In vivo evaluation of the TT–hapten conjugates. (**a**) Experimental animal design. Female Balb/c mice (*n* = 10 per treatment group) were immunized with 50 µL of the TT–hapten formulation (10 µg TT–hapten containing ALF 50 mM MLV-PHAD and 30 µg Alhydrogel) at weeks 0, 3, and 6 and bled at weeks 0, 3, 6, and 8. (**b**) Anti-hapten ELISA of sera using BSA–hapten conjugates as coating agents. (**c**) Hot plate nociception assay. The color legends in (**c**) are the same as in (**b**). On week 10, mice received 1.0 mg/kg heroin (s.c.) and nociception was measured using the hot plate assay set at 54 °C. Response was reported in terms of %MPE. Results shown are the mean ± sem. One-way analysis of variance (ANOVA) with Dunnett’s correction for multiple comparisons was used to determine statistical significance: ****, *p* < 0.0001, ***, *p* = 0.0001, vs. control group.

The haptens were separately conjugated to tetanus toxoid (TT) carrier protein using the previously optimized method [18,23,24,27]. Vaccines were adsorbed to aluminum hydroxide and mixed with Army Liposome Formulation (ALF43) adsorbed to aluminum hydroxide as an adjuvant. Immunogenicity was assessed by immunizing mice and collecting sera at weeks 0, 3, and 6. Antibodies to the immunizing haptens were measured using ELISA that used BSA–hapten conjugates as coating agents. Results showed that all of the haptens induced high antibody endpoint titers (>10^5^ after the second vaccine dose) against their respective antigens (Figure 3b). This suggests that all of the vaccine candidates tested are immunogenic.

Multiple reports have suggested that antibody endpoint titers may not predict efficacy of a vaccine against small molecules such as drugs of abuse [5,23]. To this end, we assessed the efficacy of each vaccine candidate by in vivo hot plate assay. This assay measures the time it takes for the mouse to respond to a pain stimulus (heat). This has been used in the past to evaluate vaccines to drugs of abuse [27,28]. Our results showed that among the conjugates tested, only TT-**2** and TT-**3** gave low % MPE values, suggesting that mice were protected from the antinociceptive effects of heroin (Figure 3c). The other conjugates, TT-**1** and TT-**4,** carrying epimeric haptens showed no significant difference with the unvaccinated controls (Figure 3c). While all TT–hapten conjugates induced high antibody endpoint titers to the targets, only TT-**2** and TT-**3** showed protection against heroin, suggesting that antibody titers may not predict the efficacy of heroin vaccines in vivo. This is consistent with previous reports [23,29]. 

### 3.2. Determination of Drug Sequestration In Vitro

Anti-heroin vaccines are thought to act by inducing antibodies that sequester the drugs in the periphery [4,5,6]. The sequestered drug is prevented from crossing the blood–brain barrier due to increase in apparent size thus effectively blocking the drug’s physiological effects. In vivo, heroin quickly hydrolyzes to 6-acetyl morphine (6-AM) and morphine. Studies suggested that the physiological effects of heroin are mainly due to heroin and 6-AM with little contribution from other downstream metabolites such as morphine and the glycosylated metabolites [5,30]. 

**Figure 4:**
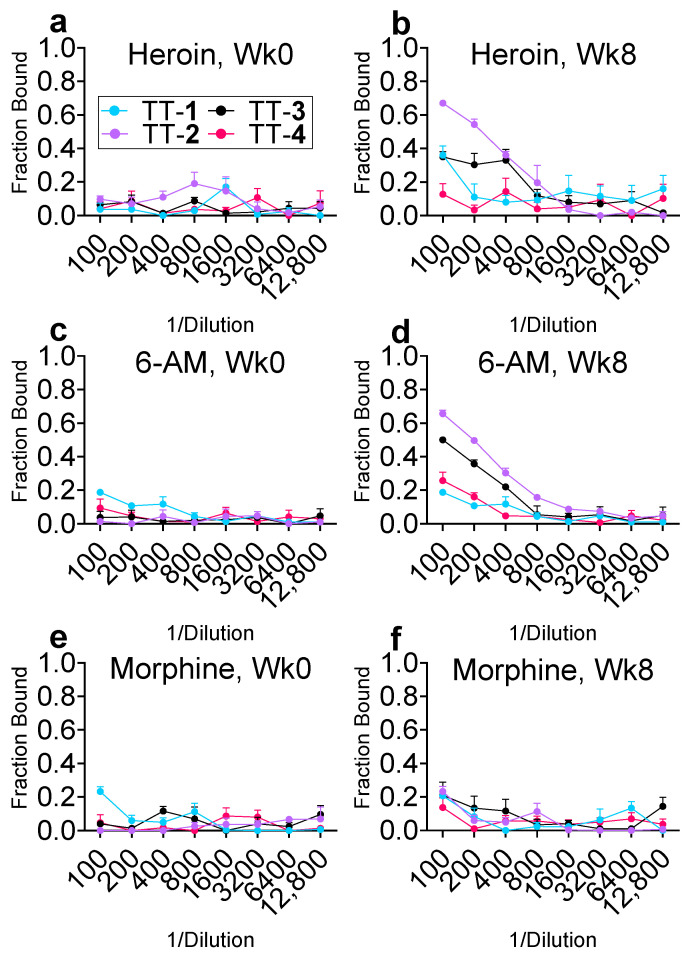
In vitro drug sequestration analysis of mice sera to heroin and metabolites. Mice sera (weeks 0 and 8) were diluted with ED buffer 5 nM of the indicated drug. ED buffer contained 3–5 mg/mL NaF to inhibit esterases that catalyze heroin degradation. Diluted mice sera were incubated in an ED plate against ED buffer. The concentration of the drug in both serum and buffer chambers were quantified by LC–MS/MS. Fraction bound was calculated and plotted to give the graphs above. Heroin (**a**,**b**); 6-AM (**c**,**d**); morphine (**e**,**f**). Results shown are the mean ± sem.

In vitro drug binding experiments using equilibrium dialysis and liquid chromatography tandem mass spectrometry (ED–LC–MS/MS) were performed to assess the drug sequestering potential of mice sera [22]. First, heroin and its two bioactive metabolites, 6-AM, and morphine were tested. Results showed that mice sera at week 8 after the first dose only TT-**2** provided the highest binding to heroin (Figure 4a,b). TT-**1** and TT-**3** showed slightly (fraction bound ~0.4) but significantly higher than before the first dose (week 0). We arbitrarily defined a fraction bound value of <0.5 at low serum dilutions (1:200 or less) as weak binding. A similar trend was observed for the metabolite 6-AM where TT-**2** and TT-**3** showed the highest binding (fraction bound >0.4) (Figure 4c,d). All conjugates tested did not bind morphine (Figure 4e,f). These results are consistent with the in vivo efficacy data (Figure 3c), where only TT-**2** and TT-**3** induced protective effects against heroin.

**Figure 5:**
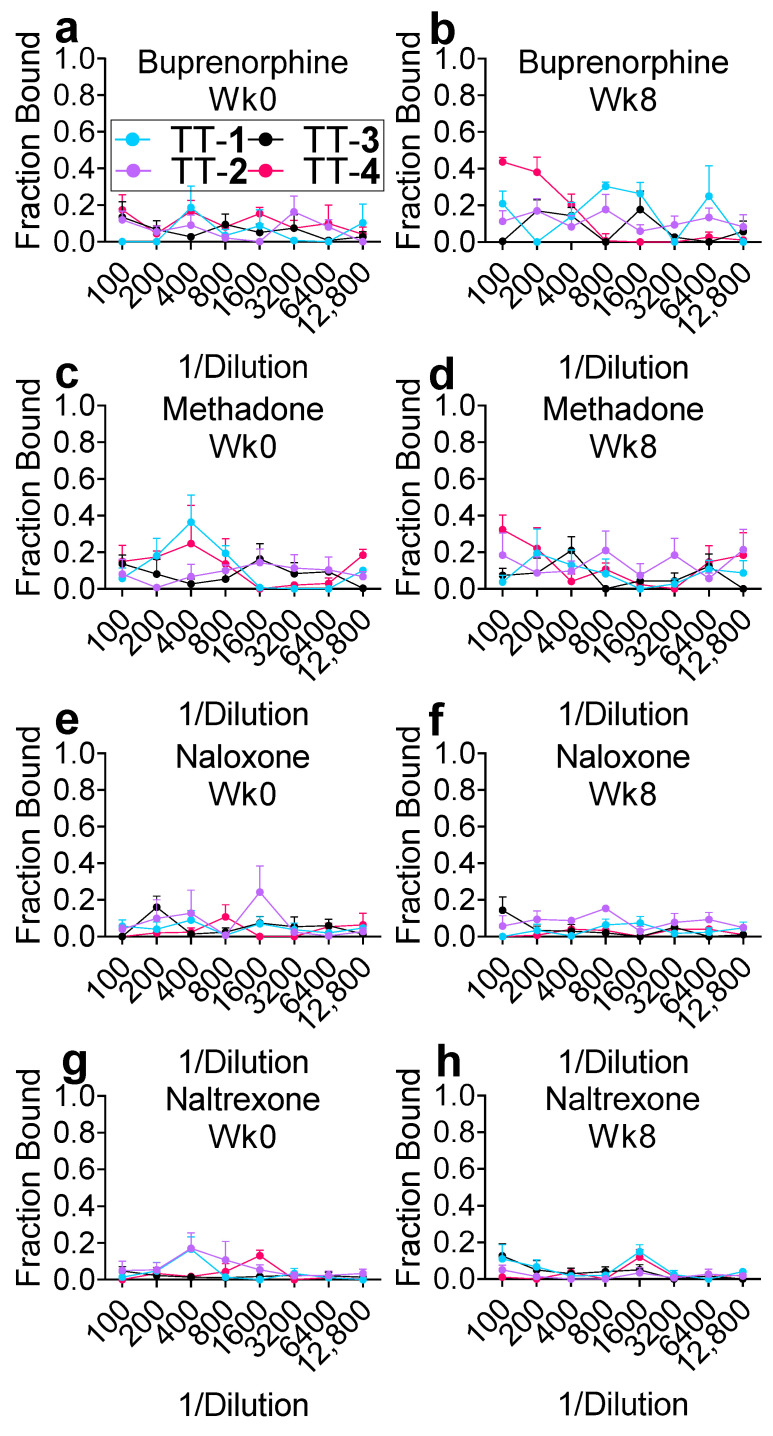
In vitro drug sequestration analysis of mice sera to therapeutics. Mice sera (weeks 0 and 8) were diluted with ED buffer 5 nM of the indicated drug. ED buffer contained 3–5 mg/mL NaF to inhibit esterases that catalyze heroin degradation. Diluted mice sera were incubated in an ED plate against ED buffer. The concentration of the drug in both serum and buffer chambers were quantified by LC–MS/MS. Fraction bound was calculated and plotted to give the graphs above. Buprenorphine (**a**,**b**); methadone (**c**,**d**); naloxone (**e**,**f**); naltrexone (**g**,**h**). Results shown are the mean ± sem.

We also tested the in vitro sequestration of drugs used to treat OUD, namely, buprenorphine, methadone, naloxone, and naltrexone. Results indicated that TT-**1**, TT-**2**, and TT-**4** did not induce antibodies that can bind any of these drugs (Figure 5). Only TT-3 induced antibodies that weakly bound buprenorphine (Figure 5b). 

**Figure 6:**
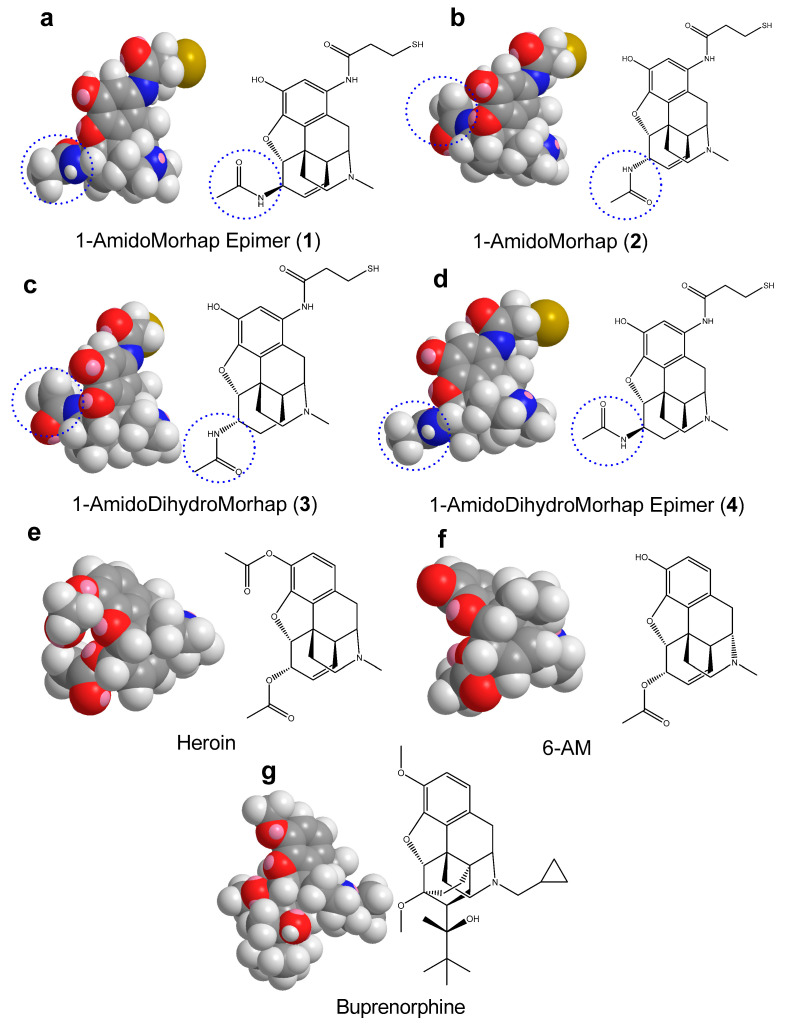
3D models of the haptens and target drugs. Structures were constructed and geometry optimized using the built-in MM2 method in ChemDraw 18.1. (**a**) 1-AmidoMorhap Epimer (**1**); (**b**) 1-AmidoMorhap (**2**); (**c**) 1-AmidoDihydroMorhap (**3**); (**d**) 1-AmidoDihydroMorhap (**4**); (**e**) heroin; (**f**) 6-AM; (**g**) buprenorphine. Structures were presented in a similar orientation to facilitate comparison.

The facial recognition hypothesis [16,17] predicts that TT-**2** and TT-**3** would induce protection in vivo because of their resemblance to the target dug, heroin. Indeed, we observed that mice sera induced by these conjugates bound heroin and 6-AM (Figure 4a–d). Interestingly, the epimers of haptens **1** and **4** failed to show binding to heroin or 6-AM. Heroin and 6-AM have an absolute configuration opposite that of the epimeric haptens **1** and **4** (Figure 4a–d). This suggested that the configuration of the acetamide in the hapten at the C6 position is essential to induce antibodies that would bind heroin and 6-AM. This observation reinforced the facial recognition hypothesis that even subtle difference in the hapten structure, in this case, chirality at the C6 position, can vastly alter the specificity of the induced antibodies. The chirality of haptens and its effect on efficacy have been previously demonstrated for methamphetamine vaccines [5]. This was further visualized by constructing the Molecular Mechanics 2 (MM2)-optimized 3D models of the haptens and the target drugs (Figure 6). It was observed that heroin and 6-AM resembled more closely the structures of haptens **2** and **3**, than haptens **1** and **4**, with emphasis on the substituent at the C6 position. The carbon–carbon double bonds in the morphinan ring did not significantly alter the 3D orientation of the haptens. The antibodies induced by TT-**2** and TT-**3** were notably weak (Figure 4). This may be because neither of these haptens exactly mimicked the three-dimensional orientation of the target drugs. Importantly, none of the conjugates induced antibodies that can bind morphine, methadone, naloxone, or naltrexone. This observation was expected because the structure of the haptens is distinct from the structure of these drugs [17]. Interestingly, we observed that TT-3 induced antibodies that weakly recognized buprenorphine (fraction bound ~0.4 at 1:100 serum dilution). When modeled, the structure of buprenorphine has some resemblance to hapten **3** (Figure 1), where the methoxy group in the C6 position of buprenorphine is projected away from the linker attachment site, and thus a part of the “face” recognized by the immune system. This structural feature is unique to buprenorphine among the therapeutics tested (buprenorphine, naloxone, naltrexone, and methadone). This difference may be attributed to the absence of the hydroxyl group at the C9 position that is present in naloxone and naltrexone. This hydroxyl group induced geometric change in those two molecules that cannot occur in buprenorphine, leaving the latter with a unique “face” that slightly mimicked that of hapten **3**. Naloxone and naltrexone share the same epoxymorphinan ring structure as the haptens and buprenorphine, but their structures in three-dimensional space suggest no significant similarity to the “face” of the haptens exposed to the immune system for recognition. Collectively, these results suggested that linker attachment at the C1 position is feasible without altering the cross-reactivity of the antibodies against OUD therapeutics. It is important that anti-opioid vaccines induce antibodies that will not cross-react with therapeutic drugs since the ultimate goal of a vaccine is to complement existing treatments [4,5,6]. 

The present study has limitations. First, this study only evaluated a vaccine based on the core morphinan structure with the linker attachment site at the C1 position. The effect of chirality at C6 in the epoxymorphinan ring in the hapten on the quality of the vaccine-induced antibodies should also be investigated where the linker attachment is on another site in the epoxymorphinan nucleus that projects a distinctly different face of the hapten. Second, this study did not attempt to evaluate the functional changes in the vaccine-induced antibodies. Recently, we have demonstrated that affinity maturation takes place. Conducting this same study for the TT–hapten conjugates described here in a future study might provide valuable insights as to how hapten-specific antibody affinity changes with time. Third, since only an in-bred mouse strain was used in this study, it would be interesting to see how these vaccines behave in other animal models, such as rats, rhesus macaques, and rabbits. 

## 4. Experimental

### 4.1. Materials and Methods

All reagents were obtained from commercial sources and used without further purification. Melting points were determined on a Mettler Toledo MP70 Melting Point System (Mettler Toledo, Columbus, OH, USA) and are uncorrected. Proton nuclear magnetic resonance (^1^H NMR, 400 MHz) and carbon nuclear magnetic resonance (^13^C NMR, 100 MHz) spectra were recorded on a Varian 400 wide-bore spectrometer (Varian, Palo Alto, CA, USA) in CDCl_3_ (unless otherwise noted) with the values given in ppm and *J* (Hz) assignments of ^1^H resonance coupling. For ^1^H NMR spectra (CDCl_3_), the residual solvent peak was used as the reference (7.26 ppm) while the central solvent peak was used as the NMR reference (77.0 ppm in CDCl_3_). The high-resolution mass spectra were obtained on a Waters (Waters Corp., Milford, MA USA) LCT Premier Time-of-Flight (TOF) mass spectrometer using electrospray ionization (ESI). Thin-layer chromatography (TLC) was performed on 0.25 mm Analtech GHLF silica gel and was used to determine the completion of the reaction depending on the polarity of the compounds, using CHCl_3_: MeOH: 28% NH_4_OH, 90:9:1. Gas chromatography (GC) was performed on an Agilent Technologies 6850 Series system (Agilent Technologies, Santa Clara, CA, USA) equipped with Agilent Technologies 7683B series injector and Agilent Technologies 5975C VL MSD Triple-Axis detector. Flash column chromatography was performed using Teledyne Isco prepacked silica gel (RediSep) columns (Teledyne Isco, Lincoln, NE, USA). Elemental analyses were performed by Micro-Analysis, Inc, Wilmington, DE, USA, and were within ±0.4% for C, H, and N. ^1^H and ^13^C NMRs of novel compounds and X-ray crystallographic data for compound **16** can be found in the Supplementary Material.

The materials and reagents used for the biological evaluation of the haptens are the following: the NHS-(PEG)_2_-maleimide crosslinker [(SM-(PEG)_2_], spin desalting columns (Zeba^TM^, 7K MWCO), dialysis cassettes (Slide-A-Lyzer^TM^, 10K MWCO), Pierce^TM^ bicinchoninic acid (BCA) protein assay kit, and the bovine serum albumin (BSA) that was used for coupling reactions were purchased from Fisher Scientific (Rockford, IL, USA). Tetanus toxoid (TT) was purchased from Statens Serum Institut (Copenhagen, Denmark). Dulbecco’s phosphate-buffered saline (DPBS, pH 7.4) was purchased from Quality Biological Inc. (Gaithersburg, MD, USA,). Lipids used to prepare liposomal adjuvant, 1,2-dimyristoyl-*sn*-glycero-3-phosphoglycerol (DMPG), 1,2-dimyristoyl-*sn*-glycero-3-phosphocholine (DMPC), synthetic monophosphoryl lipid A (PHAD^®^) (MPLA), and cholesterol were purchased from Avanti Polar Lipids (Alabaster, AL, USA). Alhydrogel^®^ was purchased from Brenntag (Reading, PA, USA). Mouse anti-tetanus toxoid monoclonal antibody was purchased from Abcam (Cambridge, MA, USA). Peroxidase-linked sheep anti-mouse IgG (γ-chain specific) was purchased from The Binding Site (San Diego, CA, USA). The 2,2′-Azino-di(3-ethylbenzthiazoline-6-sulfonate) (ABTS) peroxidase substrate system was purchased from KPL, Inc. (Gaithersburg, MD, USA). Mass spectrometry grade water and acetonitrile (ACN), methanol (MeOH), and rapid equilibrium dialysis (ED) plates (12 kDa MWCO) were purchased from Fisher Scientific (Rockford, IL, USA). Sodium fluoride was purchased from Sigma-Aldrich (Milwaukee, WI, USA). Mass spectrometry standards, heroin-*d_5_*, 6-acetylmorphine-*d_3_*, morphine-*d_3_ d*,*l*-methadone, and buprenorphine·HCl, were from Lipomed Inc. (Cambridge, MA, USA. Naloxone-*d_5_* and naltrexone-*d_3_* were from Cerilliant (Round Rock, TX, USA). 

*N-((4R,4aR,7R,7aR,12bS)-7-Acetamido-9-hydroxy-3-methyl-2,3,4,4a,7,7a-hexahydro-1H-4,12-methanobenzofuro[3,2-e]isoquinolin-11-yl)-3-(tritylthio)propanamide* (**1**). To a solution of 17 (0.728 g, 2.05 mmol) in CHCl_3_ (40 mL) at −78 °C was added BBr_3_ (2.56 g, 10.25 mmol) and the solution was stirred at room temperature overnight. The reaction was quenched with MeOH and the solvent was removed in vacuo. MeOH (20 mL) was added and the solvent was removed in vacuo. The procedure was repeated three times. The crude product was treated with CHCl_3_ (50 mL) and 3-(tritylthio)propanoic acid (1.07 g, 3.07 mmol), Et_3_N (1.04 g, 1.42 mL, 10.2 mmol), *O*-(benzotriazol-1-yl)-*N*,*N*,*N*′,*N*′-tetramethyluronium tetrafluoroborate (TBTU, 1.32 g, 4.1 mmol) were added. After stirring overnight, the solvent was removed in vacuo and the residue was dissolved in THF (20 mL) and 28% NH_4_OH (5 mL) was added. The mixture was heated to 50 °C overnight. The solvent was removed in vacuo, the residue was treated with CHCl_3_ (100 mL) and the solution was washed with saturated NaHCO_3_ and brine and dried over anhydrous Na_2_SO_4_. After filtration and removal of solvent in vacuo, the residue was purified by flash chromatography (CHCl_3_:MeOH:NH_4_OH, 90:9:1) to afford hapten **1** (0.43 g, 31.1% over 3 steps) as yellow solid. [α]_D_^20^ −75.0° (*c* 1.0, CHCl_3_:MeOH, 19:1); ^1^H NMR (400 MHz, CDCl_3_) *δ* 7.40 (d, *J* = 7.6 Hz, 6H), 7.25 (t, *J* = 8.0 Hz, 6H), 7.18 (t, *J* = 7.2 Hz, 3H), 6.72 (s, 1H), 5.66 (m, 1H), 5.49 (d, *J* = 10.4 Hz, 1H), 4.54 (s, 1H), 4.30 (d, *J* = 5.6 Hz, 1H), 3.24 (m, 1H), 2.86 (m, 2H), 2.54 (m, 3H), 2.31 (m, 4H), 2.16 (t, *J* = 7.2 Hz, 2H), 2.04 (dd, *J* = 18.4, 6.0 Hz, 1H), 1.93 (m, 4H), 1.74 (d, *J* = 11.6 Hz, 1H); ^13^C NMR (100 MHz, CDCl_3_+CD_3_OD) *δ* 170.8, 169.5, 144.7 (3), 142.8, 139.2, 131.9, 130.0, 129.6 (6), 128.6, 128.0 (6), 127.1, 126.8 (3); 119.4, 113.1, 93.1, 67.0, 58.9, 47.0, 44.1, 42.9, 39.2, 36.0, 35.3, 28.0, 22.94, 22.89, 18.0; MS (ESI): *m*/*z* 672.3 [M + H]^+^. HRMS (ESI) *m*/*z* calcd for C_41_H_42_N_3_O_4_S 672.2891, found 672.2891; Anal. calcd. for C_41_H_41_N_3_O_4_S•1.0 *t*-BuOH•0.5H_2_O: C, 71.59; H, 6.94; N, 5.57. Found: C, 71.50; H, 7.04; N, 5.78 (after 1 was lyophilized from *t*-BuOH).

*N-((4R,4aR,7S,7aR,12bS)-7-Acetamido-9-hydroxy-3-methyl-2,3,4,4a,7,7a-hexahydro-1H-4,12-methanobenzofuro[3,2-e]isoquinolin-11-yl)-3-(tritylthio)propanamide* (**2**). To a solution of silanol **25** (0.116 g, 0.127 mmol) in THF (2 mL) was added 1.0 M tetrabutylammonium fluoride solution (0.510 mL, 0.508 mmol, 4 equiv). The solution was stirred for 16 h and then concentrated in vacuo to give a brown oil. The oil was purified by silica gel chromatography (CHCl_3_:MeOH:28% NH_4_OH, 99:0.9:0.1 to 90:9:1) to give hapten **2** as a clear oil (0.048 g, 56%). The oil was lyophilized in *tert*-butanol to give hapten **2** as a white powder suitable for biological studies. [α]_D_^20^ −0.726° (*c* 0.65, MeOH); ^1^H NMR (400 MHz; CDCl_3_) *δ* 7.41 (d, *J* = 7.7 Hz, 5H), 7.36–7.17 (m, 10H), 7.09 (s, 1H), 6.71 (d, *J* = 8.6 Hz, 1H), 5.37 (d, *J* = 9.4 Hz, 1H), 5.23 (d, *J* = 9.5 Hz, 1H), 4.75 (d, *J* = 6.1 Hz, 1H), 4.48 (s, 1H), 3.33 (d, *J* = 2.0 Hz, 1H), 2.80 (d, *J* = 18.0 Hz, 1H), 2.66 (s, 1H), 2.57 (dd, *J* = 10.6, 6.0 Hz, 3H), 2.32 (s, 4H), 2.16 (t, *J* = 6.7 Hz, 2H), 2.08–2.02 (m, 2H), 1.96 (s, 3H), 1.75 (d, *J* = 12.8 Hz, 1H); ^13^C NMR (100 MHz; CDCl_3_) *δ* 170.3, 169.8, 144.5, 143.5, 138.7, 130.80, 130.66, 129.5, 128.0, 127.6, 126.8, 118.1, 111.7, 90.5, 67.1, 58.5, 47.2, 46.3, 43.6, 42.9, 36.3, 27.9, 23.1, 18.1; HRMS (ESI) (*m*/*z*) calcd for C_41_H_42_N_3_O_4_S, 672.2896; found 672.2897. Anal calcd for C_41_H_41_N_3_O_4_S•1.0 *t*-BuOH•1.45 H_2_O: C, 70.00; H, 7.04; N, 5.44; found C, 69.90; H, 6.86; N, 5.54. 

*N-((4R,4aR,7S,7aR,12bS)-7-acetamido-9-hydroxy-3-methyl-2,3,4,4a,5,6,7,7a-octahydro-1H-4,12-methanobenzofuro[3,2-e]isoquinolin-11-yl)-3-(tritylthio)propanamide* (**3**). To a solution of **32** (400 mg, 1.07 mmol) in MeOH (20 mL) was added 10% (w/w) Pd/C (200 mg). The reaction mixture was stirred under H_2_ atmosphere (40 psi) for 5 h at room temperature. The mixture was filtered through a Celite pad, and the solvent was concentrated in vacuo to give the crude intermediate amine **3** (*N*-((4*R*,4a*R*,7*S*,7a*R*,12b*S*)-11-amino-9-hydroxy-3-methyl-2,3,4,4a,5,6,7,7a-octahydro-1*H*-4,12-methanobenzofuro[3,2-*e*]isoquinolin-7-yl)acetamide) as a yellow solid that was used without purification. The intermediate **3** was dissolved in DCM (20 mL) and Et_3_N (4.2 mL). 3-(tritylthio)propanoic acid (1.12 g, 3.21 mmol, 3 equiv) and *O*-(benzotriazol-1-yl)-*N*,*N*,*N’*,*N’*-tetramethyluronium tetrafluoroborate (TBTU, 1.20 g, 3.74 mmol, 3.5 equiv) were added to the reaction mixture, and the mixture was stirred at room temperature for 16 h. The reaction was quenched with saturated NaHCO_3_ (20 mL) and extracted with DCM (3 × 25 mL). The combined organic layers were washed with brine, dried over MgSO_4_, filtered, and solvent removed in vacuo. The residual material was dissolved in THF (10 mL) and 28% NH_4_OH (6 mL) and heated at 60 °C for 5 h. After cooling to room temperature, H_2_O (15 mL) was added, and the mixture was extracted with CHCl_3_:MeOH (6:1) (3 × 30 mL). The combined organic layers were dried over MgSO4, filtered, concentrated in vacuo, and the product was purified through silica gel chromatography (CHCl_3_:MeOH:28% NH_4_OH, 90:9:1)) to give hapten **3** (495 mg, 69% yield) as a pale brown solid, mp 170–174 °C. [α]^20^_D_ −66.5° (*c* 1.0, CHCl_3_); HRMS (ESI) *m*/*z* 674.3049 [C_41_H_44_N_3_O_4_S (M+H) requires 674.3052; ^1^H NMR (400 MHz, CDCl_3_) *δ* 7.49–7.12 (m, 16H), 6.07 (d, *J* = 9.0 Hz, 1H), 4.58 (d, *J* = 4.3 Hz, 1H), 4.25–4.10 (m, 1H), 3.10 (d, *J* = 3.5 Hz, 1H), 2.73 (d, *J* = 18.1 Hz, 1H), 2.61 (t, *J* = 6.8 Hz, 2H), 2.50 (dd, *J* = 11.7, 3.8 Hz, 1H), 2.35–2.09 (m, 8H), 1.99–1.85 (m, 4H), 1.67–1.59 (m, 2H), 1.45–1.40 (m, 1H), 1.08–0.98 (m, 1H), 0.84–0.76 (m, 1H); ^13^C NMR (400 MHz, CDCl_3_) *δ* 169.8, 169.7, 144.4, 143.1, 138.3, 129.9, 129.5, 128.0, 127.5, 126.8, 117.8, 111.6, 89.9, 67.1, 59.2, 46.6, 46.0, 42.9, 42.9, 37.1, 36.4, 35.9, 27.9, 23.3, 22.0, 20.2, 17.5. Anal. calcd for C_41_H_43_N_3_O_4_S•1.25 H_2_O: C, 70.71; H, 6.59; N, 6.03, found C, 70.86; H, 6.55; N, 6.03.

*N-((4R,4aR,7R,7aR,12bS)-7-acetamido-9-hydroxy-3-methyl-2,3,4,4a,5,6,7,7a-octahydro-1H-4,12-methanobenzofuro[3,2-e]isoquinolin-11-yl)-3-(tritylthio)propanamide* (**4**). A mixture of the amine **34** (105 mg, 0.31 mmol), 3-(tritylthio)propanoic acid (160 mg, 0.46 mmol), Et_3_N (0.13 mL, 0.92 mmol) and TBTU (196 mg, 0.61 mmol) in CH_2_Cl_2_ (20 mL) was stirred at room temperature overnight, and then 50 °C for 3 h. The solution was diluted with CH_2_Cl_2_ (80 mL) and washed with saturated NaHCO_3_ and brine and dried over Na_2_SO_4_. After filtration and removal of solvent in vacuo, the residual material was purified by flash chromatography (CHCl_3_:MeOH:NH_4_OH, 90:9:1) to give hapten **4** (147 mg, 71.4%). [α]_D_^20^ −97.8° (*c* 0.99, CHCl_3_:MeOH 19:1); ^1^H NMR (400 MHz, CDCl_3_) *δ* 7.44 (d, *J* = 8.0 Hz, 6H), 7.28 (t, *J* = 8.0 Hz, 6H), 7.21 (t, *J* = 7.2 Hz, 3H), 6.15 (s, 1H), 5.68 (d, *J* = 7.2 Hz, 1H), 4.48 (d, *J* = 8.0 Hz, 1H), 3.39 (brs, 2H), 3.18 (m, 2H), 2.70 (d, *J* = 18.0 Hz, 1H), 2.51 (m, 5H), 2.38 (s, 3H), 2.16 (td, *J* = 12.0, 4.0 Hz, 1H), 2.07 (dd, *J* = 18.0, 5.6 Hz, 1H), 1.83 (m, 5H), 1.71 (dd, *J* = 12.4, 2.0 Hz, 1H), 1.61 (m, 1H), 1.50 (m, 1H), 0.80–1.01 (m, 1H); ^13^C NMR (100 MHz, CDCl_3_) *δ* 170.3, 169.6, 144.8, 139.6, 137.6, 133.7, 131.8, 129.7 (6), 128.1 (6), 126.9 (3), 118.0, 107.4, 92.9, 67.0, 59.1, 53.4, 47.2, 43.9, 43.0, 42.7, 35.1, 33.4, 28.3, 27.0, 24.6, 23.9, 17.3; MS (ESI): *m*/*z* = 674.3 [M + H]. HRMS (ESI) *m*/*z* calcd for C_41_H_44_N_3_O_4_S 674.3047; found, 674.3048; Anal. calcd for C_41_H_43_N_3_O_4_S•1.0 *t*-BuOH•0.55 H_2_O: C, 71.31; H, 7.19; N, 5.54. Found: C, 71.26; H, 7.06; N, 5.63, after lyophilization from *t*-BuOH.

*(4R,4aR,7R,7aR,12bS)-7-Acetamido-3-methyl-2,3,4,4a,7,7a-hexahydro-1H-4,12-methanobenzofuro[3,2-e]isoquinolin-9-yl acetate* (**6**). To a solution of the known phenolic amine **5 [23]** in CHCl_3_ (30 mL) at 0 °C was added a solution of Et_3_N (4.4 g, 6 mL, 43 mmol) and Ac_2_O (2.35 g, 2.2 mL, 23 mmol), and these were stirred at room temperature overnight, washed with a dilute solution of NaHCO_3_, H_2_O and brine, successively and dried over anhydrous Na_2_SO_4_. The crude product was purified by flash chromatography (CHCl_3_:MeOH:28% NH_4_OH, 90:9:1) to give the acetate **6** (2.42 g, 68% over 4 steps from morphine) as a white foam. ^1^H NMR (400 MHz, CDCl_3_) *δ* 6.71 (d, *J* = 8.0 Hz, 1H), 6.53 (d, *J* = 8.0 Hz, 1H), 5.78 (m, 2H), 5.56 (dd, *J* = 10.0, 2.0 Hz, 1H), 4.74 (s, 1H), 4.33 (t, *J* = 6.4 Hz, 1H), 3.29 (m, 1H), 3.01 (d, *J* = 18.8 Hz, 1H), 2.96 (s, 1H), 2.52 (dd, *J* = 12.4, 4.0 Hz, 1H), 2.38 (s, 3H), 2.29 (m, 2H), 2.20 (s, 3H), 1.97 (d, *J* = 12.4, 5.2 Hz, 1H), 1.92 (s, 3H), 1.78 (dd, *J* = 12.6, 2.0 Hz, 1H). ^13^C NMR (100 MHz, CDCl_3_) *δ* 169.9, 168.6, 148.9, 132.7, 132.4, 131.8, 131.4, 128.9, 121.9, 119.0, 93.2, 58.9, 49.6, 46.7, 44.1, 43.1, 40.2, 35.8, 23.2, 20.7, 20.6; MS (ESI) *m*/*z* = 369.2 [M + H]; HRMS (ESI) (*m*/*z*) calcd for C_21_H_25_N_2_O_4_ 369.1809; found 369.1810.

*(4R,4aR,7R,7aR,12bS)-7-Acetamido-3-methyl-11-nitro-2,3,4,4a,7,7a-hexahydro-1H-4,12-methanobenzofuro[3,2-e]isoquinolin-9-yl acetate* (**7**). To a solution of acetate **6** (1.09 g, 2.96 mmol) in CH_3_NO_2_ (60 mL) at −20 °C was added a mixture of HNO_3_ (0.75 g, 0.5 mL) and concentrated H_2_SO_4_ (1.3 mL) and the mixture was stirred at −20~−10 °C for 1 h. The mixture was diluted with H_2_O and basified with NaHCO_3_ powder. The two-layer mixture was extracted with CH_2_Cl_2_. The combined extracts were washed with H_2_O and brine and dried over anhydrous Na_2_SO_4_. After filtration and removal of solvent in vacuo, the residue was purified by flash chromatography (CHCl_3_:MeOH:NH_4_OH, 90:9:1) to afford three fractions containing **7** (152 mg, 12.4%), **8** (202 mg, 18.4%) and **9** (420 mg, 34.1%). Compound **7**: yellow foam; [α]_D_^20^ −132.3° (*c* 1.01, CHCl_3_). ^1^H NMR (400 MHz, CDCl_3_) *δ* 7.86 (s, 1H), 5.86 (m, 1H), 5.64 (m, 2H), 4.93 (s, 1H), 4.37 (t, *J* = 6.4 Hz, 1H), 3.54 (d, *J* = 20.0 Hz, 1H), 3.38 (m, 1H), 3.01 (s, 1H), 2.67 (dd, *J* = 20.4, 6.4 Hz, 1H), 2.58 (dd, *J* = 12.4, 4.0 Hz, 1H), 2.43 (s, 3H), 2.29 (s, 3H), 2.25 (td, *J* = 12.4, 2.0 Hz, 1H), 2.06 (td, *J* = 12.4, 5.2 Hz, 1H), 1.97 (s, 3H), 1.81 (dd, *J* = 12.6, 2.0 Hz, 1H). ^13^C NMR (100 MHz, CDCl_3_) *δ* 170.1, 167.9, 154.3, 139.4, 133.2, 133.1, 131.5, 130.9, 129.3, 121.5, 94.8, 58.0, 49.2, 46.2, 44.4, 43.2, 39.2, 35.9, 23.2, 21.3, 20.6; MS (ESI) *m*/*z* 414.2 [M + H]; HRMS (ESI) *m*/*z* calcd for C_21_H_24_N_3_O_6_ 414.1660; found 414.1658.

*N-((4R,4aR,7R,7aR,12bS)-9-Hydroxy-3-methyl-11-nitro-2,3,4,4a,7,7a-hexahydro-1H-4,12-methanobenzofuro[3,2-e]isoquinolin-7-yl)acetamide* (**8**): From **6** as a yellow foam; [α]_D_^20^ −125.4° (*c* 0.94, CHCl_3_:MeOH 9:1). ^1^H NMR (400 MHz, CDCl_3_+CD_3_OD) *δ* 7.57 (s, 1H), 5.71 (m, 1H), 5.56 (dd, *J* = 10.0, 1.6 Hz, 1H), 4.65 (s, 1H), 4.29 (d, *J* = 5.6 Hz, 1H), 3.42 (d, *J* = 20.0 Hz, 1H), 3.30 (m, 1H), 2.85 (s, 1H), 2.54 (td, *J* = 20.4, 6.0 Hz, 2H), 2.39 (s, 3H), 2.22 (m, 1H), 1.94 (m, 1H), 1.93 (s, 3H), 1.73 (dd, *J* = 12.6, 1.6 Hz, 1H). ^13^C NMR (100 MHz, CDCl_3_+CD_3_OD) *δ* 171.4, 150.4, 139.7, 139.6, 132.1, 131.0, 128.6, 123.5, 115.4, 94.3, 58.2, 49.9, 46.4, 43.9, 42.7, 38.2, 35.3, 22.4, 20.7; MS (ESI) *m*/*z* 372.2 [M + H]; HRMS (ESI) *m*/*z* calcd for C_19_H_22_N_3_O_5_ 372.1554; found 372.1554.

*Acetamide***8***from***12***:* To a solution of TBS ether **12** (0.8 g, 1.6 mmol) in THF (20 mL) was added a solution of TBAF (1 M in THF, 2 mL) and the solution was stirred at room temperature for 0.5 h. The solvent was evaporated, and the residue was purified by flash chromatography (CHCl_3_:MeOH:28% NH_4_OH, 80:18:2) to give **8** (0.55 g, 92.5%).

*N-((4R,4aR,7R,7aR,12bS)-9-Hydroxy-3-methyl-10,11-dinitro-2,3,4,4a,7,7a-hexahydro-1H-4,12-methanobenzofuro[3,2-e]isoquinolin-7-yl)acetamide* (**9**): From **6** as a yellow solid; MS (ESI) *m*/*z* 417.1 [M + H]; HRMS (ESI) *m*/*z* calcd for C_19_H_21_N_4_O_7_ 417.1405; found 417.1403. 

*N-((4R,4aR,7R,7aR,12bS)-9-((tert-Butyldimethylsilyl)oxy)-3-methyl-2,3,4,4a,7,7a-hexahydro-1H-4,12-methanobenzofuro[3,2-e]isoquinolin-7-yl)acetamide* (**11**). To a solution of acetate **6** (1.33 g, 3.6 mmol) in MeOH (20 mL) was added K_2_CO_3_ powder and the mixture was stirred for 1 h at 50 °C. The mixture was filtered and the filtrate was concentrated to give the crude intermediate **10** (*N*-((4*R*,4a*R*,7*R*,7a*R*,12b*S*)-9-hydroxy-3-methyl-2,3,4,4a,7,7a-hexahydro-1*H*-4,12-methanobenzofuro[3,2-*e*]isoquinolin-7-yl)acetamide). The crude phenol **10** was dissolved in CH_2_Cl_2_ (50 mL). Imidazole (294 mg, 4.3 mmol) was added, followed by TBSCl (650 mg, 4.3 mmol). The solution was stirred at room temperature for 2 h, washed with H_2_O and brine and dried over anhydrous Na_2_SO_4_. After filtration and removal of solvent in vacuo, the crude product was purified by purified by flash chromatography (CHCl_3_:MeOH:28% NH_4_OH, 95:4.5:0.5) to afford **11** (1.52 g, 96.2% over 2 steps) as a yellow foam. [α]_D_^20^ −170.0° (*c* 0.99, CHCl_3_); ^1^H NMR (400 MHz, CDCl_3_) *δ* 6.58 (d, *J* = 8.0 Hz, 1H), 6.44 (d, *J* = 8.4 Hz, 1H), 5.79 (m, 1H), 5.60 (dd, *J* = 10.0, 2.0 Hz, 1H), 5.33 (d, *J* = 6.8 Hz, 1H), 4.70 (s, 1H), 4.41 (t, *J* = 6.8 Hz, 1H), 3.30 (dd, *J* = 6.4, 4.0 Hz, 1H), 3.00 (d, *J* = 18.8 Hz, 1H), 2.90 (s, 1H), 2.54 (dd, *J* = 12.0, 4.4 Hz, 1H), 2.42 (s, 3H), 2.34 (m, 2H), 1.96 (m, 4H), 1.79 (dd, *J* = 12.4, 2.0 Hz, 1H), 0.96 (s, 9H), 0.18 (s, 3H), 0.16 (s, 6H). ^13^C NMR (100 MHz, CDCl_3_) *δ* 169.7, 148.1, 137.4, 133.4, 130.5, 129.0, 127.5, 121.5, 118.9, 92.3, 59.2, 49.7, 47.0, 44.3, 43.2, 40.6, 36.4, 25.9 (3), 23.4, 20.4, 18.5, 4.42, 4.38; MS (ESI): *m*/*z* 441.2 [M + H]; HRMS (ESI) *m*/*z* calcd for C_25_H_37_N_2_O_3_Si 441.2568; found 441.2569.

*N-((4R,4aR,7R,7aR,12bS)-9-((tert-Butyldimethylsilyl)oxy)-3-methyl-11-nitro-2,3,4,4a,7,7a-hexahydro-1H-4,12-methanobenzofuro[3,2-e]isoquinolin-7-yl)acetamide* (**12**). To a solution of TBS ether **11** (1.46 g, 3.3 mmol) in AcOH (20 mL) was added bismuth (III) nitrate pentahydrate (1.93 g, 4.2 mmol) and the mixture was stirred at room temperature overnight. The solvent was evaporated, and the residue was basified with NH_4_OH. The mixture was extracted with CH_2_Cl_2_ (3 × 30 mL) and the combined extracts were washed with brine and dried over anhydrous Na_2_SO_4_. After filtration and removal of solvent in vacuo, the crude product was purified by flash chromatography (CHCl_3_:MeOH:28% NH_4_OH, 95:4.5:0.5) to give **12** (0.8 g, 50.0%) as a brown oil. [α]_D_^20^ −107.2° (*c* 1.0, CHCl_3_); ^1^H NMR (400 MHz, CDCl_3_) *δ* 7.60 (s, 1H), 5.83 (m, 1H), 5.66 (td, *J* = 10.0, 2.0 Hz, 2H), 4.86 (s, 1H), 4.40 (t, *J* = 6.4 Hz, 1H), 3.47 (d, *J* = 20.4 Hz, 1H), 3.33 (m, 1H), 2.94 (s, 1H), 2.60 (dd, *J* = 20.4, 2.0 Hz, 1H), 2.54 (dd, *J* = 12.4, 4.0 Hz, 1H), 2.40 (s, 3H), 2.23 (td, *J* = 12.4, 3.6 Hz, 1H), 2.02 (td, *J* = 12.4, 4.8 Hz, 1H), 1.96 (s, 3H), 1.74 (d, *J* = 10.8 Hz, 1H), 0.94 (s, 9H), 0.18 (s, 6H). ^13^C NMR (100 MHz, CDCl_3_) *δ* 170.0, 153.7, 139.4, 137.5, 133.4, 132.1, 129.1, 126.4, 119.6, 93.8, 58.1, 49.2, 46.2, 44.4, 43.2, 39.3, 36.3, 25.6, 23.2, 21.0, 18.4, 4.54, 4.48; MS (ESI) *m*/*z* 486.2 [M + H]; HRMS (ESI) *m*/*z* calcd for C_25_H_36_N_3_O_5_Si 486.2419; found 486.2420.

*N-((4R,4aR,7R,7aR,12bS)-9-methoxy-3-methyl-2,3,4,4a,7,7a-hexahydro-1H-4,12-methanobenzofuro[3,2-e]isoquinolin-7-yl)acetamide* (**15**): Compound **15** was synthesized with a yield of 74.1% over 3 steps following the procedure of preparing compound **6 [23]**. [α]_D_^20^ −232.0° (*c* 1.02, CHCl_3_); ^1^H NMR (400 MHz, CDCl_3_) *δ* 6.64 (d, *J* = 8.4 Hz, 1H), 6.52 (d, *J* = 8.4 Hz, 1H), 5.80 (m, 1H), 5.59 (dd, *J* = 10.0, 1.6 Hz, 1H), 5.55 (d, *J* = 6.8 Hz, 1H), 4.74 (s, 1H), 4.40 (t, *J* = 6.8 Hz, 1H), 3.83 (s, 3H), 3.30 (m, 1H), 3.20 (d, *J* = 18.4 Hz, 1H), 2.94 (s, 1H), 2.53 (dd, *J* = 12.0, 4.4 Hz, 1H), 2.41 (s, 3H), 2.30 (m, 2H), 2.00 (m, 1H), 1.95 (s, 3H), 1.80 (d, *J* = 10.8 Hz, 1H); ^13^C NMR (100 MHz, CDCl_3_) *δ* 169.8, 146.1, 142.3, 132.8, 130.5, 129.1, 127.2, 118.9, 113.9, 92.5, 59.1, 56.8, 49.9, 47.0, 44.1, 43.2, 40.4, 36.2, 23.3, 20.3; MS (ESI): *m*/*z* = 341.2 [M + H]^+^. HRMS (ESI) *m*/*z* calcd for C_20_H_25_N_3_O_2_ 341.1860; found, 341.1860.

*N-((4R,4aR,7R,7aR,12bS)-9-Methoxy-3-methyl-11-nitro-2,3,4,4a,7,7a-hexahydro-1H-4,12-methanobenzofuro[3,2-e]isoquinolin-7-yl)acetamide* (**16**): To a solution of methyl ether **15** (1.2 g, 3.5 mmol) in AcOH (60 mL) was added bismuth(III) nitrate pentahydrate (2.04 g, 4.2 mmol) and the mixture was stirred at room temperature overnight. The solvent was evaporated and the residue was basified with NH_4_OH. The mixture was diluted with CH_2_Cl_2_ (100 mL) and filtered on a pad of celite. The two-layer filtrate was separated, and the aqueous layer was extracted with CH_2_Cl_2_ (3 × 30 mL) and the combined extracts were washed with brine and dried over anhydrous Na_2_SO_4_. After filtration and evaporation, the crude product was purified by flash chromatography (CHCl_3_:MeOH:NH_4_OH, 95:4.5:0.5) to yield **16** (1.19 g, 88.2%) as a yellow foam. A sample was crystallized from MeOH for analysis. ^1^H NMR (400 MHz, CDCl_3_) *δ* 7.65 (s, 1H), 5.84 (m, 1H), 5.78 (d, *J* = 6.8 Hz, 1H), 5.64 (dd, *J* = 10.0, 1.6 Hz, 1H), 4.91 (s, 1H), 4.40 (t, *J* = 6.4 Hz, 1H), 3.86 (s, 3H), 3.48 (d, *J* = 20.0 Hz, 1H), 3.34 (m, 1H), 2.97 (s, 1H), 2.61 (dd, *J* = 20.4, 6.0 Hz, 1H), 2.53 (dd, *J* = 12.0, 4.0 Hz, 1H), 2.39 (s, 3H), 2.21 (td, *J* = 12.4, 3.6 Hz, 1H), 2.02 (td, *J* = 12.4, 4.8 Hz, 1H), 1.96 (s, 3H), 1.76 (d, *J* = 12.6 Hz, 1H); ^13^C NMR (100 MHz, CDCl_3_) *δ* 170.0, 151.6, 142.1, 139.4, 133.0, 131.8, 129.2, 126.3, 111.0, 94.1, 58.1, 56.6, 49.3, 46.2, 44.2, 43.1, 39.3, 36.2, 23.2, 21.0; MS (ESI): *m*/*z* = 386.2 [M + H]^+^. HRMS (ESI) *m*/*z*: [M + H]^+^ calcd for C_20_H_24_N_3_O_5_ 386.1710; found, 386.1713; Anal. Calcd. for C_20_H_23_N_3_O_5_•1.25 CH_3_OH: C, 59.99; H, 6.63; N, 9.88. Found C, 60.11; H, 6.71; N, 10.04.

*N-((4R,4aR,7R,7aR,12bS)-11-Amino-9-methoxy-3-methyl-2,3,4,4a,7,7a-hexahydro-1H-4,12-methanobenzofuro[3,2-e]isoquinolin-7-yl)acetamide* (**17**): To a solution of nitro compound **16** (1.0 g, 2.6 mmol) in EtOH (15 mL) was added a solution of NaOH (7.5 mL, 0.31 g, 7.8 mmol). Formamidinesulfinic acid (FSA, 0.84 g, 7.8 mmol) was dissolved into a solution of NaOH (7.5 mL, 0.31 g, 7.8 mmol) and the solution was transferred to the alkaline solution of **16**. The solution was heated up to 90 °C for 2 h. The solvent was evaporated, and the aqueous layer was extracted with CH_2_Cl_2_ (3 × 30 mL). The combined extracts were washed with brine and dried over anhyd Na_2_SO_4_. After filtration and evaporation, the crude product was purified by flash chromatography (CHCl_3_:MeOH:NH_4_OH, 85:13.5:1.5) to give **17** (0.728 g, 78.8%) as an off-white foam. ^1^H NMR (400 MHz, CDCl_3_) *δ* 6.06 (s, 1H), 5.74 (m, 1H), 5.58 (d, *J* = 7.2 Hz, 1H), 5.54 (dd, *J* = 9.6, 1.6 Hz, 1H), 4.64 (s, 1H), 4.33 (t, *J* = 6.4 Hz, 1H), 3.77 (s, 3H), 3.35 (m, 3H), 2.89 (s, 1H), 2.69 (d, *J* = 18.4, 6.0 Hz, 1H), 2.52 (dd, *J* = 12.0, 4.0 Hz, 1H), 2.39 (s, 3H), 2.30 (td, *J* = 12.4, 3.6 Hz, 1H), 2.05 (dd, *J* = 14.0, 6.0 Hz, 1H), 1.95 (td, *J* = 12.4, 4.8 Hz, 1H), 1.92 (s, 3H), 1.79 (dd, *J* = 12.6, 2.0 Hz, 1H); ^13^C NMR (100 MHz, CDCl_3_) *δ* 169.7, 142.7, 139.0, 137.1, 132.2, 131.2, 129.0, 112.4, 101.1, 92.2, 58.8, 56.8, 50.2, 47.0, 44.3, 43.2, 40.2, 35.9, 23.3, 17.1; MS (ESI): *m*/*z* 356.2 [M + H]^+^. HRMS (ESI) *m*/*z*: calcd for C_20_H_26_N_3_O_3_ 356.1969; found, 356.1968.

*N-((4R,4aR,7S,7aR,12bS)-9-Methoxy-3-methyl-2,3,4,4a,7,7a-hexahydro-1H-4,12-methanobenzofuro[3,2-e]isoquinolin-7-yl)acetamide* (**20**). To a stirred suspension of codeinone oxime [26] (**18**, 3.52 g, 11.27 mmol) in anhydrous toluene (225 mL) was added Red-Al solution 65% in toluene, 56.34 mmol, 17.18 mL, 5 equiv). The solids dissolved upon addition of the Red-Al solution. The solution was heated to 70 °C for 1.5 h until the starting material was consumed as indicated by TLC and mass spectrometry. The solution was cooled to 25 °C and 5% NaOH solution (100 mL) was added. The mixture was stirred until two homogenous layers formed. The organic layer was separated, and the aqueous layer was extracted with CHCl_3_ (3 × 50 mL). The combined organic extracts were dried over Na_2_SO_4_, filtered, and concentrated in vacuo to give intermediate **19** ((4*R*,4a*R*,7*S*,7a*R*,12b*S*)-9-methoxy-3-methyl-2,3,4,4a,7,7a-hexahydro-1*H*-4,12-methanobenzofuro[3,2-*e*]isoquinolin-7-amine) as a pale yellow oil. The crude amine was dissolved in CHCl_3_ (112 mL), and triethylamine (3.42 g, 33.81 mmol, 4.71 mL, 3 equiv) and 4-dimethylaminopyridine (0.14 g, 1.12 mmol, 0.1 equiv were added sequentially under N_2_. The solution was cooled to 0 °C and acetic anhydride (2.30 g, 22.53 mmol, 2.13 mL, 2 equiv) was added in one portion. The solution was stirred for 16 h, warming to 25 °C. The solution was washed with saturated aqueous NaHCO_3_ (25 mL, H_2_O (25 mL), dried over Na_2_SO_4_, filtered, and solvent removed in vacuo to give a yellow oil. The oil was purified by column chromatography on SiO_2_ (CHCl_3_:MeOH:28% NH_4_OH, 99:0.9:0.1 to 95:94.5:0.5) to give **20** as a clear oil (2.90 g, 75%). [α]_D_^20^ −1.91° (*c* 0.59, MeOH); ^1^H NMR (400 MHz, CDCl_3_) *δ* 6.60 (d, *J* = 8.4 Hz, 1H), 6.51 (d, *J* = 8.4 Hz, 1H), 6.35 (d, *J* = 8.7 Hz, 1H), 5.43 (d, *J* = 9.7 Hz, 1H), 5.33 (dt, *J* = 9.7, 2.6 Hz, 1H), 4.78 (dd, *J* = 6.2, 1.0 Hz, 1H), 4.53 (dtd, *J* = 8.7, 5.7, 2.9 Hz, 1H), 3.76 (s, 3H), 3.30 (dd, *J* = 6.1, 3.2 Hz, 1H), 2.99 (d, *J* = 18.6 Hz, 1H), 2.68 (quintet, *J* = 2.7 Hz, 1H), 2.52 (dd, *J* = 12.2, 4.1 Hz, 1H), 2.38 (s, 3H), 2.34 (dt, *J* = 12.2, 6.1 Hz, 1H), 2.25 (dt, *J* = 17.2, 7.9 Hz, 1H), 2.01 (dt, *J* = 13.7, 7.4 Hz, 1H), 1.99 (s, 3H), 1.77 (dd, *J* = 12.6, 1.6 Hz, 1H); ^13^C NMR (100 MHz, CDCl_3_) *δ* 169.7, 146.4, 141.9, 130.8, 130.6, 130.0, 127.4, 119.3, 112.5, 90.8, 58.7, 56.1, 47.1, 46.4, 43.5, 43.1, 40.8, 35.7, 23.3, 20.4; HRMS (ESI) (*m*/*z*) [M+H] calcd for C_20_H_25_N_2_O_3_ 341.1865, found 341.1863. Anal calcd for C_20_H_24_N_2_O_3_•0.45 CHCl_3_: C, 62.32; H, 6.25; N, 7.11; Found C, 62.01; H, 6.61; N, 7.02. 

*N-((4R,4aR,7S,7aR,12bS)-9-Methoxy-3-methyl-11-nitro-2,3,4,4a,7,7a-hexahydro-1H-4,12-methanobenzofuro[3,2-e]isoquinolin-7-yl)acetamide* (**21**). Amide 20 (2.97 g, 8.72 mmol) was dissolved in acetic acid (174 mL) under N_2_. The solution was cooled to 10 °C and freshly ground bismuth(III) nitrate pentahydrate (5.29 g, 10.90 mmol, 1.25 equiv) was added in one portion. The solution was stirred for 16 h, warmed to 25 °C then concentrated in vacuo to give a yellow residue. The residue was made basic (pH > 9) with 28% NH_4_OH, and filtered through celite to remove the solid precipitate, rinsing with 10% MeOH:CHCl_3_. The organic layer was separated and the aqueous layer was extracted with 10% MeOH:CHCl_3_ (4 × 50 mL). The combined organic layers were dried over Na_2_SO_4_, filtered, and concentrated in vacuo to give a yellow solid. The solid was triturated with 1:1 acetone: Et_2_O, filtered, and dried to give **21** as a yellow solid (1.52 g, 45%). ^1^H NMR (400 MHz, CDCl_3_) *δ* 7.69 (s, 1H), 6.14 (d, *J* = 8.7 Hz, 1H), 5.51 (dd, *J* = 9.7, 2.9 Hz, 1H), 5.44 (dt, *J* = 9.7, 2.6 Hz, 1H), 4.99 (dd, *J* = 6.0, 0.8 Hz, 1H), 4.69 (ddd, *J* = 8.6, 5.8, 2.8 Hz, 1H), 3.92 (s, 3H), 3.53 (d, *J* = 20.3 Hz, 1H), 3.42 (dd, *J* = 6.0, 3.2 Hz, 1H), 2.76 (q, *J* = 2.6 Hz, 1H), 2.65 (dd, *J* = 20.2, 6.1 Hz, 1H), 2.60 (dd, *J* = 10.4, 4.4 Hz, 1H), 2.46 (s, 3H), 2.32 (td, *J* = 12.3, 3.4 Hz, 1H), 2.13 (dd, *J* = 12.5, 5.1 Hz, 1H), 2.09 (s, 3H), 1.82 (dd, *J* = 12.8, 1.7 Hz, 1H); ^13^C NMR (100 MHz, CDCl_3_) *δ* 169.7, 151.8, 141.8, 139.9, 132.2, 130.8, 130.3, 126.5, 110.2, 92.8, 57.8, 56.3, 47.2, 45.7, 43.8, 43.1, 40.1, 35.7, 23.3, 21.3; HRMS (ESI) (*m*/*z*) [M+H] calcd for C_20_H_24_N_3_ 386.1716, found 386.1712. Anal calcd for C_20_H_23_N_3_O_5_•0.25 H_2_O: C, 61.61; H, 6.07; N, 10.78; found C, 61.67; H, 6.06; N, 10.67.

*N-((4R,4aR,7S,7aR,12bS)-9-Hydroxy-3-methyl-11-nitro-2,3,4,4a,7,7a-hexahydro-1H-4,12-methanobenzofuro[3,2-e]isoquinolin-7-yl)acetamide* (**22**). Under nitrogen, amide **21** (0.50 g, 1.41 mmol) in anhyd CH_2_Cl_2_ (28 mL) was cooled to −78 °C and BBr_3_ (2.11 g, 8.44 mmol, 0.80 mL, 6 equiv) was added dropwise. The solution was stirred for 16 h, warming to 25 °C. The solution was cooled to 0 °C and MeOH (10 mL) was added to neutralize the remaining BBr_3_. The solution was concentrated in vacuo and then stripped with MeOH (20 mL × 3) to give a yellow solid. The solid was dissolved in MeOH and purified via silica gel chromatography (CHCl_3_:MeOH:28% NH_4_OH, 92:1.8:0.2 to 90:9:1) to give **22** as a yellow solid (0.40 g, 77%).^1^H NMR (400 MHz; CDCl_3_) *δ* 7.64 (s, 1H), 6.74 (d, *J* = 8.7 Hz, 1H), 5.53 (d, *J* = 9.8 Hz, 1H), 5.45 (dt, *J* = 9.5, 2.4 Hz, 1H), 5.00 (d, *J* = 5.8 Hz, 1H), 4.65 (dt, *J* = 5.6, 2.9 Hz, 1H), 3.50 (m, 2H), 2.81 (t, *J* = 2.2 Hz, 1H), 2.73–2.66 (m, 2H), 2.49 (s, 3H), 2.40–2.39 (m, 1H), 2.16–2.12 (m, 1H), 2.09 (s, 3H), 1.83 (d, *J* = 11.4 Hz, 1H); ^13^C NMR (100 MHz; CDCl_3_) *δ* 170.7, 151.5, 139.9, 139.2, 132.0, 130.4, 130.2, 124.5, 114.9, 92.0, 58.0, 47.5, 45.7, 43.6, 42.8, 39.5, 35.1, 23.1, 21.6.

*N-((4R,4aR,7S,7aR,12bS)-9-((tert-Butyldiphenylsilyl)oxy)-3-methyl-11-nitro-2,3,4,4a,7,7a-hexahydro-1H-4,12-methanobenzofuro[3,2-e]isoquinolin-7-yl)acetamide* (**23**). Phenol **22** (0.20 g, 0.54 mmol) was dissolved in CHCl_3_ (5 mL) under N_2_, and imidazole (0.15 g, 2.15 mmol, 4 equiv) and *tert*-butyldimethylsilyl chloride (0.30 g, 1.10 mmol, 0.28 mL, 2 equiv) were added sequentially. The solution was heated to 50 °C until TLC indicated that the starting material was consumed. The solution was cooled, concentrated and purified via silica gel chromatography (CHCl_3_:MeOH:28% NH_4_OH, 99:0.9:0.1 to 95:94.5:0.5) to give **23** as a yellow oil that solidified upon standing (0.274 g, 83%).[α]_D_^20^ −0.545° (*c* 0.46, MeOH); ^1^H NMR (400 MHz; CDCl_3_) *δ* 7.70–7.63 (m, 5H), 7.45–7.41 (m, 2H), 7.39–7.34 (m, 4H), 5.41 (d, *J* = 8.9 Hz, 1H), 5.36 (dt, *J* = 9.7, 2.7 Hz, 1H), 5.24 (d, *J* = 9.7 Hz, 1H), 4.64 (dd, *J* = 5.9, 0.9 Hz, 1H), 4.48 (ddd, *J* = 8.7, 5.9, 2.9 Hz, 1H), 3.45 (d, *J* = 20.3 Hz, 1H), 3.34 (dd, *J* = 6.0, 3.2 Hz, 1H), 2.65 (t, *J* = 2.7 Hz, 1H), 2.56 (td, *J* = 17.1, 5.7 Hz, 2H), 2.41 (s, 3H), 2.23 (td, *J* = 12.4, 3.5 Hz, 1H), 1.96 (td, *J* = 12.5, 5.1 Hz, 1H), 1.76 (s, 3H), 1.54 (dd, *J* = 12.7, 1.6 Hz, 1H), 1.14 (s, 9H); ^13^C NMR (100 MHz; CDCl_3_) *δ* 169.4, 153.8, 139.7, 137.0, 135.2, 132.6, 132.4, 130.3, 130.3, 130.2, 128.0, 126.6, 118.992.1, 57.7, 47.0, 45.6, 43.7, 43.1, 40.0, 35.5, 26.4, 23.0, 21.3, 19.6; HRMS (ESI) (*m*/*z*) [M+H] calcd for C_35_H_40_N_3_O_5_Si 610.2737, found 610.2740. Anal calcd for C_35_H_39_N_3_O_5_Si•0.25 CHCl_3_: C, 66.19; H, 6.19; N, 6.57, found C, 66.07; H, 6.28; N, 6.48.

*N-((4R,4aR,7S,7aR,12bS)-7-acetamido-9-((tert-butyldiphenylsilyl)oxy)-3-methyl-2,3,4,4a,7,7a-hexahydro-1H-4,12-methanobenzofuro[3,2-e]isoquinolin-11-yl)-3-(tritylthio)propanamide* (**25**)**.** A solution of amide **23** (0.165 g, 0.271 mmol), EtOH (5.40 mL), and stannous chloride dihydrate (0.305 g, 1.35 mmol, 5 equiv) was heated to 60 °C for 10 min, then sodium borohydride (0.005 g, 0.135 mmol, 0.5 equiv) was added in one portion. The mixture was stirred for 2 h, then the temperature was increased to 65 °C and a second portion of sodium borohydride (0.005 g, 0.135 mmol, 0.5 equiv) was added. The solution was stirred at 65 °C for 16 h, at which point TLC analysis indicated that the starting material had been consumed. The solution was concentrated in vacuo and the excess hydride was neutralized by addition of cold 5% NaHCO_3_ solution (10 mL). The aqueous solution was extracted with 10% MeOH:CHCl_3_ (3 × 10 mL), and the combined organic extracts were dried over Na_2_SO_4_, filtered, and concd in vacuo to give the crude intermediate aniline (**24**) as clear oil that was used without purification in the subsequent reaction. The crude aniline **24** was dissolved in CHCl_3_ (5 mL), and triethylamine (0.109 g, 1.08 mmol, 0.150 mL, 4 equiv) and 3-(tritylthio)propanoic acid (0.189 g, 0.541 mmol, 2 equiv) were added sequentially. The solution was cooled to 0 °C and TBTU (0.347 g, 1.08 mmol, 4 equiv) was added in one portion. The solution was stirred for 16 h, warming to 25 °C, then washed with H_2_O (2 × 5 mL). The combined aqueous layers were back extracted with CHCl_3_ (2 × 5 mL). The combined organic extracts were dried over Na_2_SO_4_, filtered, and concentrated in vacuo to give a yellow oil. The oil was purified via silica gel chromatography (CHCl_3_:MeOH:28% NH_4_OH, 99:0.9:0.1 to 95:94.5:0.5) to give **25** as a clear oil (0.116 g, 46%).^1^H NMR (400 MHz; CDCl_3_) *δ* 7.70 (dd, *J* = 13.3, 6.9 Hz, 4H), 7.44–7.27 (m, 15H), 7.21 (dd, *J* = 16.9, 9.8 Hz, 5H), 6.86 (s, 1H), 6.76 (s, 1H), 5.59 (d, *J* = 8.8 Hz, 1H), 5.27–5.20 (m, 2H), 4.53 (d, *J* = 6.0 Hz, 1H), 4.40 (d, *J* = 6.0 Hz, 1H), 3.27 (dd, *J* = 5.4, 3.0 Hz, 1H), 2.81 (d, *J* = 18.5 Hz, 1H), 2.62–2.48 (m, 4H), 2.33–2.26 (m, 4H), 2.13–2.08 (m, 2H), 1.95 (m, 2H), 1.71 (s, 3H), 1.62 (d, *J* = 11.9 Hz, 1H), 1.12 (s, 9H); ^13^C NMR (100 MHz; CDCl_3_) *δ* 169.5, 168.8, 146.2, 144.6, 136.8, 135.30, 135.26, 133.5, 133.2, 131.2, 130.3, 129.94, 129.85, 129.5, 127.96, 127.80, 127.6, 127.3, 126.7, 121.8, 115.8, 90.5, 67.0, 58.4, 47.0, 46.2, 43.7, 43.1, 40.7, 36.2, 35.6, 27.9, 26.5, 26.4, 23.0, 19.7, 18.3.

*(4R,4aR,7S,7aR,12bS)-7-(Benzylamino)-3-methyl-2,3,4,4a,5,6,7,7a-octahydro-1H-4,12-methanobenzofuro[3,2-e]isoquinolin-9-ol* (**26**). To a solution of hydromorphone [31,32] (2.28 g, 8.0 mmol) in DCE (60 mL) was added benzylamine (0.96 mL, 8.8 mmol, 1.1 equiv), acetic acid (0.91 mL, 16.0 mmol, 2 equiv), and NaBH(OAc)_3_ (2.54 g, 1.2 mmol, 1.5 equiv), respectively. The mixture was stirred at room temperature for 24 h. Water (20 mL) was added to the reaction mixture, and it was basified to a pH ~8 with 28% NH_4_OH. The organic layer was separated, and the aqueous layer was extracted with CHCl_3_ (3 × 25 mL). The combined organic layers were washed with brine, dried over MgSO_4_, filtered, concentrated in vacuo, and the product was purified through silica gel chromatography (CHCl_3_:MeOH:28% NH_4_OH, 90:9:1)) to give **26** (2.85 g, 95% yield) as a light-yellow foam. [α]^20^_D_ −184.3° (*c* 1.9, CHCl_3_); ^1^H NMR (400 MHz, CDCl_3_) *δ* 7.38–7.19 (m, 5H), 6.65 (d, *J* = 8.0 Hz, 1H), 6.50 (d, *J* = 8.0 Hz, 1H), 4.63 (d, *J* = 3.4 Hz, 1H), 3.90 (dd, *J* = 41.7, 13.0 Hz, 2H), 3.04 (dd, *J* = 6.1, 2.5 Hz, 1H), 2.91 (d, *J* = 18.6 Hz, 1H), 2.71–2.75 (m, 1H), 2.49 (dd, *J* = 12.0, 4.2 Hz, 1H), 2.36 (s, 3H), 2.35–2.24 (m, 2H), 2.16–2.12 (m, 1H), 1.84 (td, *J* = 12.4, 5.0 Hz, 1H), 1.68–1.43 (m, 3H), 0.92–0.77 (m, 2H); ^13^C NMR (400 MHz, CDCl_3_) *δ* 145.9, 139.2, 138.0, 129.6, 128.5, 128.4, 128.4, 127.1, 126.1, 119.2, 118.0, 89.1, 59.6, 53.6, 50.4, 46.1, 43.0, 42.7, 38.0, 36.0, 21.4, 20.7, 19.9; HRMS (ESI) *m*/*z* 377.2224 [C_24_H_29_N_2_O_2_ (M+H) requires 377.2229].

*N-((4R,4aR,7S,7aR,12bS)-9-hydroxy-3-methyl-2,3,4,4a,5,6,7,7a-octahydro-1H-4,12-methanobenzofuro[3,2-e]isoquinolin-7-yl)acetamide* (**29**). To a solution of **26** (3.0 g, 7.9 mmol) in MeOH (100 mL) in a 500 mL hydrogenation bottle was added 10% (*w*/*w*) Pd/C (1.5 g) and 37% HCl (1.5 mL). The reaction was stirred under H_2_ (50 psi) for 4 days at 40 °C. The reaction mixture was filtered through a Celite pad, and concentrated under vacuo to give a yellow solid amine **27** (4*R*,4a*R*,7*S*,7a*R*,12b*S*)-7-amino-3-methyl-2,3,4,4a,5,6,7,7a-octahydro-1*H*-4,12-methanobenzofuro[3,2-*e*]isoquinolin-9-ol). To the intermediate **27**, DCM (25 mL), triethylamine (5.7, 41.1 mmol), and Ac_2_O (5.1 mL, 54 mmol) were added, respectively. The reaction mixture was stirred for 16 h at 40 °C, cooled to room temperature, and H_2_O (30 mL) was added to the reaction mixture. The mixture was basified to pH ~8.5 with 28% NH_4_OH and extracted with CHCl_3_:2-propanol (9:1) (3 × 30 mL). The combined organic layers were concentrated in vacuo to give a thick viscous syrupy amide **28** ((4*R*,4a*R*,7*S*,7a*R*,12b*S*)-7-acetamido-3-methyl-2,3,4,4a,5,6,7,7a-octahydro-1*H*-4,12-methanobenzofuro[3,2-*e*]isoquinolin-9-yl acetate). The syrup was dissolved in MeOH (30 mL) and 6 M NaOH (4 mL) and stirred at room temperature for 1 h. The reaction mixture was concentrated in vacuo, dissolved in CHCl_3_ (40 mL), and extracted with 2 M NaOH (3 × 20 mL). The combined aqueous layers were acidified to pH < 4 with 37%. HCl, and then re-basified to pH ~8.5 with 28% NH_4_OH. The aqueous solution was extracted with CHCl_3_:MeOH (8:1) (3 × 50 mL) and the combined organic layers were dried over MgSO_4_, filtered, and the solvent removed in vacuo to provide **29** (1.92 g, 70% yield) as a light yellow solid. White crystals were obtained after recrystallization in DCM and hexane, mp 137–142 °C. [α]^20^_D_ −167.5° (*c* 1.0, CHCl_3_); ^1^H NMR (400 MHz, CDCl_3_) *δ* 6.68 (d, *J* = 8.1 Hz, 1H), 6.56 (d, *J* = 8.1 Hz, 1H), 6.10 (d, *J* = 9.0 Hz, 1H), 4.62 (d, *J* = 4.6 Hz, 1H), 4.28–4.21 (m, 1H), 3.14 (dd, *J* = 5.9, 2.5 Hz, 1H), 2.98 (d, *J* = 18.7 Hz, 1H), 2.55 (dd, *J* = 12.1, 3.9 Hz, 1H), 2.47–2.37 (m, 4H), 2.35–2.27 (m, 2H), 2.03–1.93 (m, 4H), 1.73–1.64 (m, 2H), 1.51–1.43 (m, 1H), 1.10 –1.04 (m, 1H), 0.94–0.83 (m, 1H); ^13^C NMR (400 MHz, CDCl_3_) *δ* 169.9, 145.4, 138.0, 129.7, 126.4, 119.5, 117.1, 89.7, 59.6, 46.7, 46.2, 42.8, 42.7, 37.0, 36.4, 23.3, 22.5, 20.3, 20.0; HRMS (ESI) *m*/*z* 329.1861 [C_19_H_25_N_2_O_3_ (M+H) requires 329.1865].

*N-((4R,4aR,7S,7aR,12bS)-9-((tert-butyldimethylsilyl)oxy)-3-methyl-2,3,4,4a,5,6,7,7a-octahydro-1H-4,12-methanobenzofuro[3,2-e]isoquinolin-7-yl)acetamide* (**30**). To a solution of **29** (1.0 g, 3.0 mmol) in DCM (25 mL) was added 1*H*-imidazole (0.23 g, 3.3 mmol, 1.1 equiv) and *tert*-butyldimethylsilyl chloride (TBS-Cl, 0.60 g, 4.0 mmol, 1.3 equiv) and the mixture was stirred overnight at room temperature. The reaction was quenched with saturated NaCO_3_H, and extracted with CHCl_3_ (3 × 25 mL). The combined organic layers were washed with brine, dried over MgSO_4_, filtered, the solvent removed in vacuo, and the product was purified through silica gel chromatography (CHCl_3_:MeOH:28% NH_4_OH, 90:9:1) to give **30** (1.22 g, 91% yield) as a white solid, mp 176–178 °C. [α]^20^_D_ −146° (*c* 1.1, CHCl_3_); ^1^H NMR (400 MHz, CDCl_3_) *δ* 6.65 (d, *J* = 8.1 Hz, 1H), 6.54 (d, *J* = 8.1 Hz, 1H), 5.81 (d, *J* = 8.7 Hz, 1H), 4.58 (d, *J* = 4.4 Hz, 1H), 4.21–4.08 (m, 1H), 3.08 (dd, *J* = 6.1, 2.7 Hz, 1H), 2.95 (d, *J* = 18.7 Hz, 1H), 2.48 (dd, *J* = 12.2, 4.5 Hz, 1H), 2.43–2.34 (m, 4H), 2.31–2.20 (m, 2H), 1.95 (s, 3H), 1.90 (dd, *J* = 12.4, 5.1 Hz, 1H), 1.80–1.62 (m, 2H), 1.53–1.47 (m, 1H), 1.02 (s, 9H), 0.94–0.84 (m, 2H), 0.23 (s, 3H), 0.18 (s, 3H). ^13^C NMR (400 MHz, CDCl_3_) *δ* 169.1, 148.2, 136.4, 130.3, 128.4, 121.3, 119.2, 89.6, 59.6, 46.8, 46.1, 43.1, 42.8, 37.6, 35.9, 25.6, 23.3, 22.0, 20.4, 19.9, 18.3, −4.2, −4.5. HRMS (ESI) *m*/*z* 443.2725 [C_24_H_29_N_2_O_2_ (M+H) requires 443.2729].

*N-((4R,4aR,7S,7aR,12bS)-9-((tert-butyldimethylsilyl)oxy)-3-methyl-11-nitro-2,3,4,4a,5,6,7,7a-octahydro-1H-4,12-methanobenzofuro[3,2-e]isoquinolin-7-yl)acetamide* (**31**). To a stirred solution of **30** (860 mg, 1.94 mmol) in trifluoroacetic acid (10 mL) at 0 °C was added NaNO_2_ (174 mg, 2.52 mmol, 1.3 equiv). The reaction mixture was stirred for 30 min at 0 °C, slowly basified to pH ~8.5 with 28% NH_4_OH, and extracted with CHCl_3_:2-propanol (8:1) (4 × 25 mL). The combined organic layers were dried over MgSO_4_, filtered, concentrated in vacuo, and the product was purified through silica gel chromatography (CHCl_3_:MeOH:28% NH_4_OH, 90:9:1)) to give **31** (710 mg, 75% yield) as light green foam. [α]^20^_D_ −142.0° (*c* 1.7, CHCl_3_); ^1^H NMR (400 MHz, CDCl_3_) *δ* 7.71 (s, 1H), 5.65 (d, *J* = 8.6 Hz, 1H), 4.78 (dd, *J* = 4.4, 1.1 Hz, 1H), 4.27–4.19 (m, 1H), 3.41 (d, *J* = 20.3 Hz, 1H), 3.15 (dd, *J* = 6.1, 2.9 Hz, 1H), 2.77 (dd, *J* = 20.3, 6.2 Hz, 1H), 2.52 (dd, *J* = 12.4, 4.2 Hz, 1H), 2.42 (s, 3H), 2.32 (td, *J* = 9.4, 2.8 Hz, 1H), 2.20 (td, *J* = 12.3, 3.5 Hz, 1H), 2.05–1.91 (m, 4H), 1.88–1.72 (m, 2H), 1.71–1.51 (m, 2H), 1.03 (s, 9H), 0.99–0.81 (m, 2H), 0.27 (s, 3H), 0.23 (s, 3H); ^13^C NMR (400 MHz, CDCl_3_) *δ* 169.2, 154.0, 139.7, 136.7, 131.9, 127.1, 119.6, 91.5, 58.8, 46.5, 45.4, 43.2, 42.9, 37.7, 35.0, 25.5, 23.3, 21.7, 20.6, 20.2, 18.3, −4.3, −4.5; HRMS (ESI) *m*/*z* 488.2575 [C_25_H_38_N_3_O_5_Si (M+H) requires 488.2581].

*N-((4R,4aR,7S,7aR,12bS)-9-hydroxy-3-methyl-11-nitro-2,3,4,4a,5,6,7,7a-octahydro-1H-4,12-methanobenzofuro[3,2-e]isoquinolin-7-yl)acetamide* (**32**). To a solution of **31** (487 mg, 1.0 mmol) in THF (3 mL) was added 3 M HCl (2 mL), and the mixture was stirred for 1 h at 60 °C. After cooling to room temperature, the reaction mixture was basified to pH~8 with 28%. NH_4_OH and extracted with CHCl_3_:MeOH (5:1) (3 × 20 mL). The combined organic layers were dried over MgSO_4_, filtered, concd in vacuo, and purified through silica gel chromatography (CHCl_3_:MeOH:28% NH_4_OH, 90:9:1)) to give the phenol **32** (354 mg, 9% yield) as an orange solid, mp 170–175 °C. [α]^20^_D_ −187.3° (*c* 2.25, CHCl_3_); HRMS (ESI) *m*/*z* 374.1710 [C_19_H_24_N_3_O_5_ (M+H) requires 374.1716]. ^1^H NMR (400 MHz, CDCl_3_) *δ* 9.63 (br, 1H), 7.75 (s, 1H), 6.40 (d, *J* = 8.3 Hz, 1H), 4.83 (d, *J* = 4.0 Hz, 1H), 4.30 (m, 1H), 3.44 (d, *J* = 20.0 Hz, 1H), 3.30 (s, 1H), 2.87 (d, *J* = 14.5 Hz, 1H), 2.67 (d, *J* = 8.0 Hz, 1H), 2.56–2.40 (m, 4H), 2.38–2.25 (m, 1H), 2.12–2.03 (m, 4H), 1.80–1.71 (m, 2H), 1.59–1.49 (m, 1H), 1.18 – 1.07 (m, 1H), 1.04–0.93 (m, 1H); ^13^C NMR (400 MHz, CDCl_3_) *δ* 170.4, 151.8, 139.9, 139.7, 130.8, 124.0, 115.4, 91.0, 58.9, 46.6, 45.5, 42.7, 42.6, 36.8, 35.0, 23.2, 21.7, 21.1, 20.1.

*N-((4R,4aR,7R,7aR,12bS)-11-amino-9-hydroxy-3-methyl-2,3,4,4a,5,6,7,7a-octahydro-1H-4,12-methanobenzofuro[3,2-e]isoquinolin-7-yl)acetamide* (**34**). To a solution of acetamide **8** (0.25 g, 0.67 mmol) in MeOH (50 mL) was added 10% Pd/C (50 mg). The mixture was hydrogenated under a hydrogen balloon overnight, filtered on a pad of celite and the filtrate was concentrated in vacuo. The product was purified by flash chromatography (CHCl_3_:MeOH:28% NH_4_OH, 80:18:2) to give **34** (105 mg, 45.6%) as a white foam. [α]_D_^20^ −147.1° (*c* 1.0, MeOH); ^1^H NMR (400 MHz, CDCl_3_+CD_3_OD) *δ* 5.99 (s, 1H), 4.08 (d, *J* = 8.0 Hz, 1H), 3.46 (m, 1H), 3.25 (s, 1H), 3.02 (s, 1H), 2.61 (d, *J* = 18.0 Hz, 1H), 2.39 (d, *J* = 11.2 Hz, 1H), 2.25 (s, 3H), 2.08 (t, *J* = 12.0 Hz, 1H), 1.98 (m, 2H), 1.81 (s, 3H), 1.62 (m, 2H), 1.52 (d, *J* = 12.0 Hz, 1H), 1.35 (d, *J* = 12.4 Hz, 1H), 1.08 (m, 1H), 0.88 (m, 1H). ^13^C NMR (100 MHz, CDCl_3_+CD_3_OD) *δ* 171.3, 140.5, 137.0, 136.0, 129.7, 110.8, 104.5, 92.6, 59.0, 51.4, 47.1, 43.3, 42.3, 41.8, 34.8, 28.7, 23.8, 22.7, 16.7; MS (ESI): *m*/*z* = 344.2 [M + H]. HRMS (ESI) *m*/*z*: calcd for C_19_H_26_N_3_O_3_ 344.1969; found, 344.1969.

*X-ray crystallographic data for compound***16.** Single-crystal X-ray diffraction data on compound **16** (deposition CCDC 1554589) were collected using Cu Kα radiation and a Bruker PLATINUM 135 CCD area detector. The crystal was prepared for data collection by coating with high viscosity microscope oil. The oil-coated crystal was mounted on a micro-mesh mount (MiteGen, Inc.) and transferred to the diffractometer and a data set collected at 150 °K. The 0.373 × 0.343 × 0.281 mm^3^ crystal was monoclinic in space group P2_1_, with unit cell dimensions a = 7.39130(10) Å, b = 9.4942(2) Å, c = 13.1498(3) Å, *α* = 90°, *β* = 92.8700(10)°, and *γ* = 90°. Data were 94.5% complete to 67.679° θ (~0.83 Å) with an average redundancy of 3.00. The final anisotropic full-matrix least-squares refinement on F^2^ with 257 variables converged at R_1_ = 3.94%, for the observed data and wR2 = 9.13% for all data. The structure was solved by direct methods and refined by full-matrix least squares on F^2^ values using the programs found in the SHELXL suite (Bruker, SHELXL v2014.7, 2014, Bruker AXS Inc., Madison, WI, USA). Corrections were applied for Lorentz, polarization, and absorption effects. Parameters refined included atomic coordinates and anisotropic thermal parameters for all non-hydrogen atoms. The H atoms were included using a riding model. Complete information on data collection and refinement is available in the Supplementary Material.

### 4.2. Preparation of TT–Hapten Conjugates

Each of the TT–hapten conjugates (TT-**1**, TT-**2**, TT-**3**, and TT-**4**) was separately made using the procedures reported previously [18,23,24,27,33]. Briefly, TT was incubated with the SM(PEG)_2_ cross-linker at a 1:1600 molar ratio for 2 h. Excess linker was removed using a Zeba spin column, and the protein content of the flow-through was quantified using a BCA assay kit. The excess haptens were removed by repeated dialysis against PBS, pH 7.4, at 4 °C. The TT–hapten conjugates were sterile filtered and quantified using the BCA protein assay. The number of haptens attached per TT molecule in each conjugate was measured using matrix assisted laser desorption/ionization time-of-flight (MALDI-TOF) mass spectrometry, generally, approximately 25–35 haptens per TT molecule were obtained (data not shown).

### 4.3. Immunizations and Animal Challenge

All animal studies were conducted under an approved animal use protocol in an Association for Assessment and Accreditation of Laboratory Animal Care International (AAALACI)-accredited facility in compliance with the Animal Welfare Act and other federal statutes and regulations relating to animals. Experiments involving animals adhered to the principles stated in the Guide for the Care and Use of Laboratory Animals, 8th edition [34]. Female BALB/c mice, 6–7 weeks of age (*n* = 10 per group, for a total of 50 mice) were immunized intramuscularly (i.m.) on weeks 0, 3, and 6 using 50 μL of the vaccine formulation. This dose contained 10 μg of TT−hapten (based on the protein content of the protein−hapten conjugate), 20 μg of PHAD^®^ in ALF43 [18,23], and 30 μg of aluminum in aluminum hydroxide (Alhydrogel^®^) in DPBS pH 7.4. Mice were bled for ELISA assay at weeks 0, 3, 6, and 8. Challenge studies were performed at week 10.

Mice received 1.0 mg/kg of heroin (s.c.), and this route has been used previously to evaluate anti-opioid vaccines [27,28]. Antinociceptive effects were assessed 15 min after each heroin injection using the hot plate assay [35]. This assay involved placing the mouse on a hot plate set at 54 °C. The latency to a nociceptive response was recorded, defined as the time elapsed until the mouse licked or flicked its hind paw. The latency times were measured with a cutoff time of 30 sec to prevent injury. Antinociception, measured as %MPE, was calculated using Equation (1):

(1) $$\%\mathrm{MPE}=\frac{\mathrm{Post}\;\mathrm{heroin}\;\mathrm{injection}\;\mathrm{latency}\;\mathrm{time}-\mathrm{baseline}\;\mathrm{latency}\;\mathrm{time}}{\mathrm{Cutoff}\;\mathrm{latency}-\mathrm{baseline}\;\mathrm{latency}\;\mathrm{time}}\times \;100$$

### 4.4. Drug Binding Analysis

Drug binding was measured using ED–LC–MS/MS as described previously [22,27,28]. Briefly, mice sera were diluted with 0.05% BSA in DPBS, pH 7.4 (ED buffer) containing 3–5 mg/mL NaF and 5 nM of a drug. The following drugs were tested: heroin, 6-A.M., morphine, methadone, naloxone, buprenorphine, and methadone. An aliquot (100 µL) was seeded into sample chambers of the ED plate and the buffer chamber was filled with 300 µL of ED buffer. The plate was incubated at 4 °C and 300 rpm for 24 h in a thermomixer. Aliquots (90 µL) from sample and buffer chambers were pipetted out and analyzed by LC–MS/MS. 

### 4.5. Data Analysis

Graphical and statistical analyses were performed using Prism 9 (GraphPad Software, San Diego, CA, USA). All results were reported as the mean ± standard error of the mean (sem). The chemical structures and 3D optimization were made using ChemDraw 18.1. The built-in MM2 method was used for geometry optimization and energy minimization. 

## 5. Conclusions

In this study, we described the synthesis of haptens **1**–**4**, (Figure 1), produced the individual TT–hapten conjugates and evaluated their efficacy both in vivo and in vitro. We showed that only haptens **2** and **3** can induce protection against the effects of heroin in vivo. The epimeric congeners of these haptens, haptens **1** and **4**, failed to protect mice from the effects of heroin. We also showed that the in vivo efficacy is consistent with the results of the in vitro drug sequestration assay. Only TT-**2** and TT-**3** yielded antibodies that bound heroin and 6-AM. None of the TT–hapten conjugates induced antibodies that cross-reacted with morphine, methadone, naloxone, or naltrexone, and only TT-**3** interacted weakly with buprenorphine. These results highlight the importance of judicious hapten design toward effective heroin vaccines.

## Data Availability

Data are available upon request to the Corresponding Authors.

## Supplementary Materials

The following are available online. ^1^H and ^13^C NMRs of novel compounds and X-ray crystallographic data for compound **16** can be found in the Supplementary Data.

## Sample Availability

Some of the compounds may be available in mg quantities upon request from the Corresponding Authors.
