# Supplementary material for: Design, Synthesis, and In Vivo Evaluation of C1-Linked 4,5-Epoxymorphinan Haptens for Heroin Vaccines
Source: Molecules. 2022 Feb 25;27(5):1553. doi: 10.3390/molecules27051553 (PMC8911913; doi:10.3390/molecules27051553)
Supplement: Supplementary file 1 [file molecules-27-01553-s001.zip › molecules-1538648-supplementary.pdf]

Supplementary Section

Design, Synthesis, and *In vivo* Evaluation of C1-Linked 4,5-Epoxymorphinan Haptens for Heroin Vaccines

Agnieszka Sulima,[1] Fuying Li,[1] J. Brian Morgan,[1] Phong Truong,[1,a] Joshua F. G. Antoline,[1] Therese Oertel,[2,b] Rodell C. Barrientos,[2,3,c] Oscar B. Torres,[2,3,c] Zoltan Beck,[2,3,d] Gregory H. Imler,[4] Jeffrey R. Deschamps,[4] Gary R. Matyas,[2] Arthur E. Jacobson[1,*] and Kenner C. Rice[1,*]

[1]Drug Design and Synthesis Section, Molecular Targets and Medications Discovery Branch, Intramural Research Program, National Institute on Drug Abuse and the National Institute on Alcohol Abuse and Alcoholism, National Institutes of Health, Department of Health and Human Services, 9800 Medical Center Drive, Bethesda, MD 20892-3373, USA.

[2]Laboratory of Adjuvant and Antigen Research, US Military HIV Research Program, Walter Reed Army Institute of Research, 503 Robert Grant Avenue, Silver Spring, MD 20910 USA

[3]U.S. Military HIV Research Program, Henry M. Jackson Foundation for the Advancement of Military Medicine, 6720A Rockledge Drive, Bethesda, MD 20817 USA.

[4]Center for Biomolecular Science and Engineering, Naval Research Laboratory, Washington DC 20375, United States

*Correspondence kcr: kennerr@mail.nih.gov, Tel.: 301-451-4799; aej: arthurj@mail.nih.gov, Tel.: 301-451-5028

[a] Current address: Emory Institute for Drug Development, Emory University, Atlanta, GA, 3039, United States

[b] Current address: Department of Chemistry, Georgetown University, Washington, DC, 20057, United States

[c] Current address: Merck & Co., Inc., 126 E Lincoln Avenue, Rahway, NJ 07065, United States

[d] Current address: Pfizer, Inc., 401 N Middletown Rd, Pearl River, NY 10965, United States

Table of Contents

^{1}H NMR of 1..………………………………………………………………..………4
^{13}C NMR of 1…………………………………….……………….……..……4
^{1}H NMR of 2.……………………………………………………………..………..5
^{13}C NMR of 2………………………………………..………………….….……5
^{1}H NMR of 3..………………………………………………………………..………6
^{13}C NMR of 3…………………………………….…………………..…….6
^{1}H NMR of 4..……………………………………………………….….….……7
^{13}C NMR of 4……………………………………….…………………….…….7
^{1}H NMR of 6..……………………………………………………….….….……8
^{13}C NMR of 6…………………………………….…………………..…….8
^{1}H NMR of 7..……………………………………………………….….….……9
^{13}C NMR of 7…………………………………….…………………..…….9
^{1}H NMR of 8..………………………………………………………………..………10
^{13}C NMR of 8…………………………………….………………….….……10
^{1}H NMR of 11..…………………………………………………….….……...11
^{13}C NMR of 11…………………………………….……………….……….…...11
^{1}H NMR of 12..…………………………………………………….….……….…...12
^{13}C NMR of 12…………………………………….………………….……….…...12
^{1}H NMR of 15..…………………………………………………….….……….…...13
^{13}C NMR of 15…………………………………….……………….……….…...13
^{1}H NMR of 16..………………………………………………………….….………14
^{13}C NMR of 16…………………………………….………………….….………14
^{1}H NMR of 17..…………………………………………………………...………15
^{13}C NMR of 17..……………………………………………………….………15
^{1}H NMR of 20………………………………….………………………….……..16
^{13}C NMR of 20…………………………………….…………………….………17
^{1}H NMR of 21..………………………………………………………….………18
^{13}C NMR of 21…………………………………….……………………….………19
^{1}H NMR of 22..……………………………………………………………….……….20
^{13}C NMR of 22…………………………………….……………………….………21
^{1}H NMR of 23..………………………………………………………….………22

^{13}C NMR of 23……………………………………………………………………………….……….23
^{1}H NMR of 25………………………………………………………………………….………..24
^{13}C NMR of 25……………………………………………………………………………………25
^{1}H NMR of 26..……………………………………………………………………….………..26
^{13}C NMR of 26……………………………………………………………………………………26
^{1}H NMR of 29..……………………………………………………………………….………..27
^{13}C NMR of 29……………………………………………………………………………………27
^{1}H NMR of 30..……………………………………………………………………….………..28
^{13}C NMR of 30……………………………………………………………………………………28
^{1}H NMR of 31..……………………………………………………………………….………..29
^{13}C NMR of 31……………………………………………………………………………………29
^{1}H NMR of 32..……………………………………………………………………….………..30
^{13}C NMR of 32……………………………………………………………………………………30
^{1}H NMR of 34..……………………………………………………………………….………..31
^{13}C NMR of 34……………………………………………………………………………………31
X-ray crystallographic data for compound **16** …………….……….………………………32-39
Structures of Naloxone and Naltrexone ………….……………….……….………………40

Compound **1**

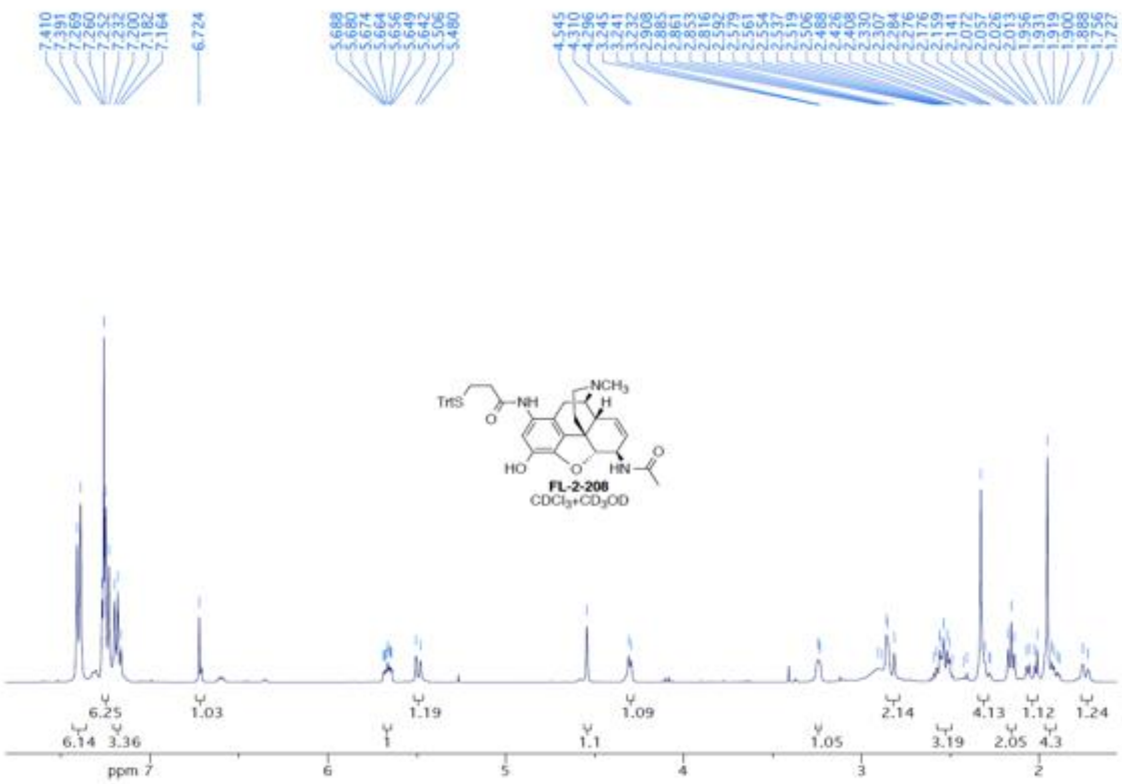

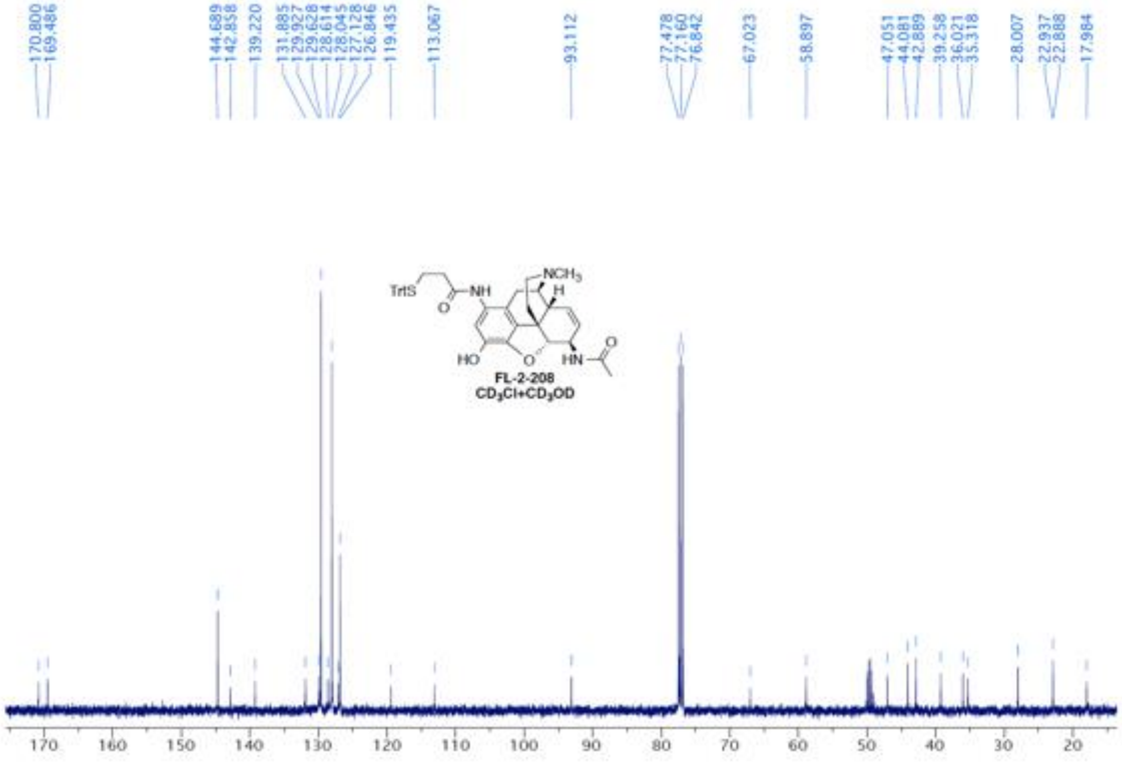

Compound **2**

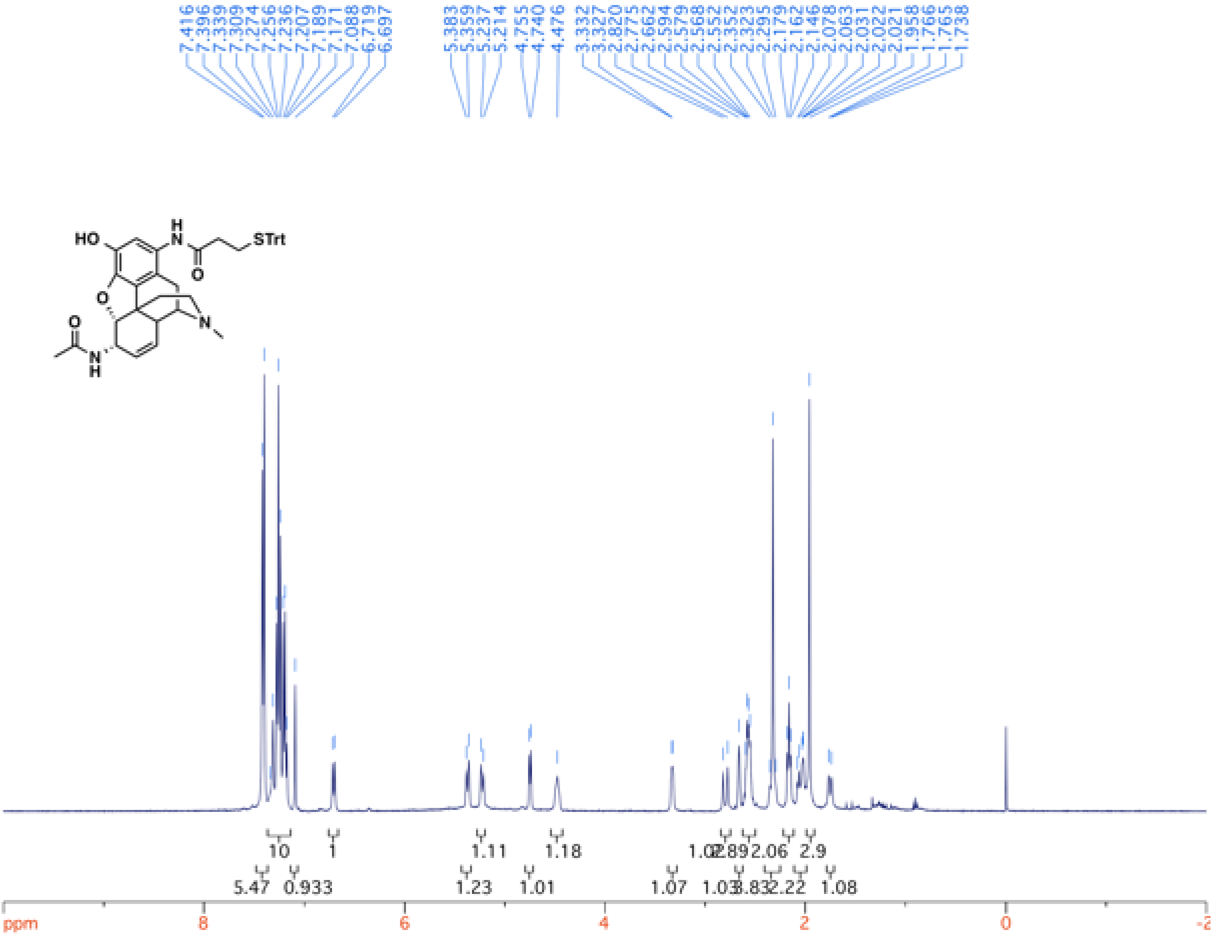
7.416
7.396
7.339
7.309
7.274
7.256
7.236
7.207
7.189
7.171
7.088
6.719
6.697
5.383
5.359
5.237
5.214
4.755
4.740
4.476
3.332
3.327
2.820
2.775
2.662
2.594
2.579
2.568
2.552
2.352
2.323
2.295
2.179
2.162
2.146
2.078
2.063
2.031
2.022
2.021
1.958
1.766
1.765
1.738
HO
H
N
STrt
O
O
N
O
N
H
10
1
1.11
1.18
1.02
2.89
2.06
2.9
5.47
0.933
1.23
1.01
1.07
1.03
3.83
2.22
1.08
ppm
8
6
4
2
0

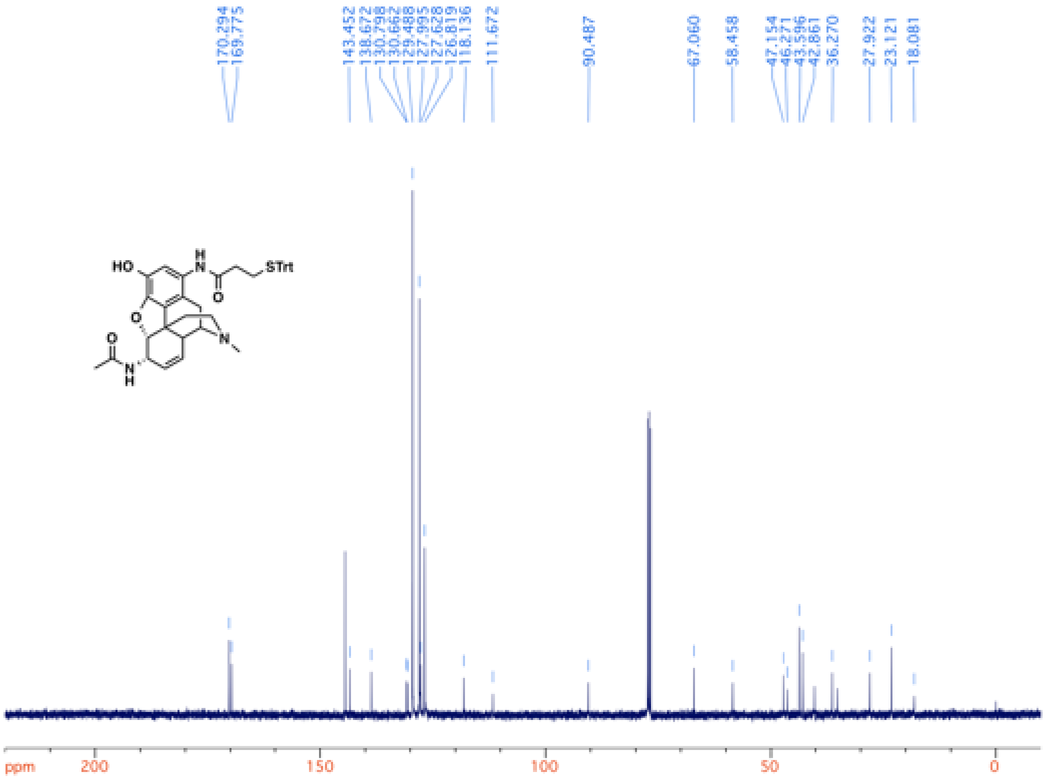
170.294
169.775
143.452
138.672
130.798
130.662
129.488
127.995
127.628
126.819
118.136
111.672
90.487
67.060
58.458
47.154
46.271
43.596
42.861
36.270
27.922
23.121
18.081
HO
H
N
STrt
O
O
N
O
N
H
ppm
200
150
100
50
0

Compound **3**

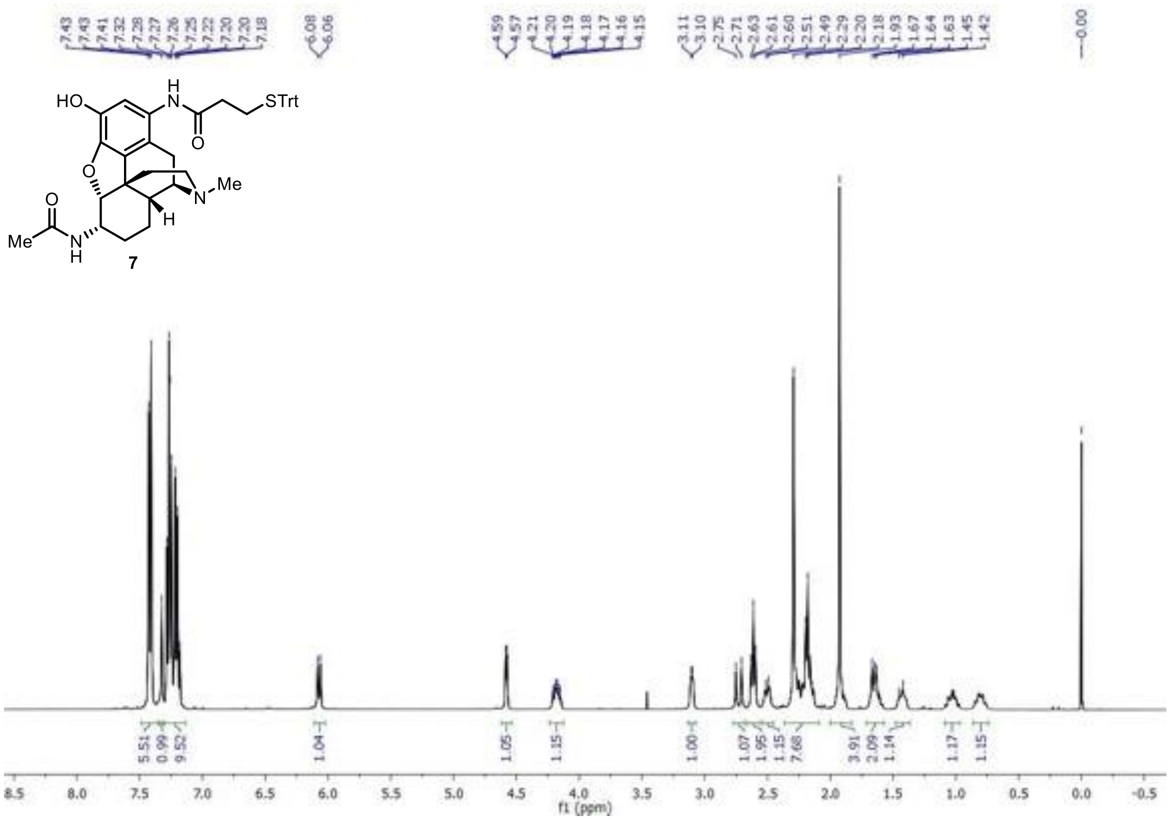

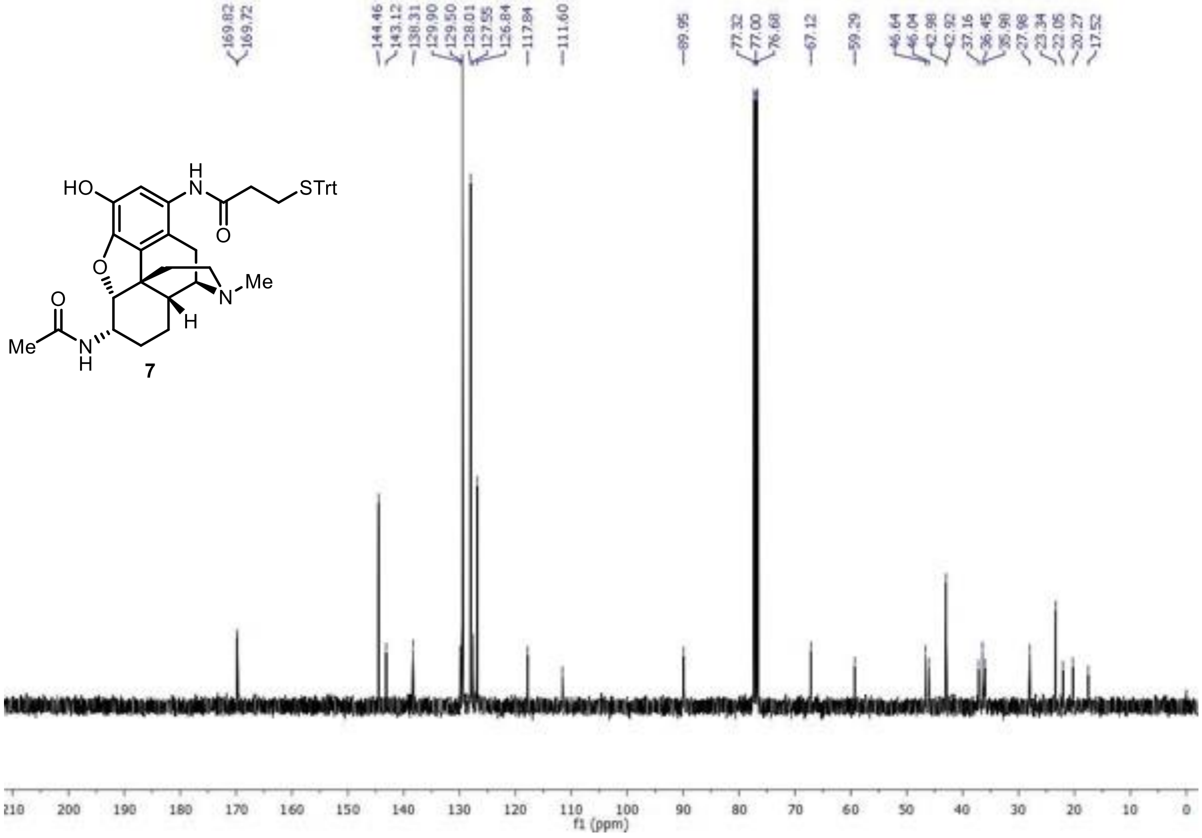

Compound **4**

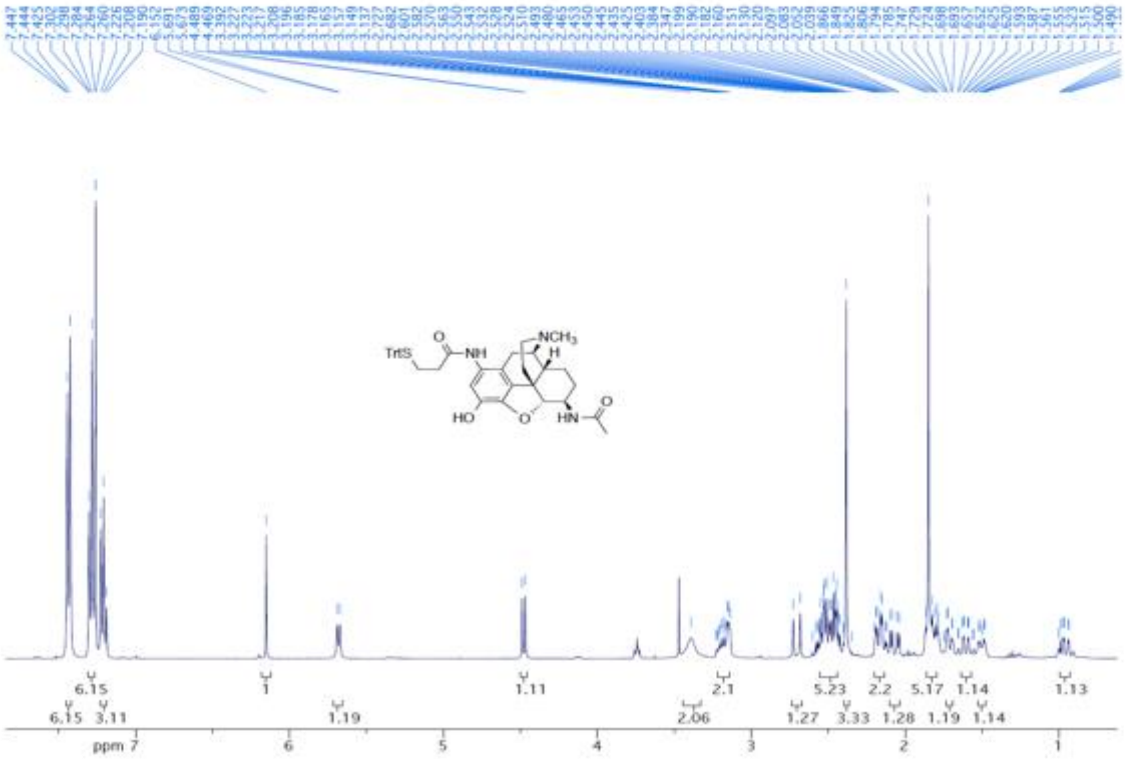
TrtS
NH
NCH3
H
HO
O
HN
O
6.15
6.15
3.11
1
1.19
1.11
2.06
2.1
1.27
5.23
3.33
2.2
1.28
5.17
1.19
1.14
1.14
1.13
ppm 7
6
5
4
3
2
1

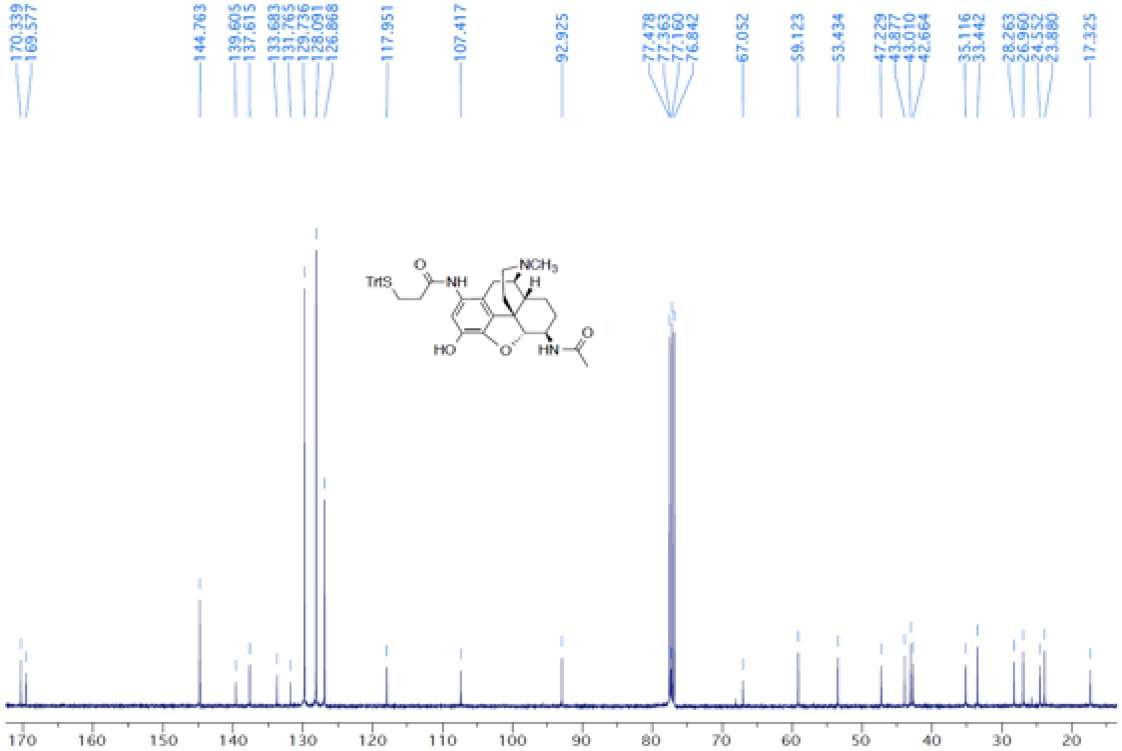
170.339
169.577
144.763
139.605
137.615
133.683
131.765
129.736
128.091
126.868
117.951
107.417
92.925
77.478
77.363
77.160
76.842
67.052
59.123
53.434
47.229
43.877
43.010
42.664
35.116
33.442
28.263
26.960
24.552
23.880
17.325
TrtS
NH
NCH3
H
HO
O
HN
O
170
160
150
140
130
120
110
100
90
80
70
60
50
40
30
20

Compound **6**

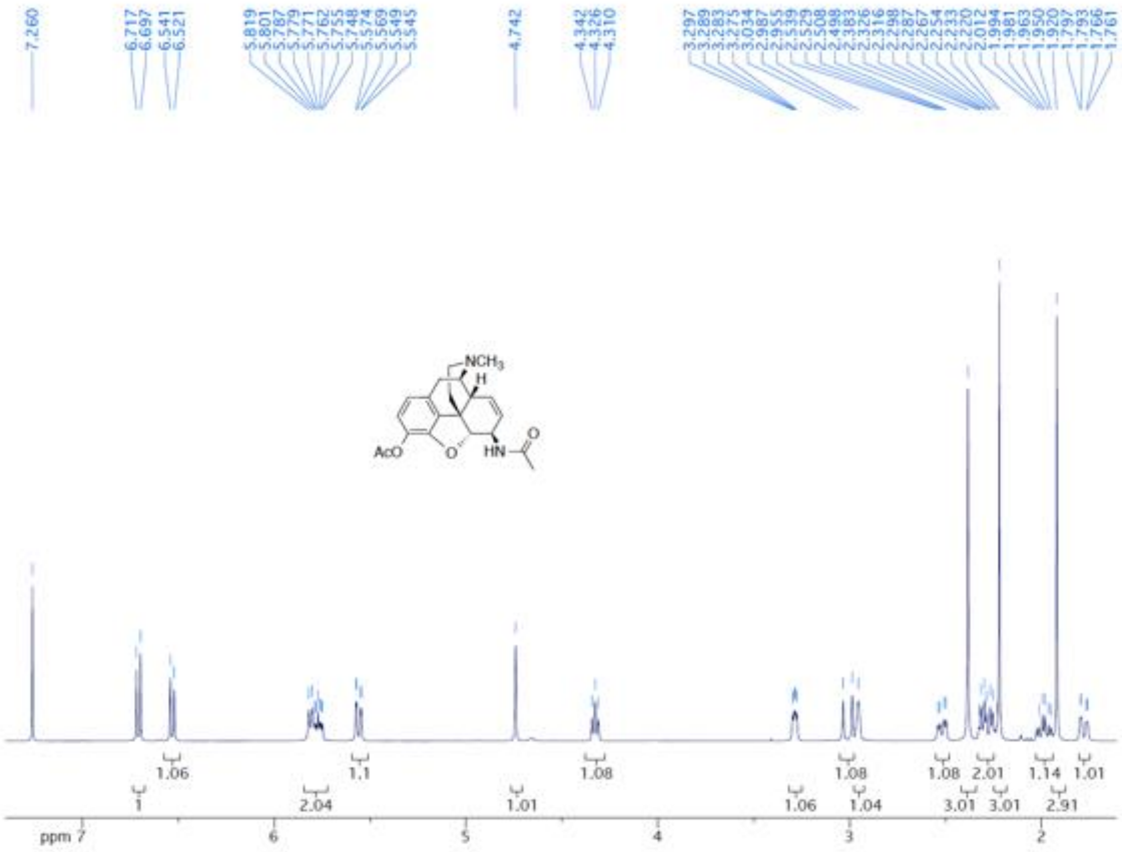

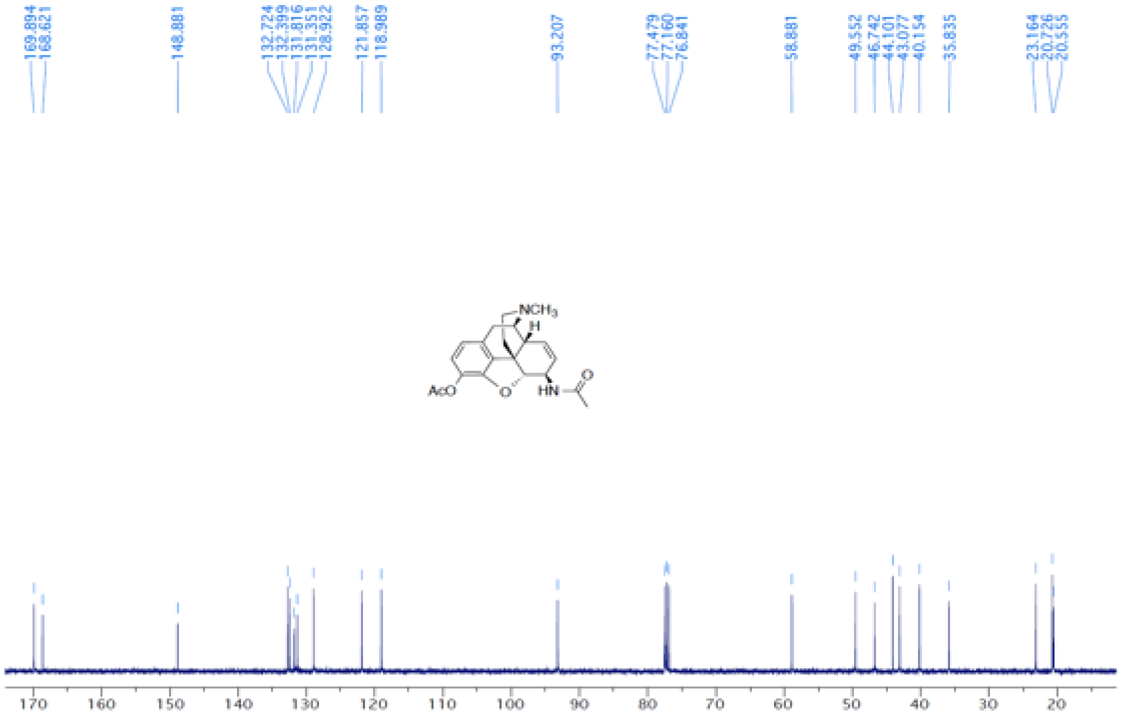

Compound **7**

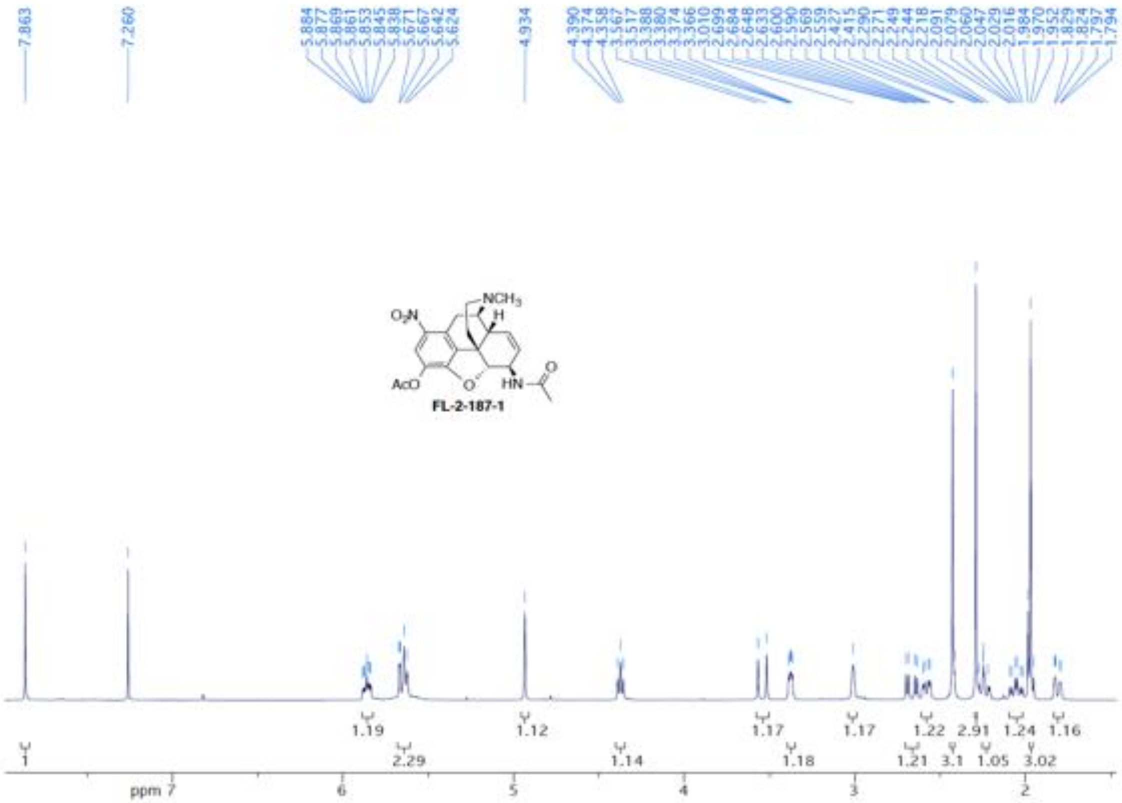
7.863
7.260
5.884
5.877
5.869
5.861
5.853
5.845
5.838
5.671
5.667
5.642
5.624
4.934
4.390
4.374
4.358
3.567
3.517
3.388
3.380
3.374
3.366
3.010
2.699
2.684
2.648
2.633
2.600
2.590
2.569
2.559
2.427
2.415
2.290
2.271
2.249
2.244
2.218
2.091
2.079
2.060
2.047
2.029
2.016
1.984
1.970
1.952
1.829
1.824
1.797
1.794
NCH3
O2N
H
AcO
O
HN
O
FL-2-187-1
1.19
2.29
1.12
1.14
1.17
1.18
1.17
1.22
1.21
2.91
3.1
1.05
1.24
3.02
1.16
1
ppm 7
6
5
4
3
2

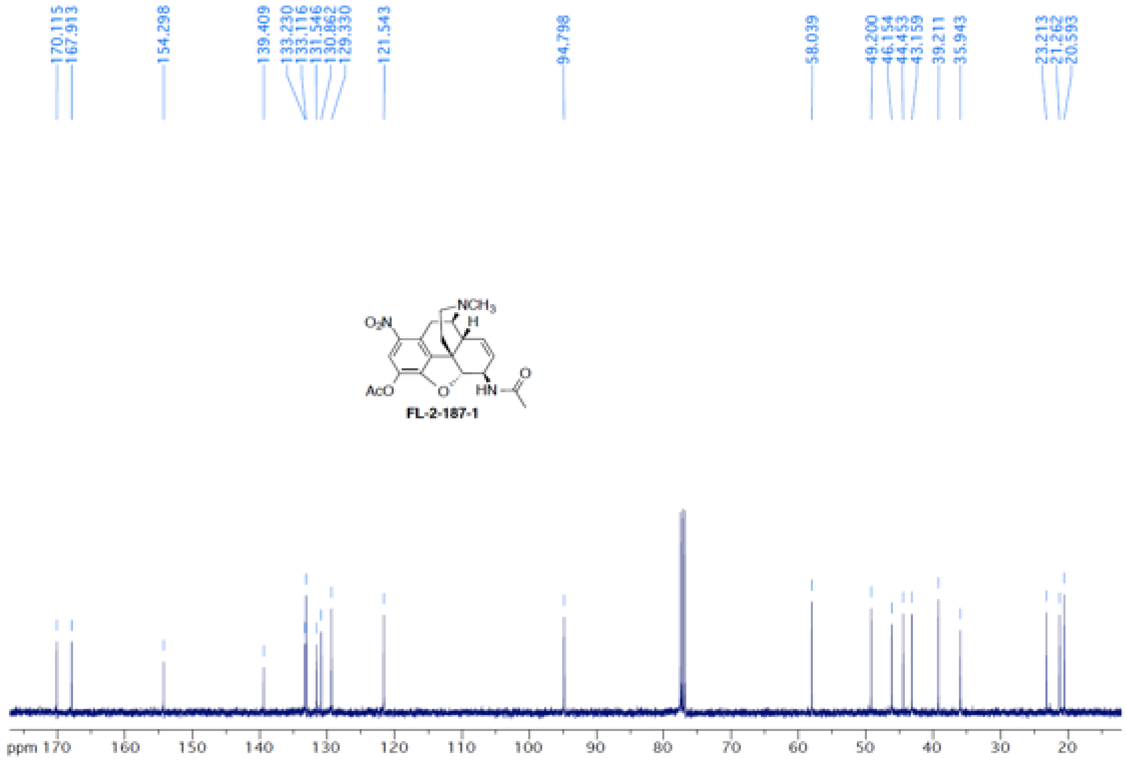
170.115
167.913
154.298
139.409
133.230
133.116
131.546
130.862
129.330
121.543
94.798
58.039
49.200
46.154
44.453
43.159
39.211
35.943
23.213
21.262
20.593
NCH3
O2N
H
AcO
O
HN
O
FL-2-187-1
ppm 170
160
150
140
130
120
110
100
90
80
70
60
50
40
30
20

Compound **8**

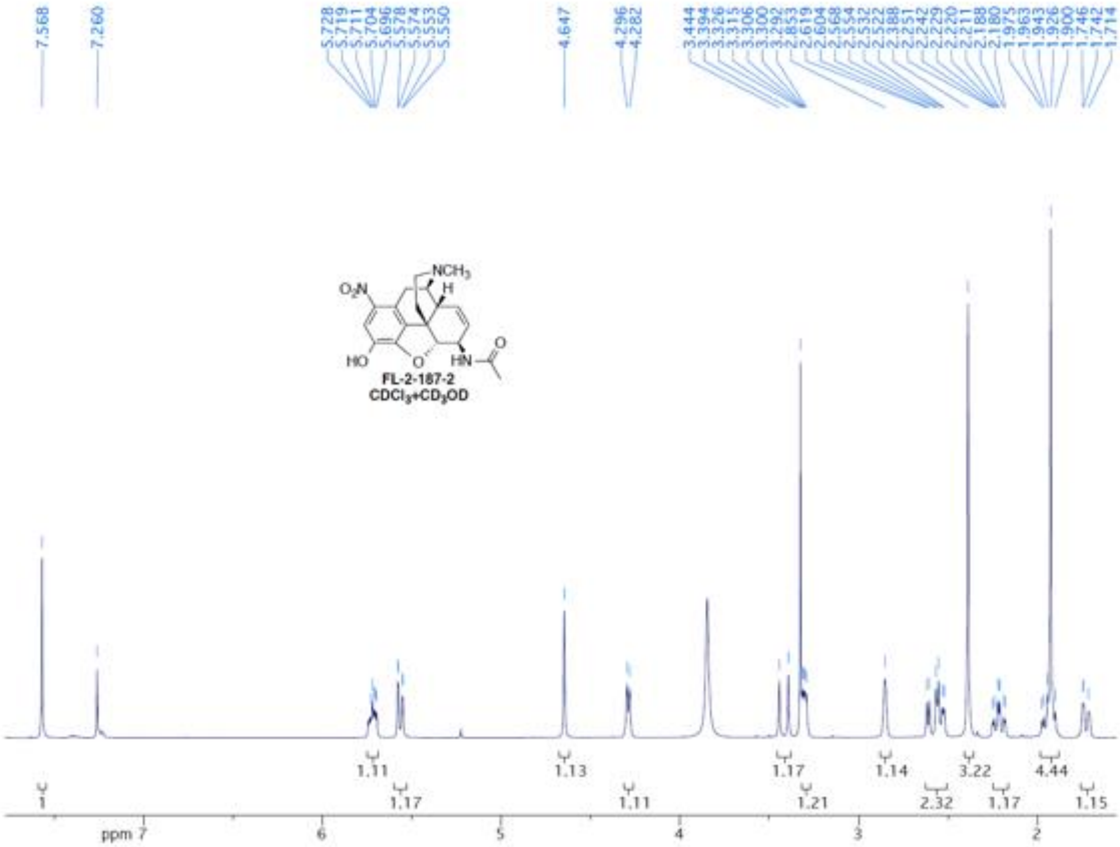

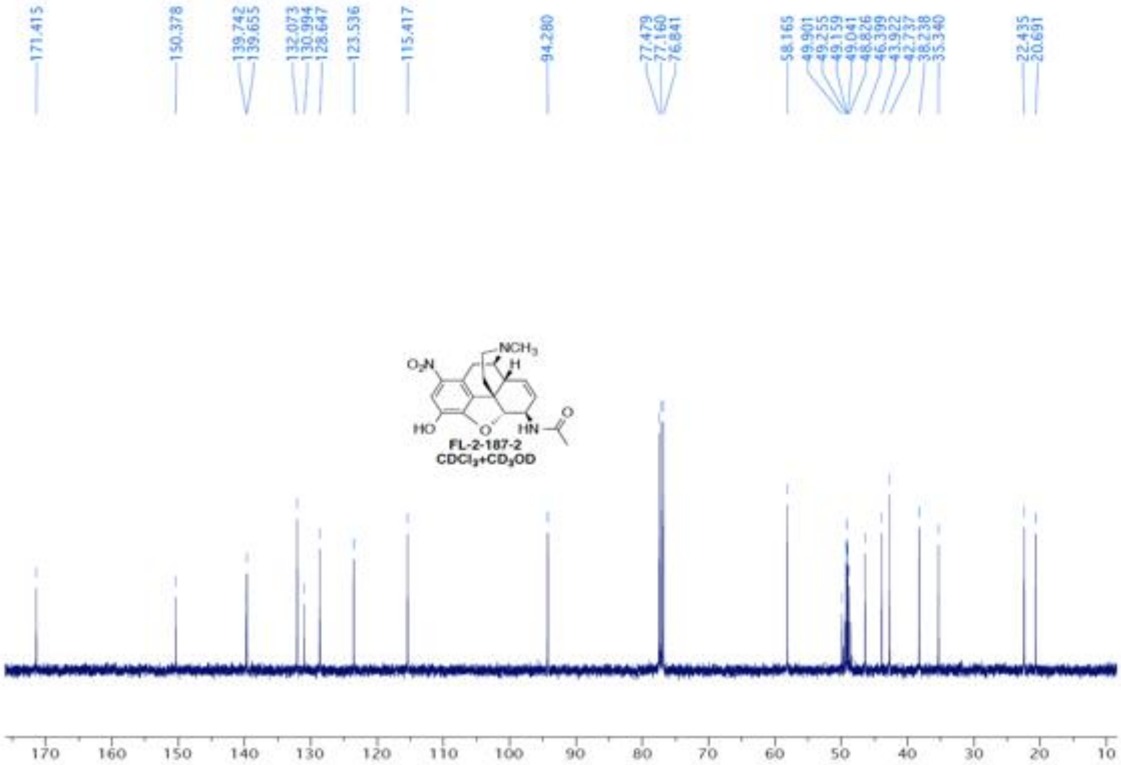

Compound **11**

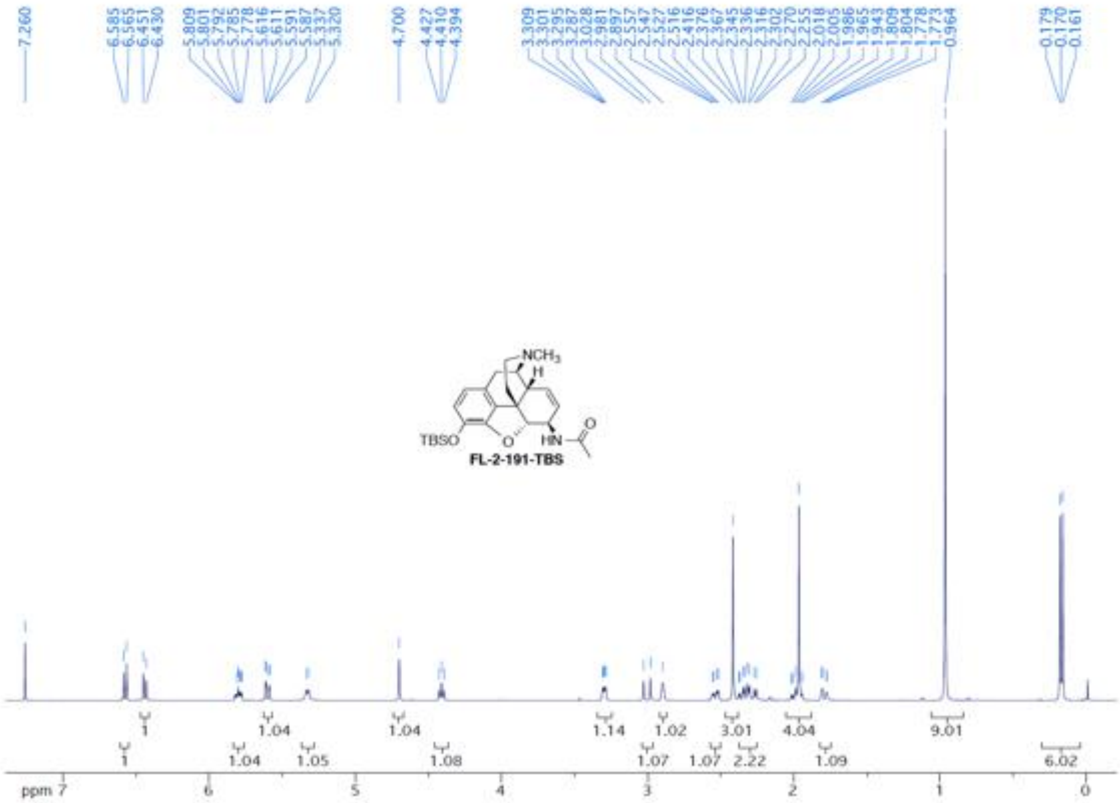
7.260
6.585
6.565
6.451
6.430
5.809
5.801
5.792
5.785
5.778
5.616
5.611
5.591
5.587
5.337
5.320
4.700
4.427
4.410
4.394
3.309
3.301
3.295
3.287
3.028
2.981
2.897
2.557
2.547
2.527
2.516
2.416
2.376
2.367
2.345
2.336
2.316
2.302
2.270
2.255
2.018
2.005
1.986
1.965
1.943
1.809
1.804
1.778
1.773
0.964
0.179
0.170
0.161
NCH3
H
TBSO
O
HN
O
FL-2-191-TBS
1
1.04
1.04
1.14
1.02
3.01
4.04
9.01
1
1.04
1.05
1.08
1.07
1.07
2.22
1.09
6.02
ppm 7
6
5
4
3
2
1
0

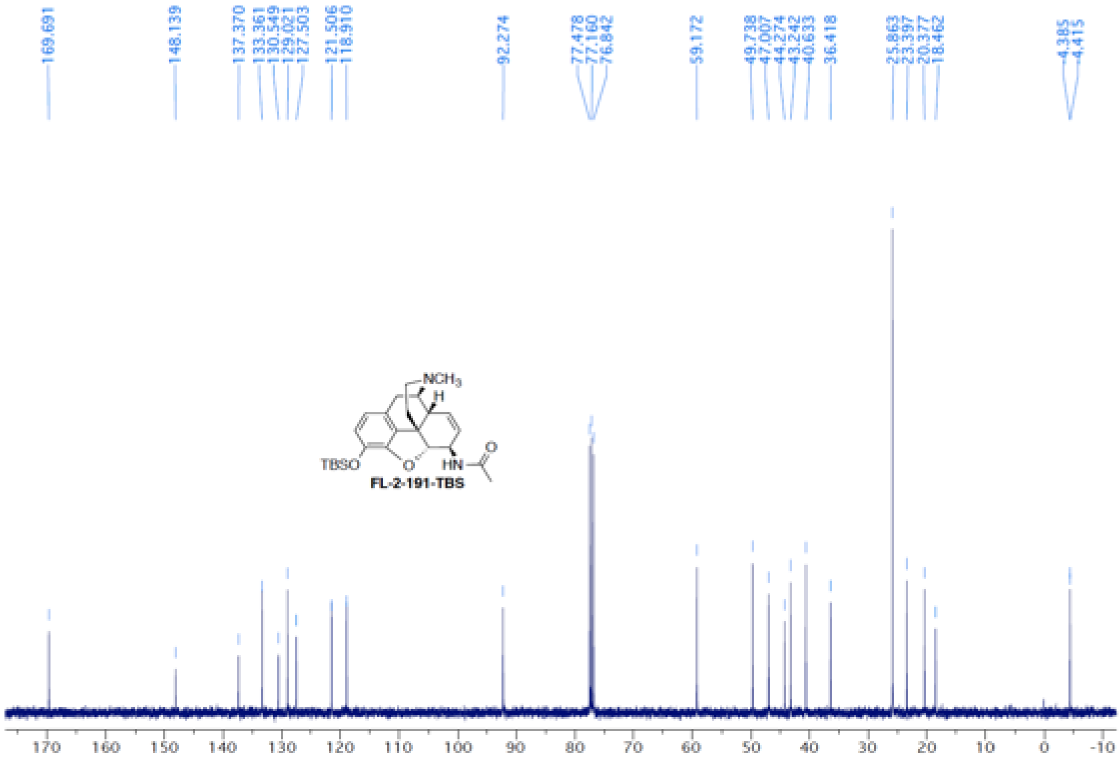
169.691
148.139
137.370
133.361
130.549
129.021
127.503
121.506
118.910
92.274
77.478
77.160
76.842
59.172
49.738
47.007
44.274
43.242
40.633
36.418
25.863
23.397
20.377
18.462
-4.385
-4.415
NCH3
H
TBSO
O
HN
O
FL-2-191-TBS
170
160
150
140
130
120
110
100
90
80
70
60
50
40
30
20
10
0
-10

Compound **12**

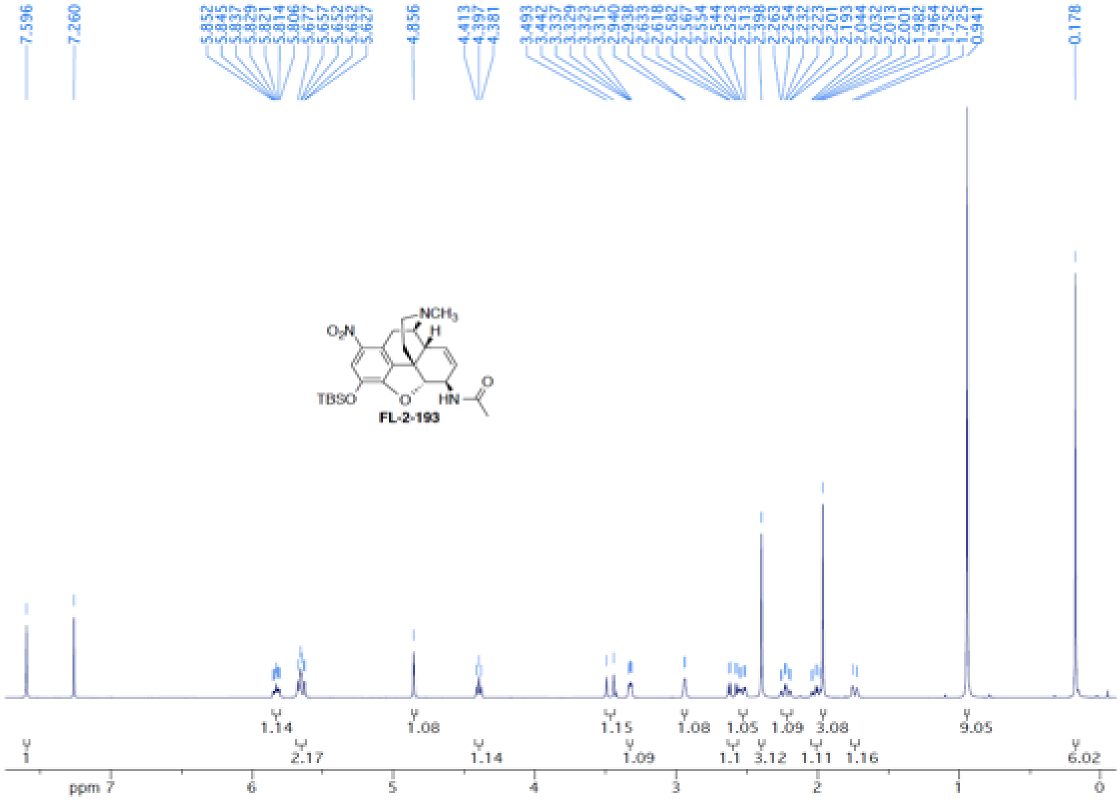
7.596
7.260
5.852
5.845
5.837
5.829
5.821
5.814
5.806
5.677
5.657
5.652
5.632
5.627
4.856
4.413
4.397
4.381
3.493
3.442
3.337
3.329
3.323
3.315
2.940
2.938
2.633
2.618
2.582
2.567
2.554
2.544
2.523
2.513
2.398
2.263
2.254
2.232
2.223
2.201
2.193
2.044
2.032
2.013
2.001
1.982
1.964
1.752
1.725
0.941
0.178
O2N
NCH3
H
TBSO
O
HN
O
FL-2-193
1.14
1.08
1.15
1.08
1.05
1.09
3.08
9.05
1
2.17
1.14
1.09
1.1
3.12
1.11
1.16
6.02
ppm 7
6
5
4
3
2
1
0

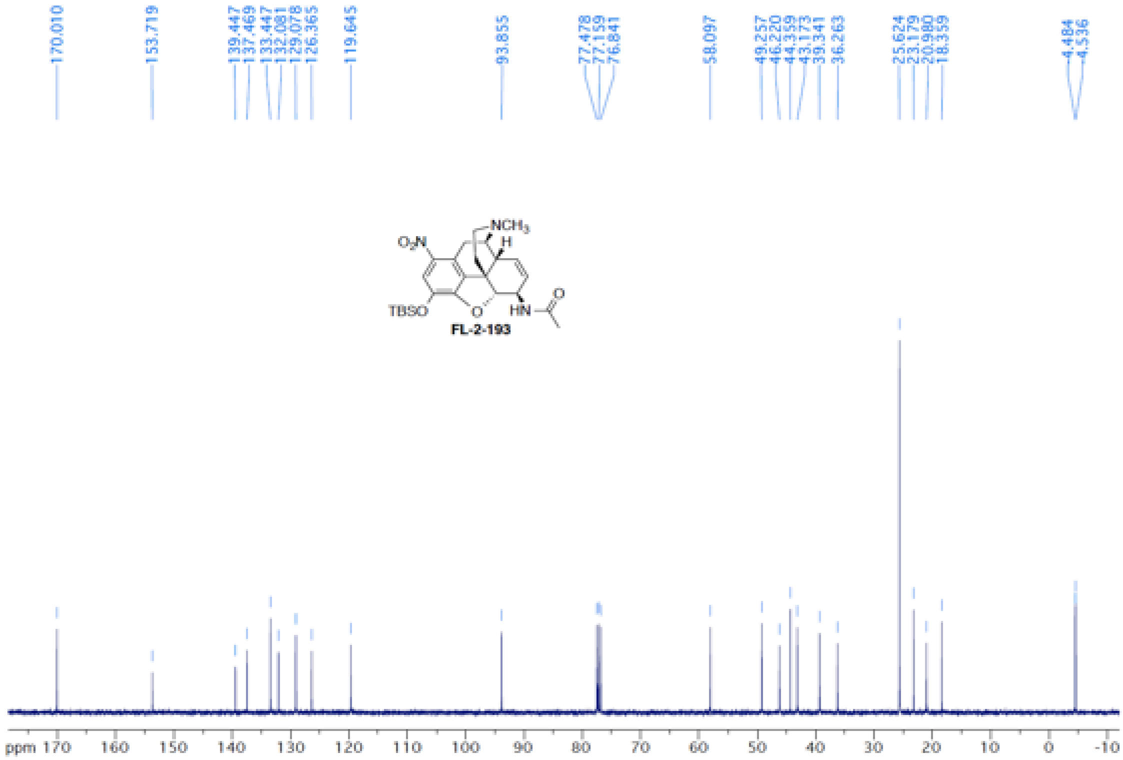
170.010
153.719
139.447
137.469
133.447
132.081
129.078
126.365
119.645
93.855
77.478
77.159
76.841
58.097
49.257
46.220
44.359
43.173
39.341
36.263
25.624
23.179
20.980
18.359
-4.484
-4.536
O2N
NCH3
H
TBSO
O
HN
O
FL-2-193
ppm 170
160
150
140
130
120
110
100
90
80
70
60
50
40
30
20
10
0
-10

Compound **15**

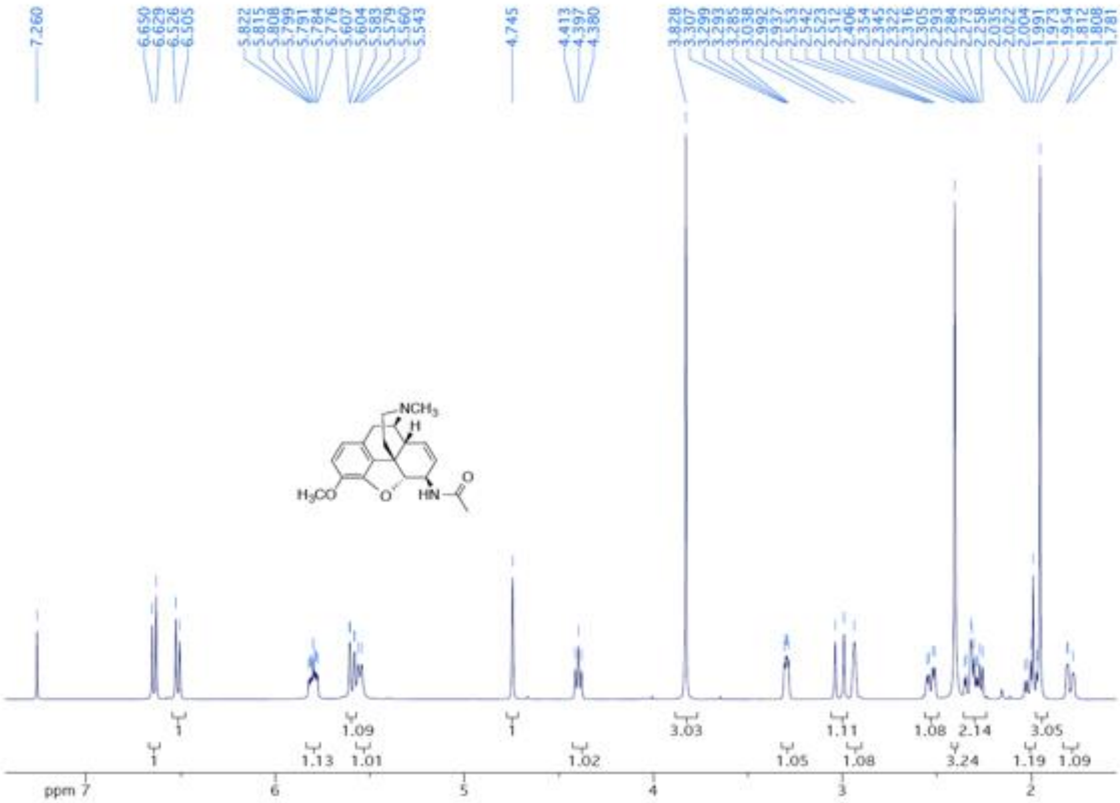

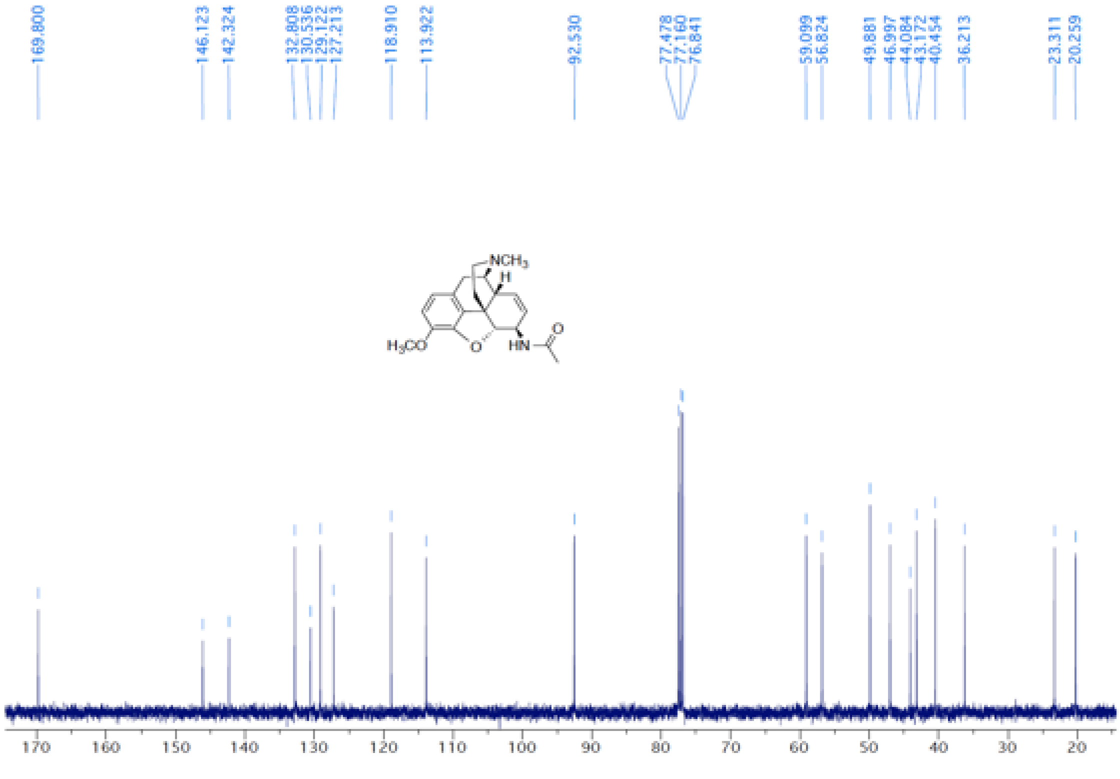

Compound **16**

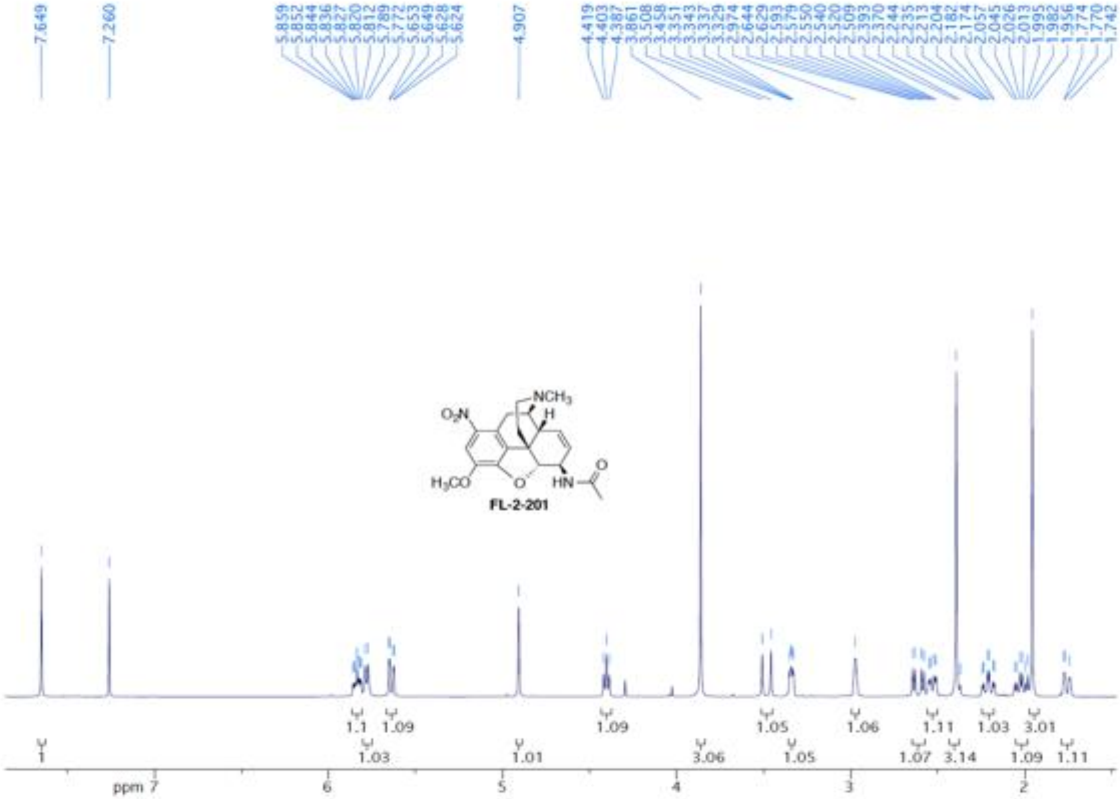

7.649
7.260
5.859 5.852 5.844 5.836 5.827 5.820 5.812 5.789 5.772 5.653 5.649 5.628 5.624
4.907
4.419 4.403 4.387 3.861 3.508 3.458 3.351 3.343 3.337 3.329 2.974 2.644 2.629 2.593 2.579 2.550 2.540 2.520 2.509 2.393 2.370 2.244 2.235 2.213 2.204 2.182 2.174 2.057 2.045 2.026 2.013 1.995 1.982 1.956 1.774 1.770 1.742
NCH3
H
O2N
H3CO
O
HN
O
FL-2-201
1 1.1 1.03 1.09 1.01 1.09 3.06 1.05 1.05 1.06 1.07 1.11 3.14 1.03 1.09 3.01 1.11
ppm 7 6 5 4 3 2

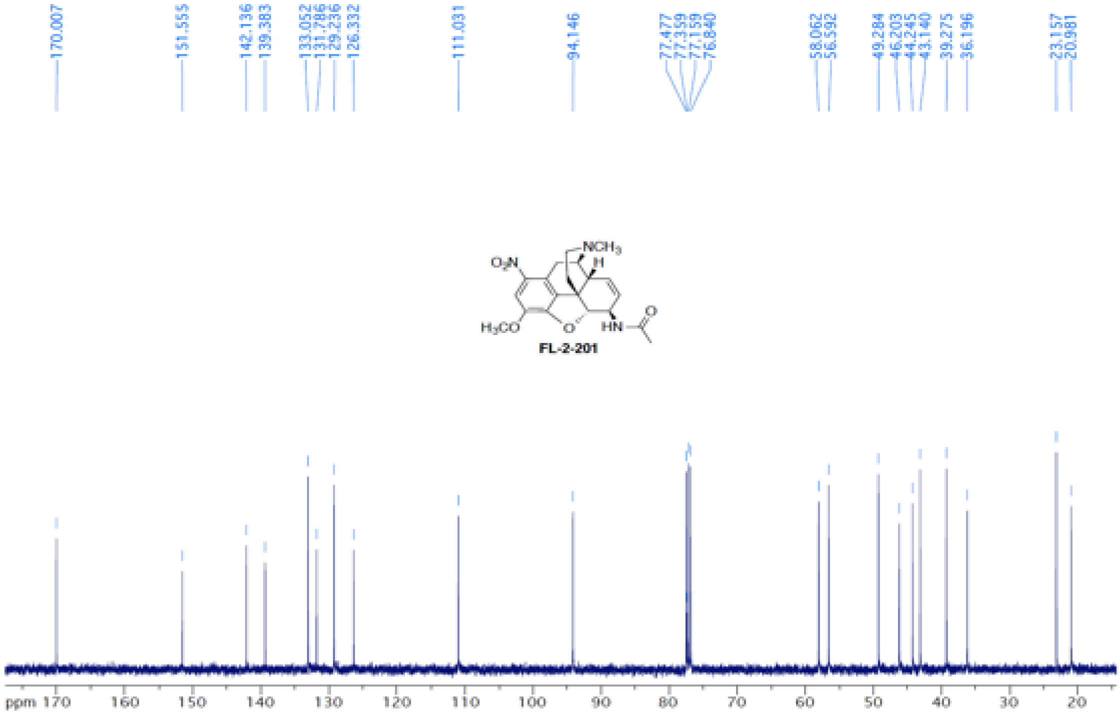

170.007
151.555
142.136 139.383
133.052 131.786 129.236 126.332
111.031
94.146
77.477 77.359 77.159 76.840
58.062 56.592
49.284 46.203 44.245 43.140 39.275 36.196
23.157 20.981
NCH3
H
O2N
H3CO
O
HN
O
FL-2-201
ppm 170 160 150 140 130 120 110 100 90 80 70 60 50 40 30 20

Compound **17**

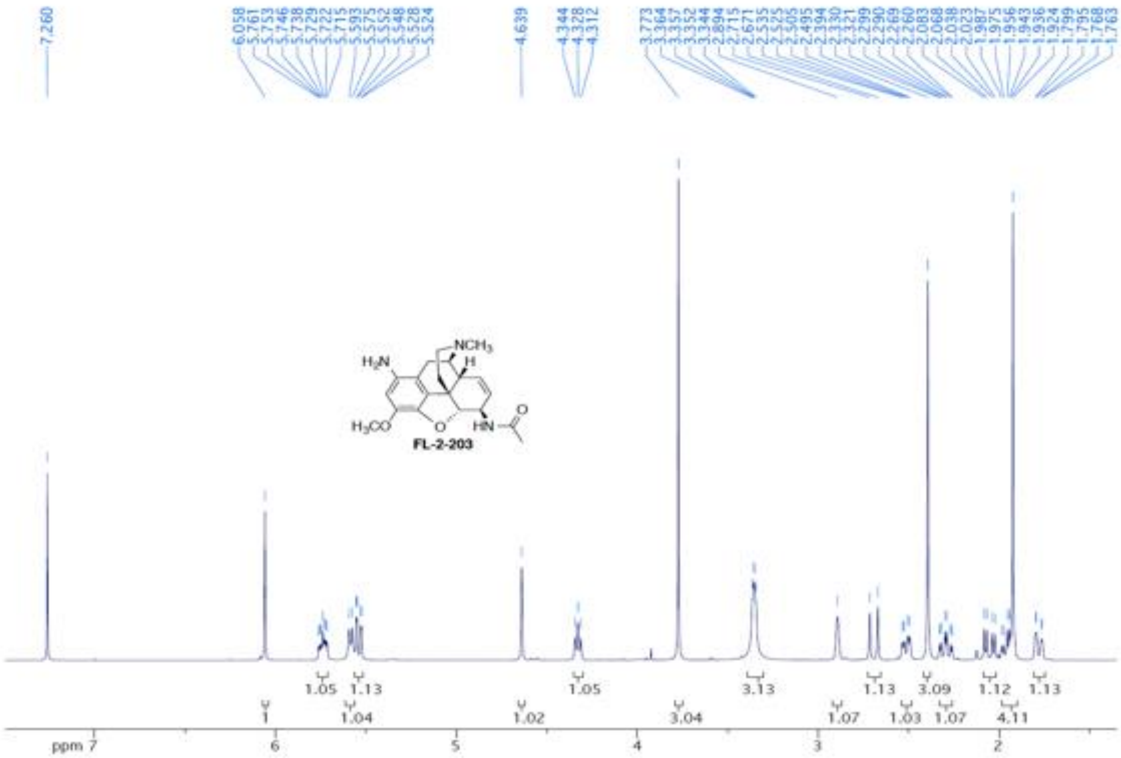
7.260
6.058
5.761
5.753
5.746
5.738
5.729
5.722
5.715
5.593
5.575
5.552
5.548
5.528
5.524
4.639
4.344
4.328
4.312
3.773
3.364
3.357
3.352
3.344
2.894
2.715
2.671
2.535
2.525
2.505
2.495
2.394
2.330
2.321
2.299
2.290
2.269
2.260
2.083
2.068
2.038
2.023
1.987
1.975
1.956
1.943
1.936
1.924
1.799
1.795
1.768
1.763
H2N
NCH3
H
H3CO
O
HN
O
FL-2-203
1.05
1.13
1.04
1
1.02
1.05
3.04
3.13
1.07
1.13
1.03
3.09
1.07
1.12
4.11
1.13
ppm 7
6
5
4
3
2

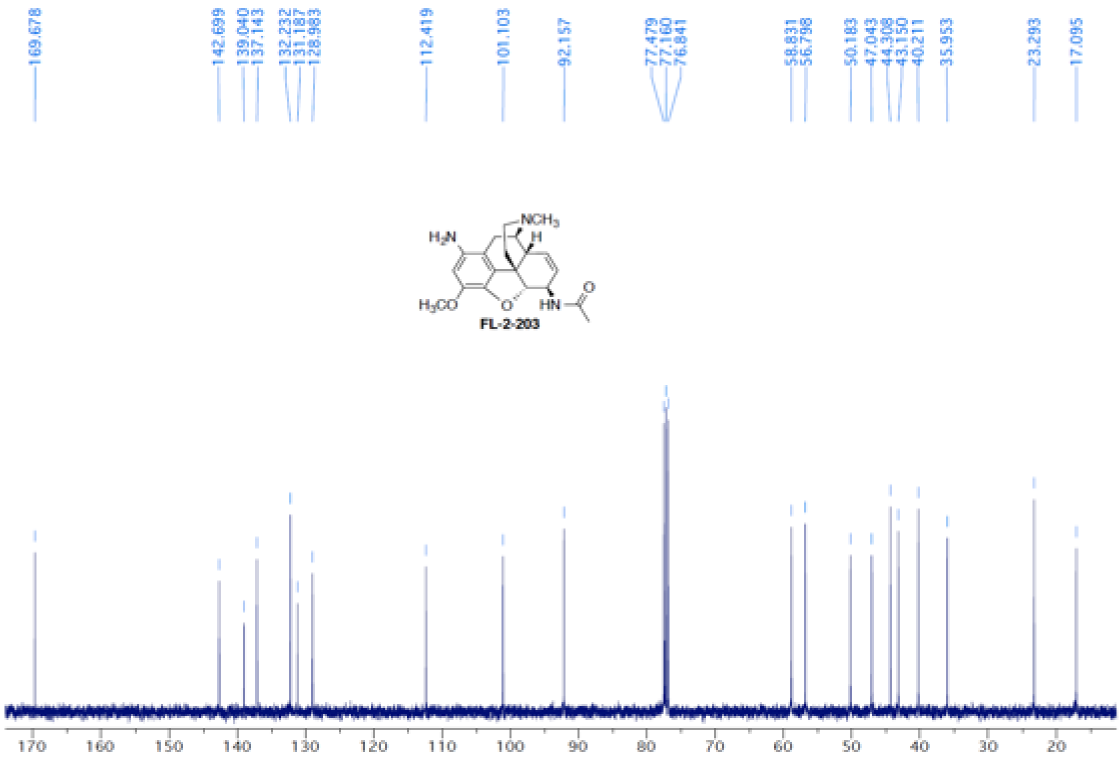
169.678
142.699
139.040
137.143
132.232
131.187
128.983
112.419
101.103
92.157
77.479
77.160
76.841
58.831
56.798
50.183
47.043
44.308
43.150
40.211
35.953
23.293
17.095
H2N
NCH3
H
H3CO
O
HN
O
FL-2-203
170
160
150
140
130
120
110
100
90
80
70
60
50
40
30
20

Compound **20**

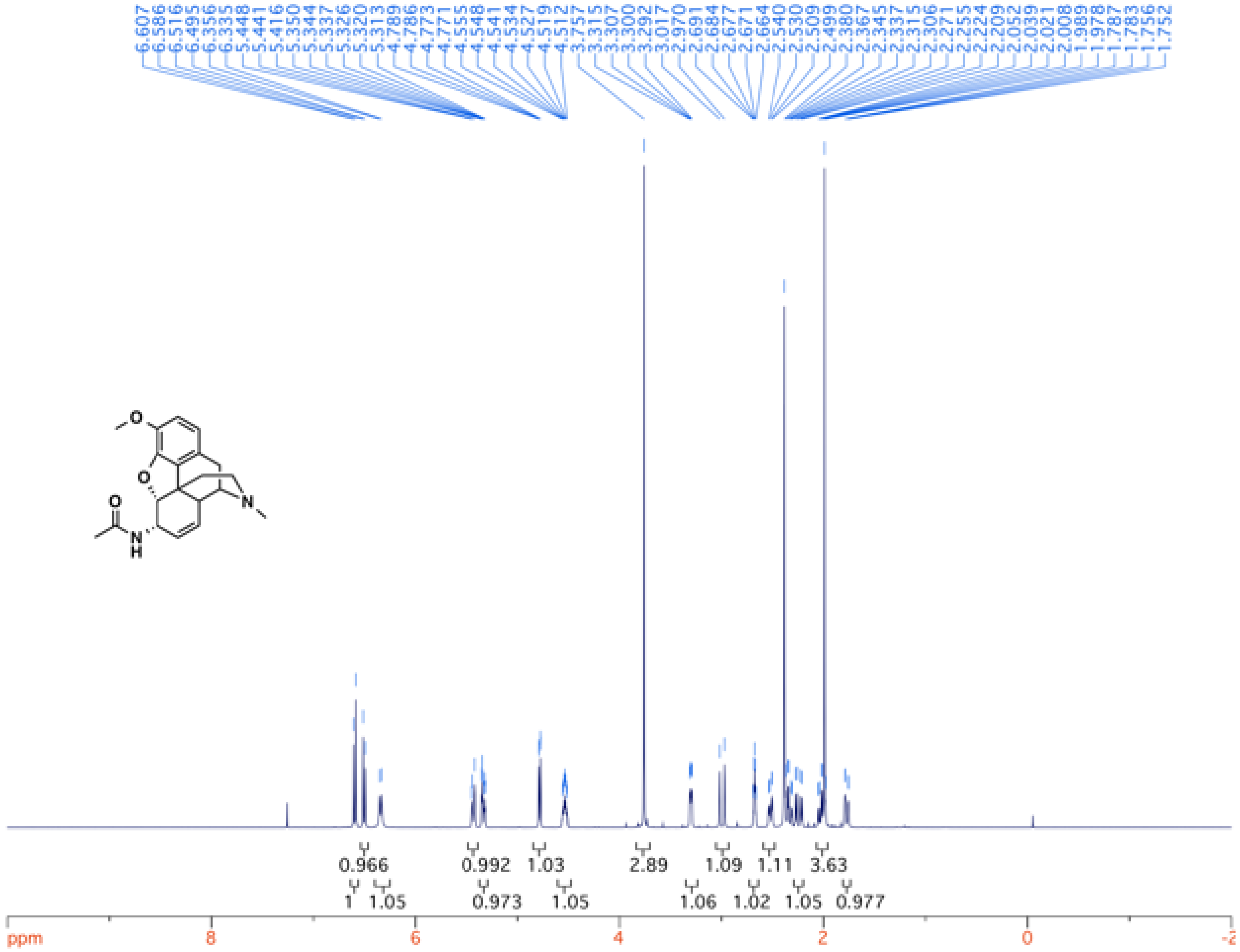
6.607
6.586
6.516
6.495
6.356
6.335
5.448
5.441
5.416
5.350
5.344
5.337
5.326
5.320
5.313
4.789
4.786
4.773
4.771
4.555
4.548
4.541
4.534
4.527
4.519
4.512
3.757
3.315
3.307
3.300
3.292
3.017
2.970
2.691
2.684
2.677
2.671
2.664
2.540
2.530
2.509
2.499
2.380
2.367
2.345
2.337
2.315
2.306
2.271
2.255
2.224
2.209
2.052
2.039
2.021
2.008
1.989
1.978
1.787
1.783
1.756
1.752
O
O
O
N
N
H
0.966
0.992
1.03
2.89
1.09
1.11
3.63
1
1.05
0.973
1.05
1.06
1.02
1.05
0.977
ppm
8
6
4
2
0

Compound **20**

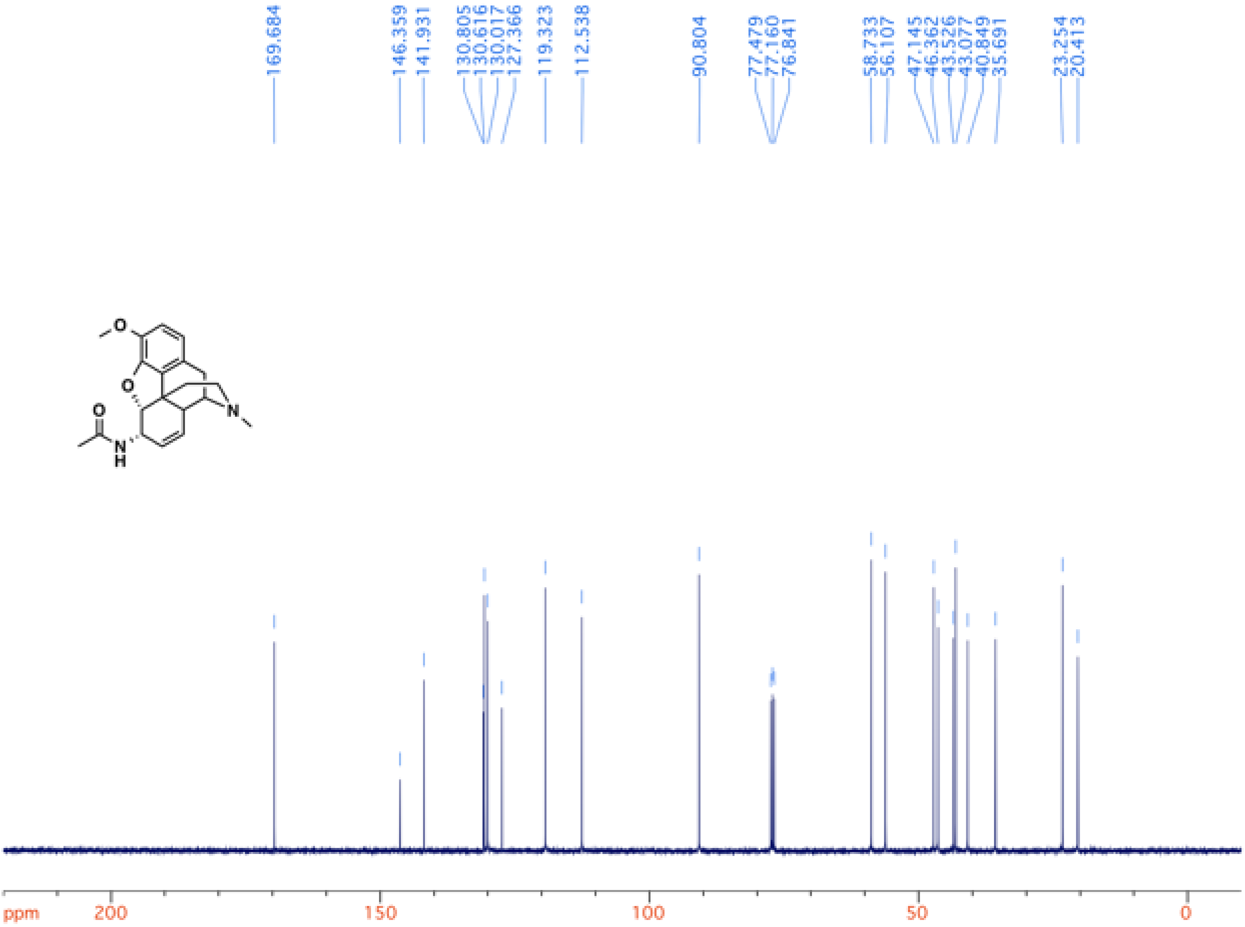
169.684
146.359
141.931
130.805
130.616
130.017
127.366
119.323
112.538
90.804
77.479
77.160
76.841
58.733
56.107
47.145
46.362
43.526
43.077
40.849
35.691
23.254
20.413
ppm
200
150
100
50
0

Compound **21**

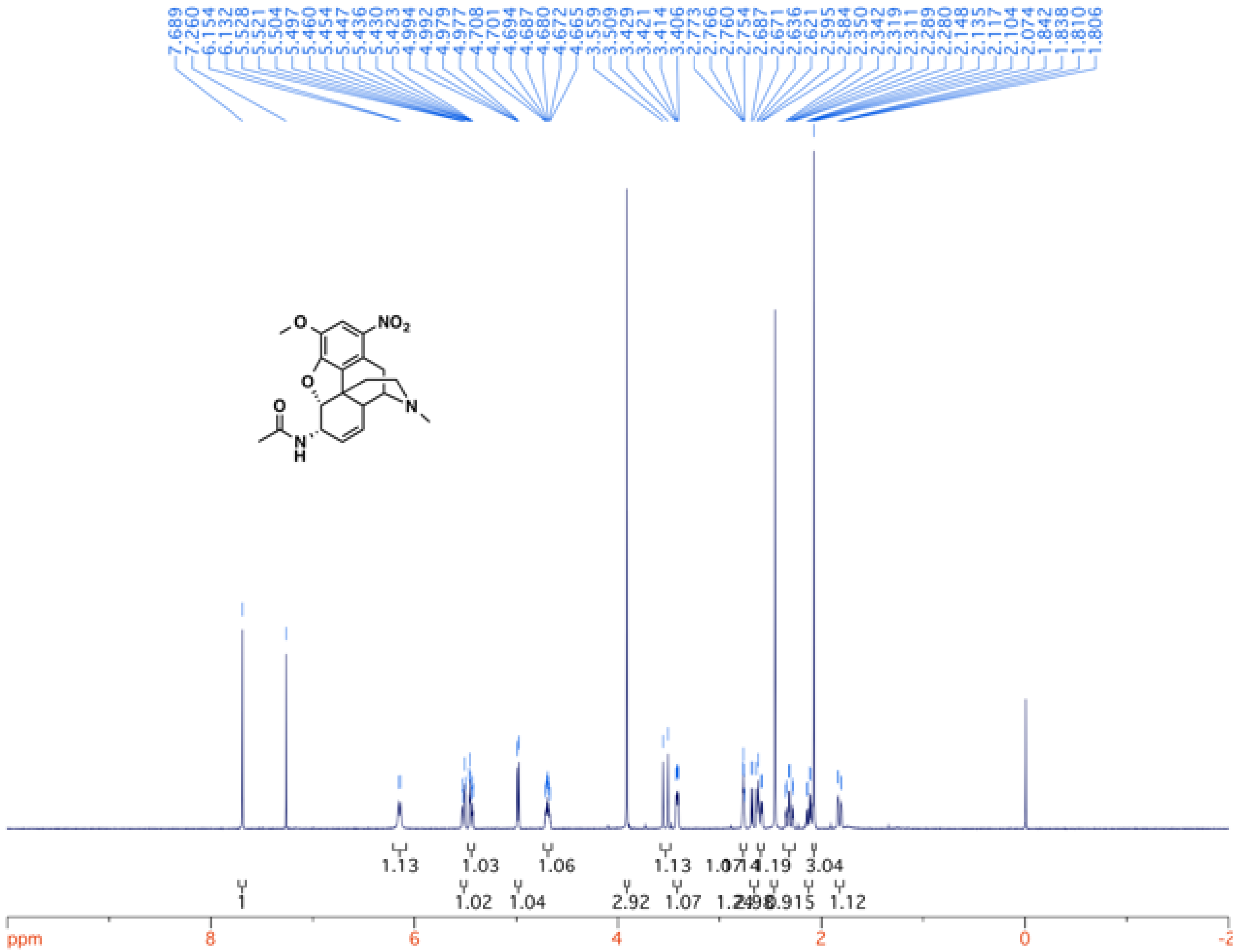

Compound **21**

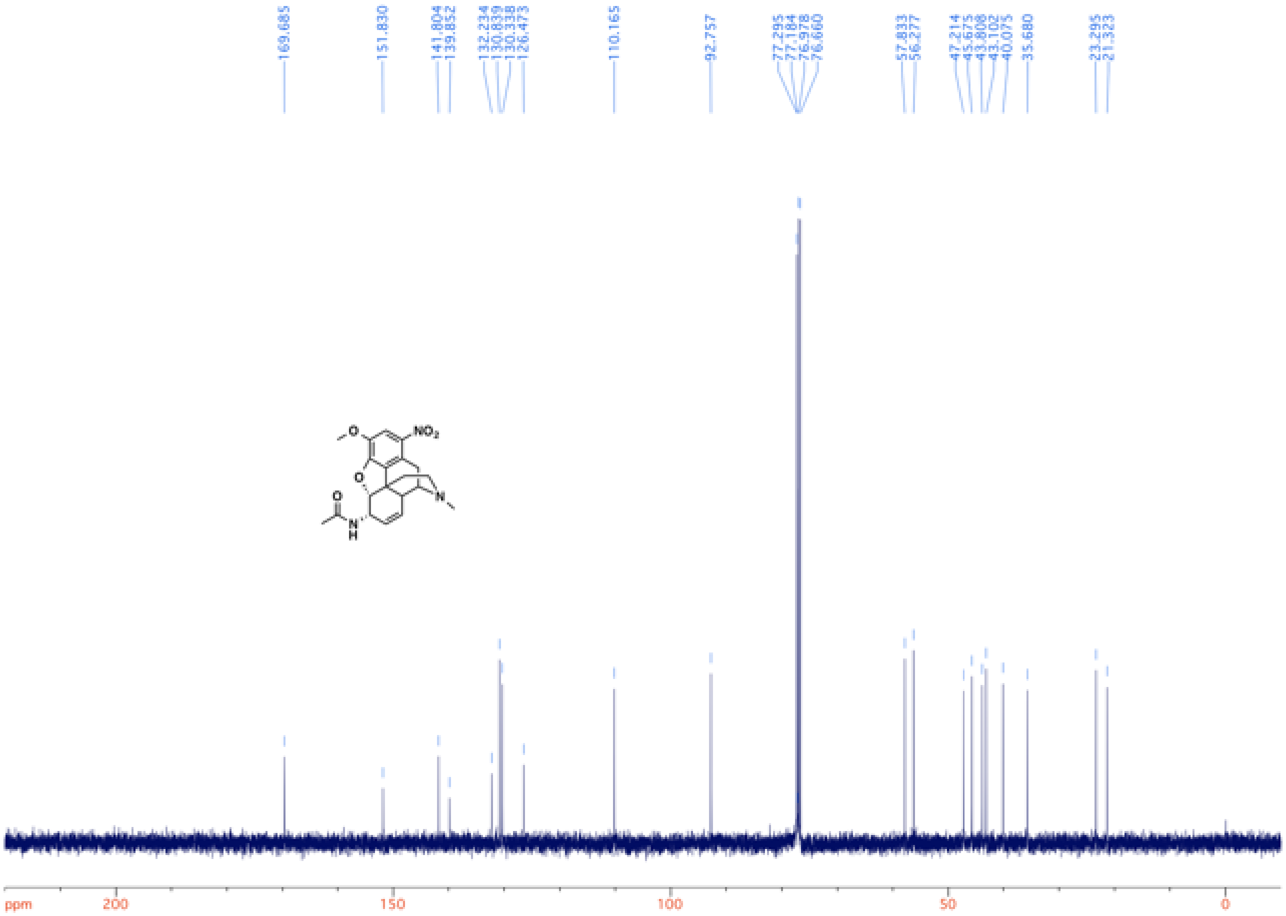
169.685
151.830
141.804
139.852
132.234
130.839
130.338
126.473
110.165
92.757
77.295
77.184
76.978
76.660
57.833
56.277
47.214
45.675
43.808
43.102
40.075
35.680
23.295
21.323
NO2
O
N
H
ppm
200
150
100
50
0

Compound **22**

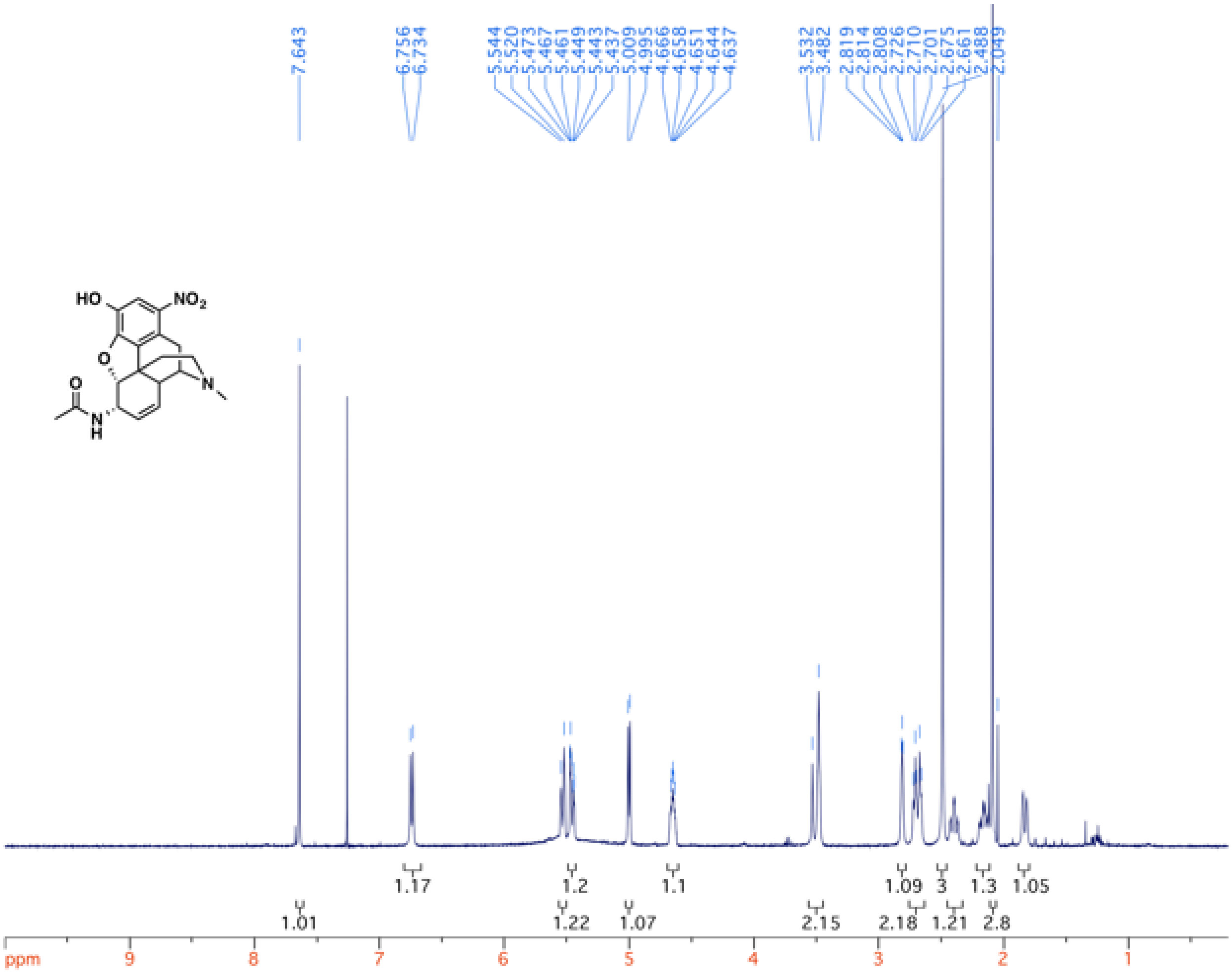

Compound **22**

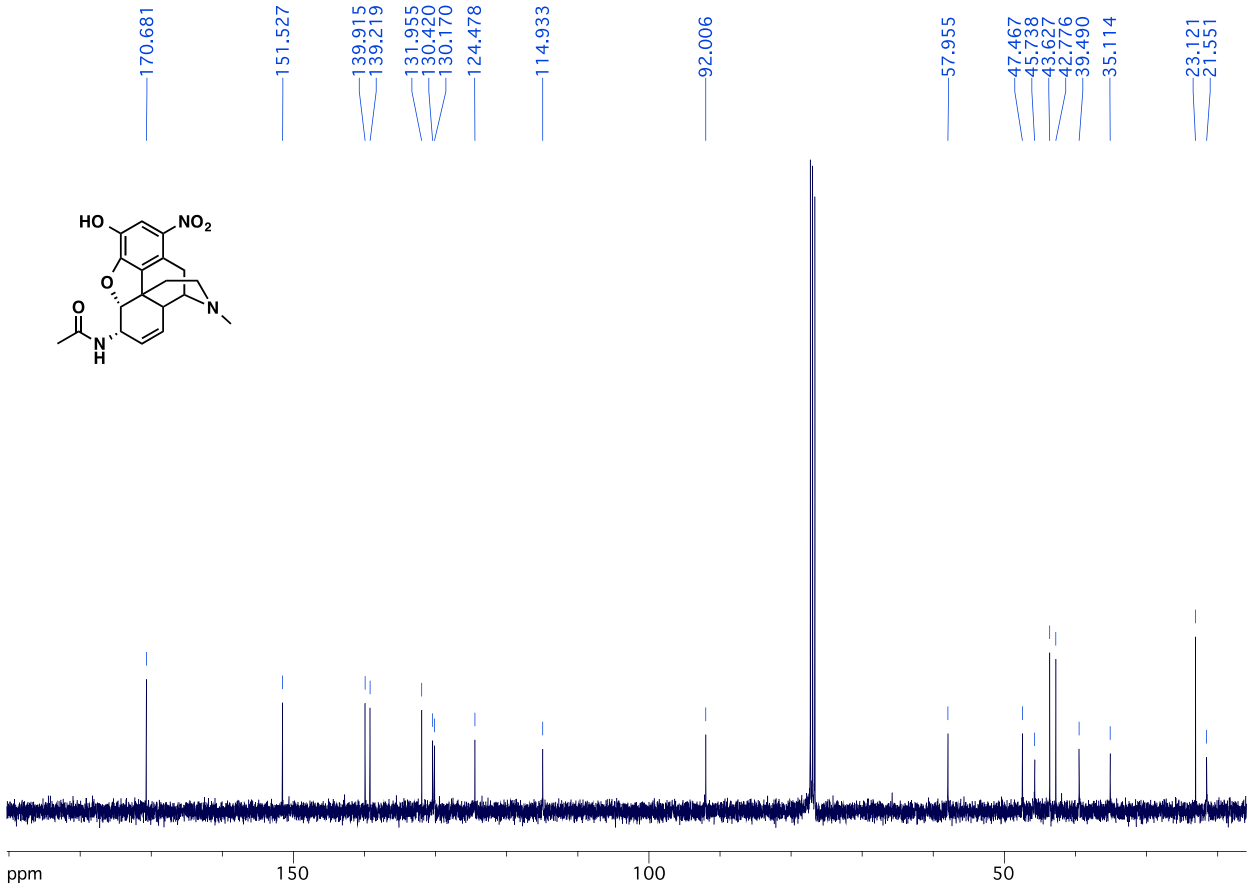

170.681
151.527
139.915
139.219
131.955
130.420
130.170
124.478
114.933
92.006
57.955
47.467
45.738
43.627
42.776
39.490
35.114
23.121
21.551
HO
NO2
O
N
O
N
H
ppm
150
100
50

Compound **23**

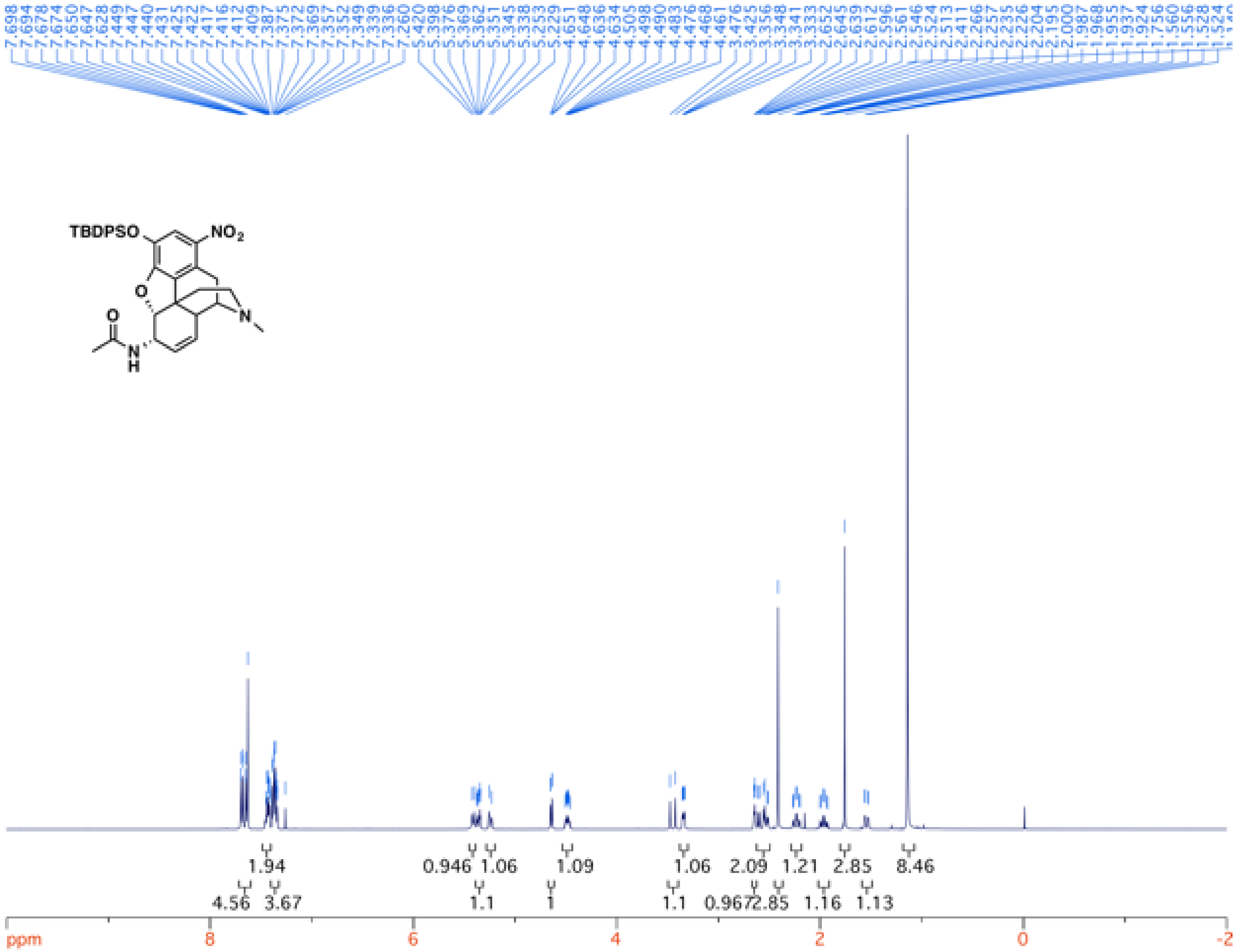
TBDPSO
NO_2
O
N
O
N
H
1.94
0.946
1.06
1.09
1.06
2.09
1.21
2.85
8.46
4.56
3.67
1.1
1
1.1
0.967
2.85
1.16
1.13
ppm
8
6
4
2
0
-2

Compound **23**

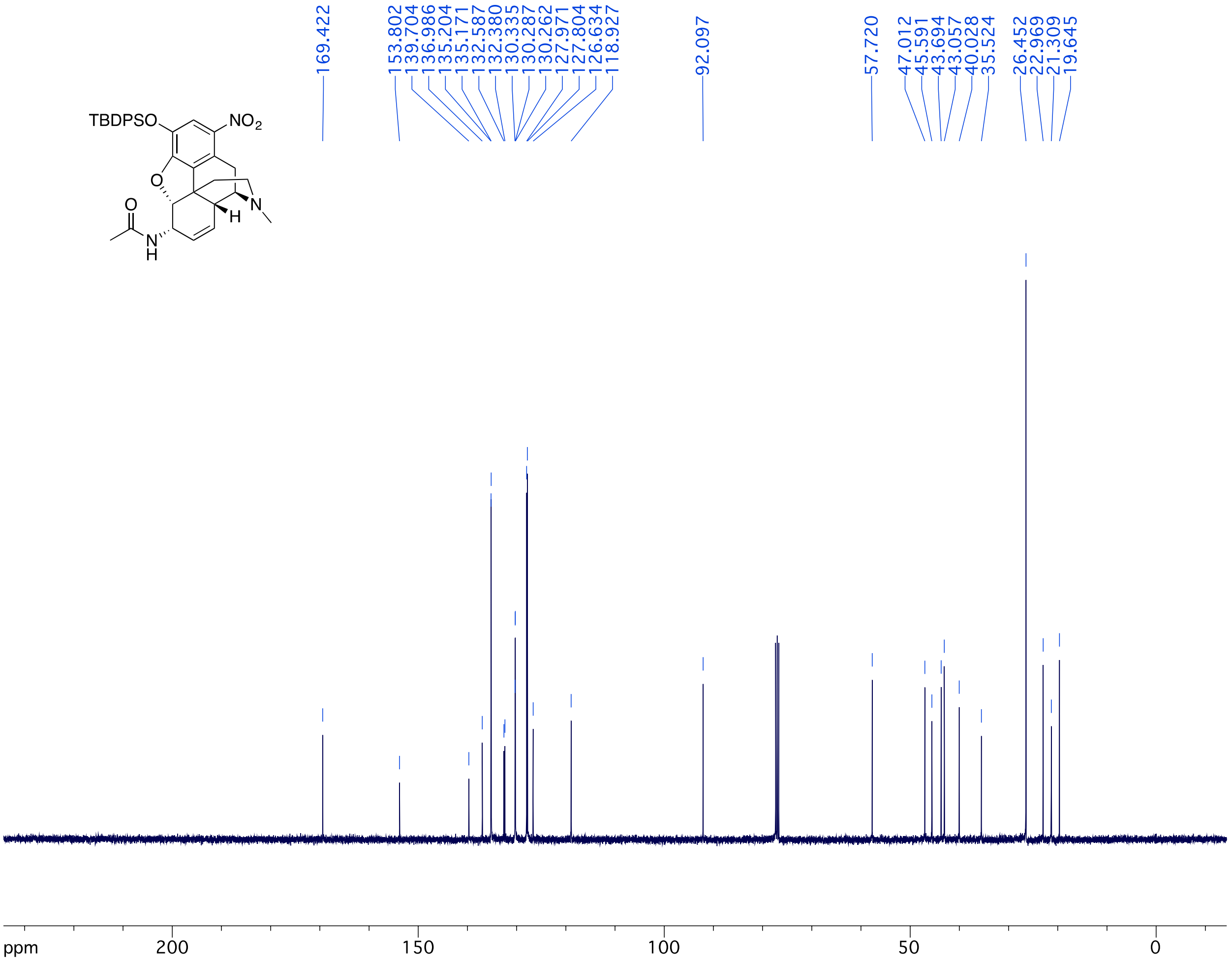
TBDPSO
NO2
O
N
H
169.422
153.802
139.704
136.986
135.204
135.171
132.587
132.380
130.335
130.287
130.262
127.971
127.804
126.634
118.927
92.097
57.720
47.012
45.591
43.694
43.057
40.028
35.524
26.452
22.969
21.309
19.645
ppm
200
150
100
50
0

Compound **25**

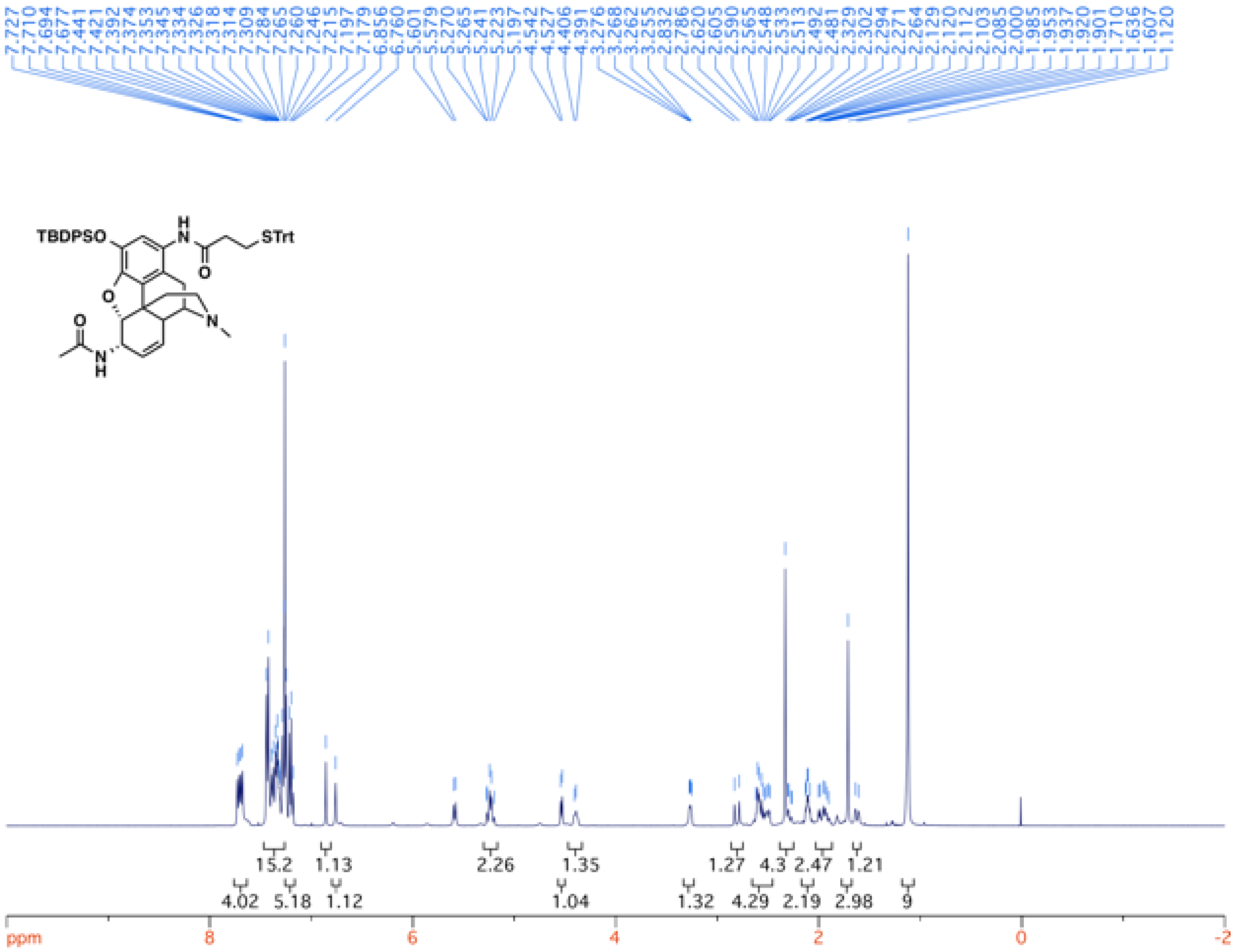

Compound **25**

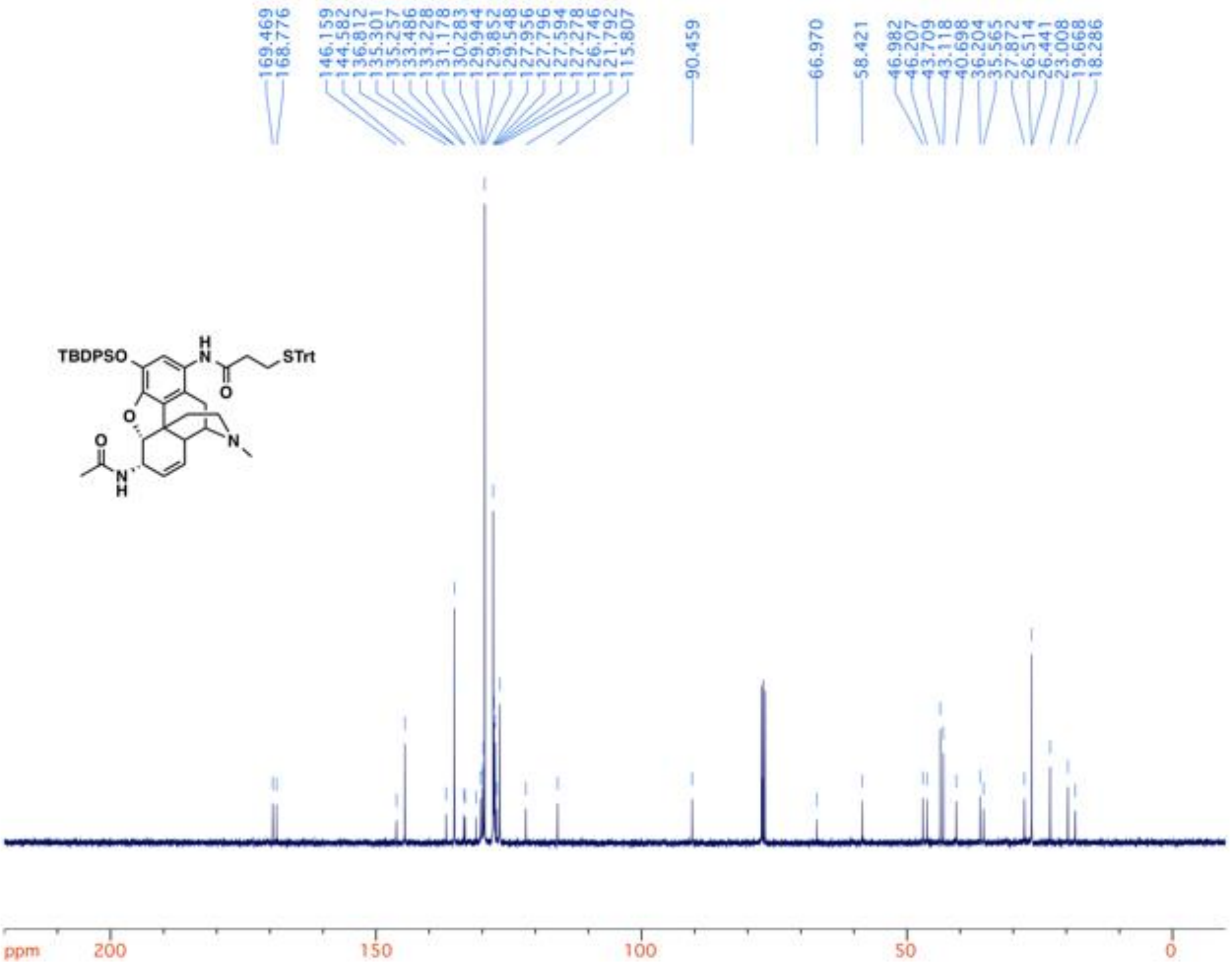
169.469
168.776
146.159
144.582
136.812
135.301
135.257
133.486
133.228
131.178
130.283
129.944
129.852
129.548
127.956
127.796
127.594
127.278
126.746
121.792
115.807
90.459
66.970
58.421
46.982
46.207
43.709
43.118
40.698
36.204
35.565
27.872
26.514
26.441
23.008
19.668
18.286
TBDPSO
STrt
ppm
200
150
100
50
0

Compound **26**

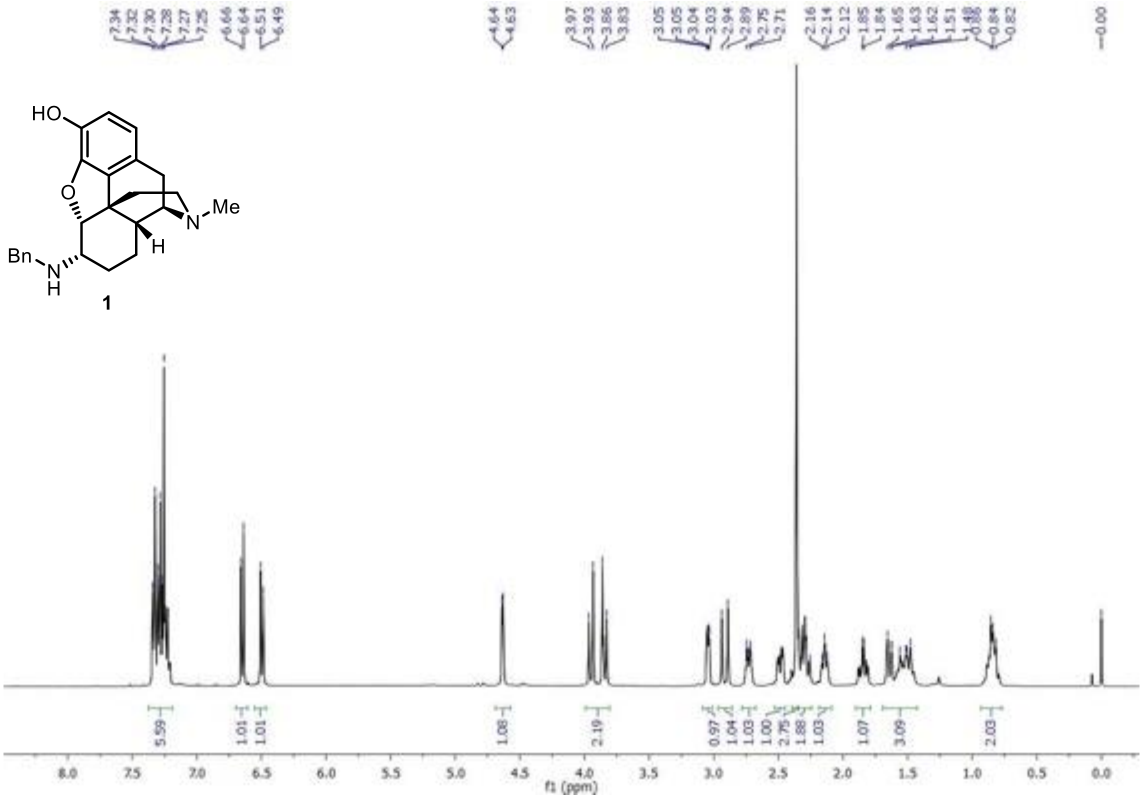

7.34 7.32 7.30 7.28 7.27 7.25 6.66 6.64 6.51 6.49
4.64 4.63
3.97 3.93 3.86 3.83
3.05 3.05 3.04 3.03 2.94 2.89 2.75 2.71
2.16 2.14 2.12 1.85 1.84 1.65 1.63 1.62 1.51 1.48 0.86 0.84 0.82
0.00
HO
O
N
Me
H
Bn
N
H
1
5.59 1.01 1.01 1.08 2.19 0.97 1.04 1.03 1.00 2.75 1.88 1.03 1.07 3.09 2.03
f1 (ppm)

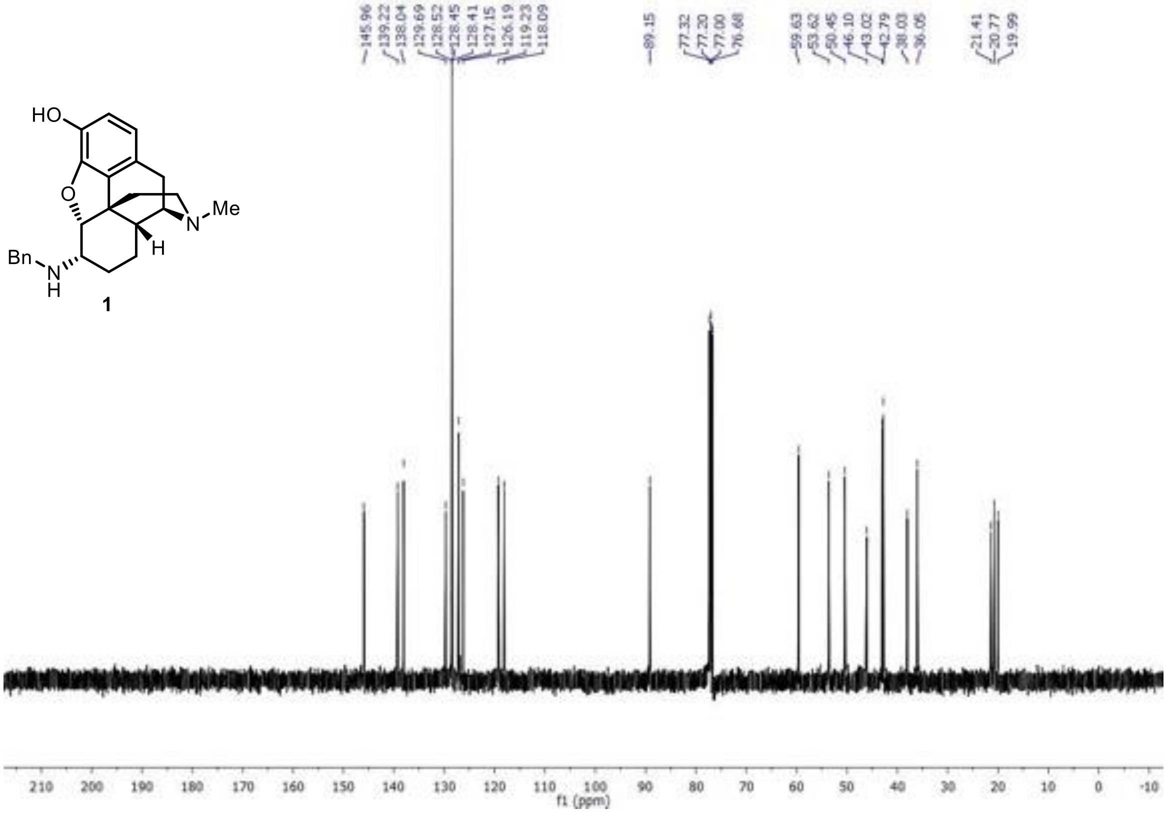

145.96 139.22 138.04 129.69 128.52 128.45 128.41 127.15 126.19 119.23 118.09
89.15
77.32 77.20 77.00 76.68
59.63 53.62 50.45 46.10 43.02 42.79 38.03 36.05
21.41 20.77 19.99
HO
O
N
Me
H
Bn
N
H
1
f1 (ppm)

Compound **29**

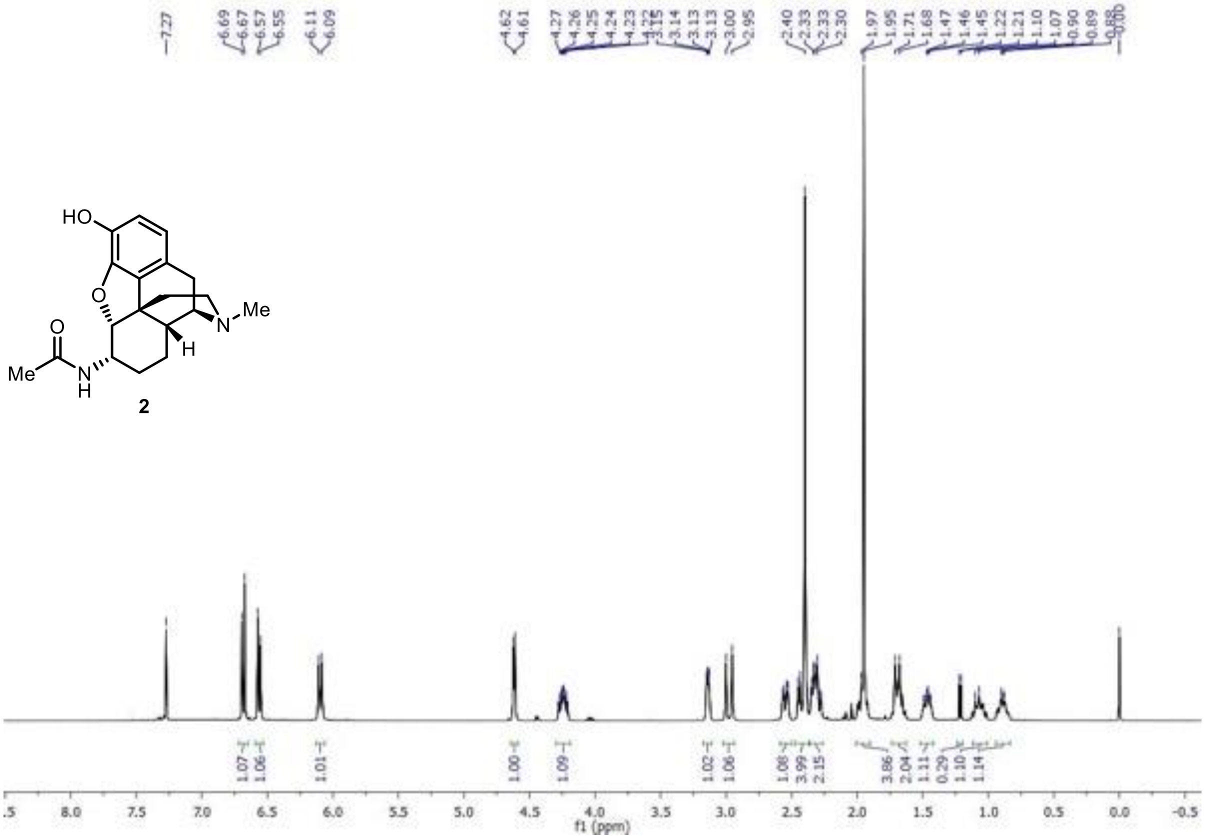

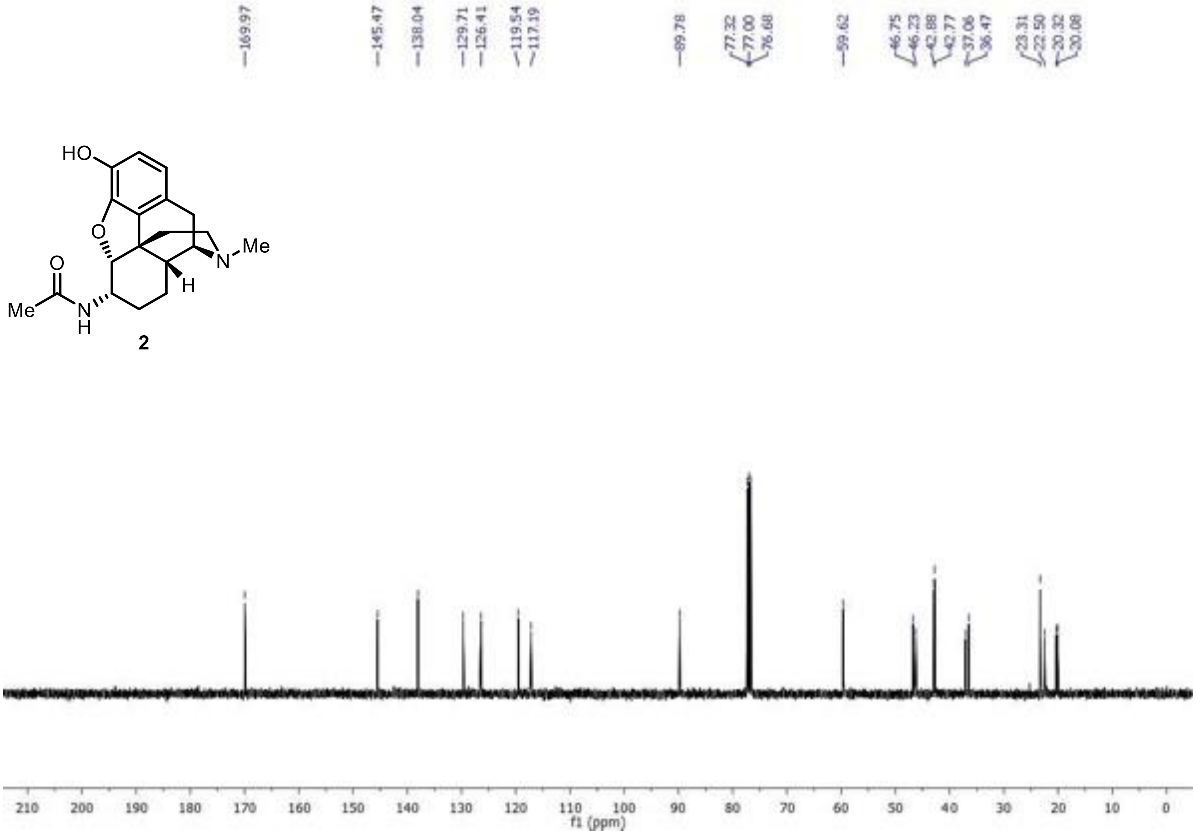

Compound **30**

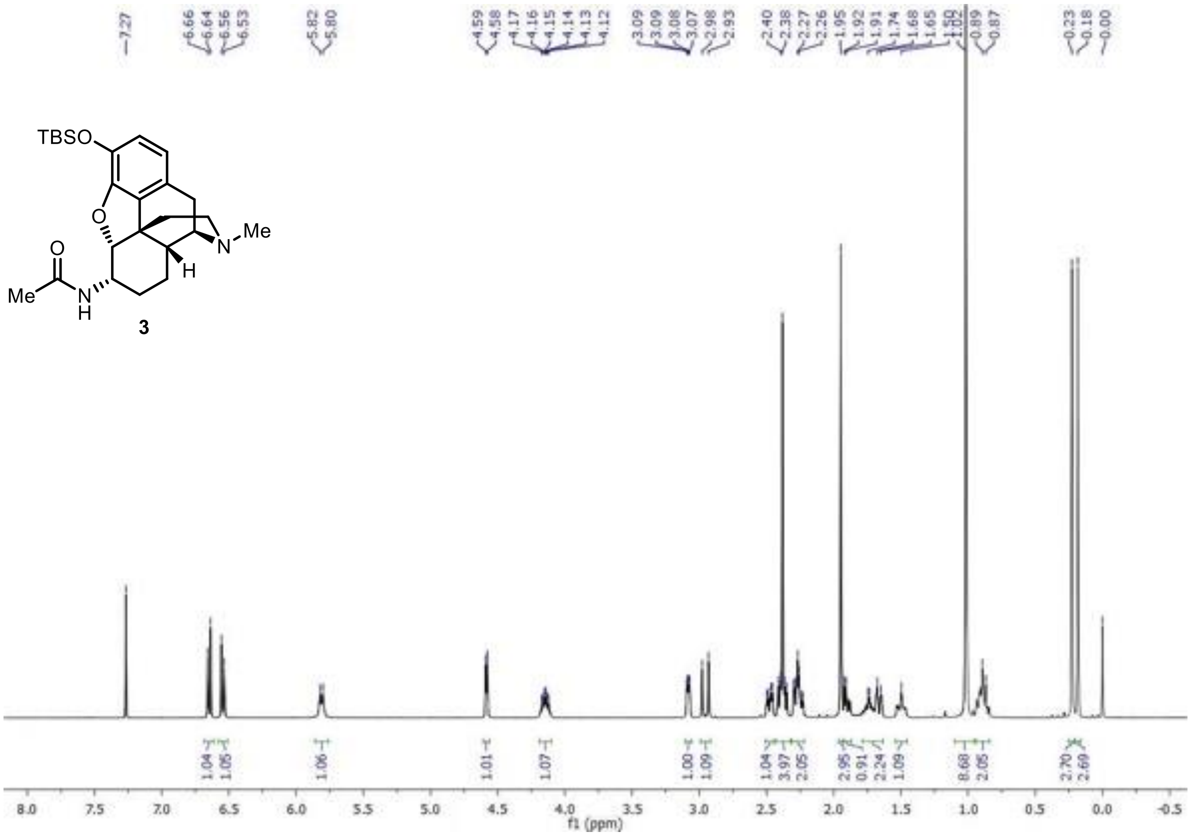

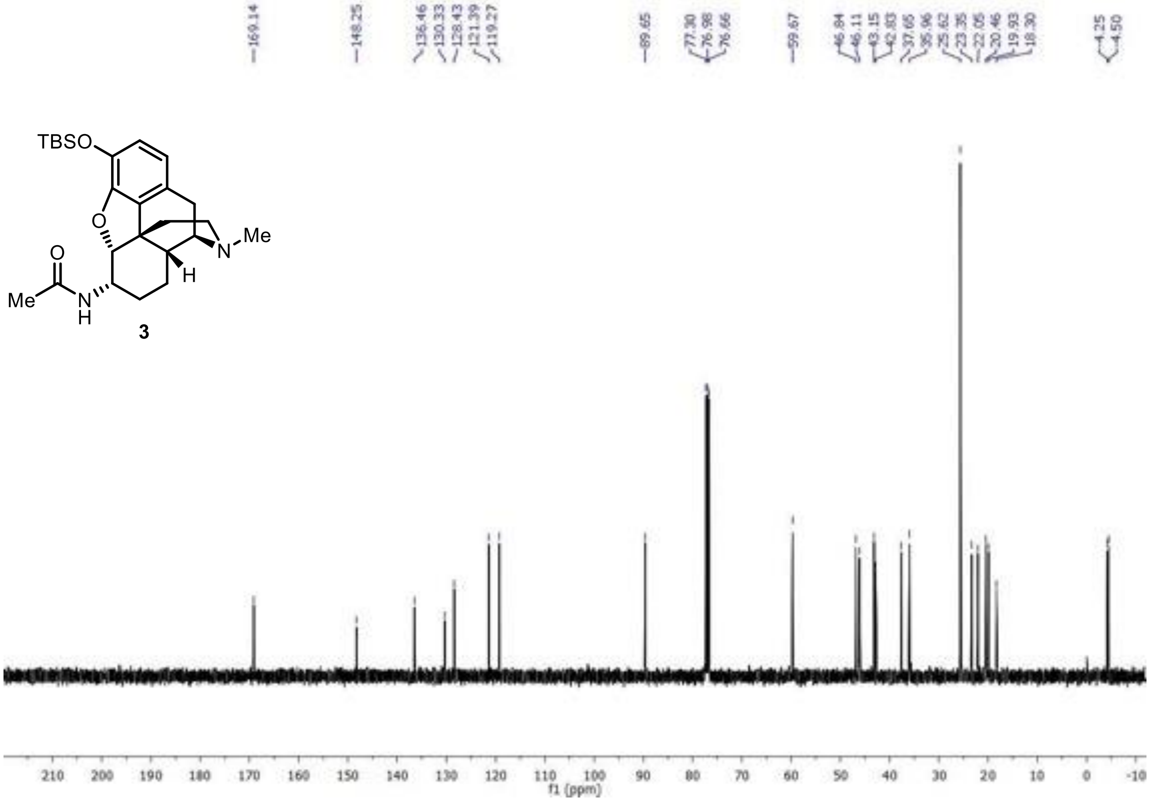

Compound **31**

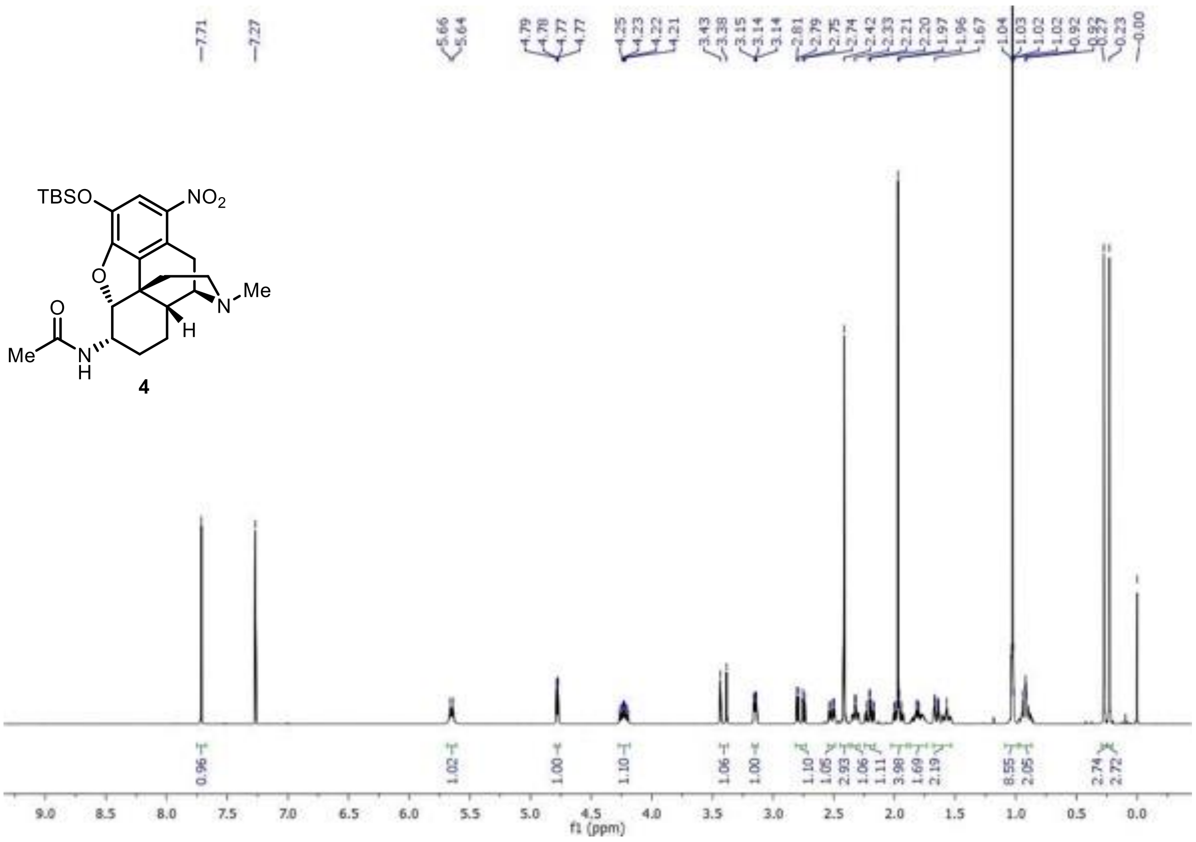

7.71
7.27
5.66
5.64
4.79
4.78
4.77
4.77
4.25
4.23
4.22
4.21
3.43
3.38
3.15
3.14
3.14
2.81
2.79
2.75
2.74
2.42
2.33
2.21
2.20
1.97
1.96
1.67
1.04
1.03
1.02
1.02
0.92
0.92
0.27
0.23
0.00
TBSO
NO_2
O
N
Me
H
O
Me
N
H
4
0.96
1.02
1.00
1.10
1.06
1.00
1.10
1.05
2.93
1.06
1.11
3.98
1.69
2.19
8.55
2.05
2.74
2.72
9.0
8.5
8.0
7.5
7.0
6.5
6.0
5.5
5.0
4.5
4.0
3.5
3.0
2.5
2.0
1.5
1.0
0.5
0.0
f1 (ppm)

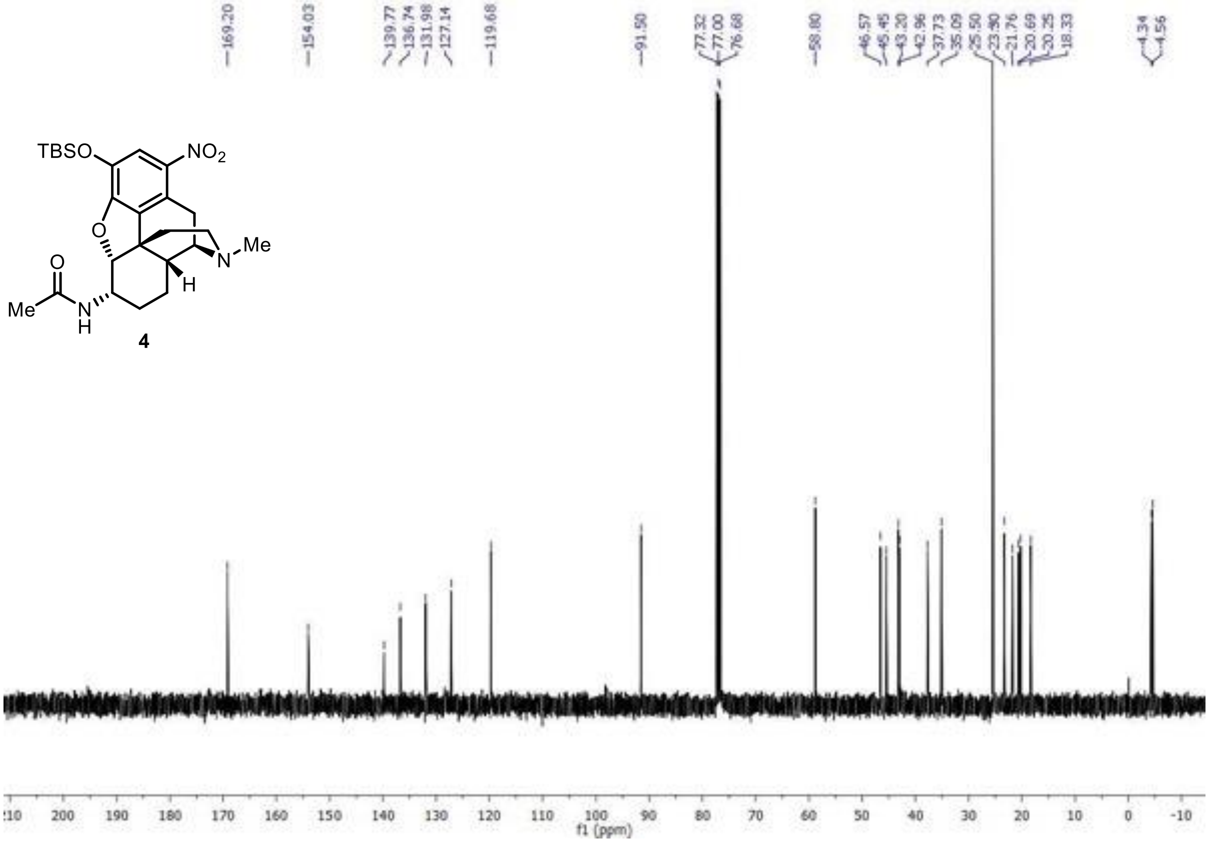

169.20
154.03
139.77
136.74
131.98
127.14
119.68
91.50
77.32
77.00
76.68
58.80
46.57
45.45
43.20
42.96
37.73
35.09
25.50
23.30
21.76
20.69
20.25
18.33
-4.34
-4.56
TBSO
NO_2
O
N
Me
H
O
Me
N
H
4
210
200
190
180
170
160
150
140
130
120
110
100
90
80
70
60
50
40
30
20
10
0
-10
f1 (ppm)

Compound **32**

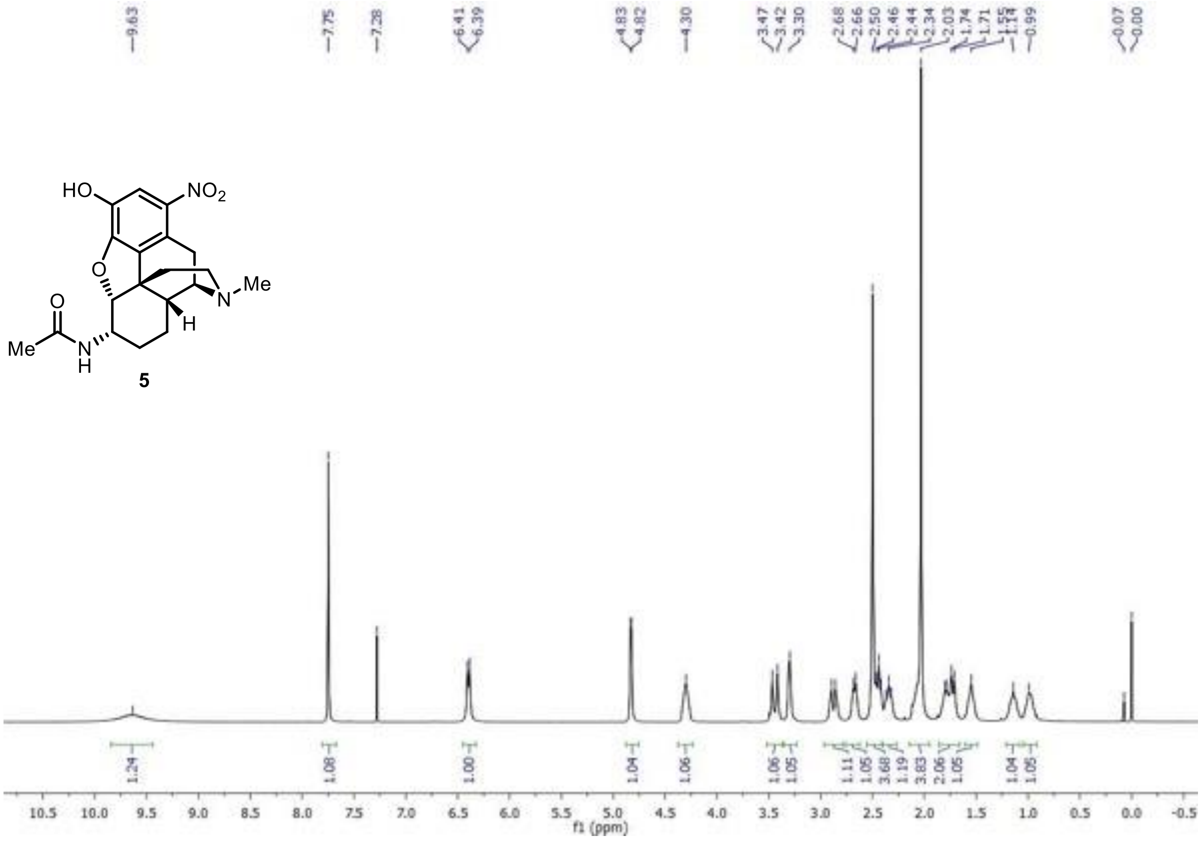
9.63
7.75
7.28
6.41
6.39
4.83
4.82
4.30
3.47
3.42
3.30
2.68
2.66
2.50
2.46
2.44
2.34
2.03
1.74
1.71
1.55
1.14
0.99
0.07
0.00
HO
NO_2
O
N
Me
H
O
Me
N
H
5
1.24
1.08
1.00
1.04
1.06
1.06
1.05
1.11
1.05
3.68
1.19
3.83
2.06
1.05
1.04
1.05
10.5 10.0 9.5 9.0 8.5 8.0 7.5 7.0 6.5 6.0 5.5 5.0 4.5 4.0 3.5 3.0 2.5 2.0 1.5 1.0 0.5 0.0 -0.5
f1 (ppm)

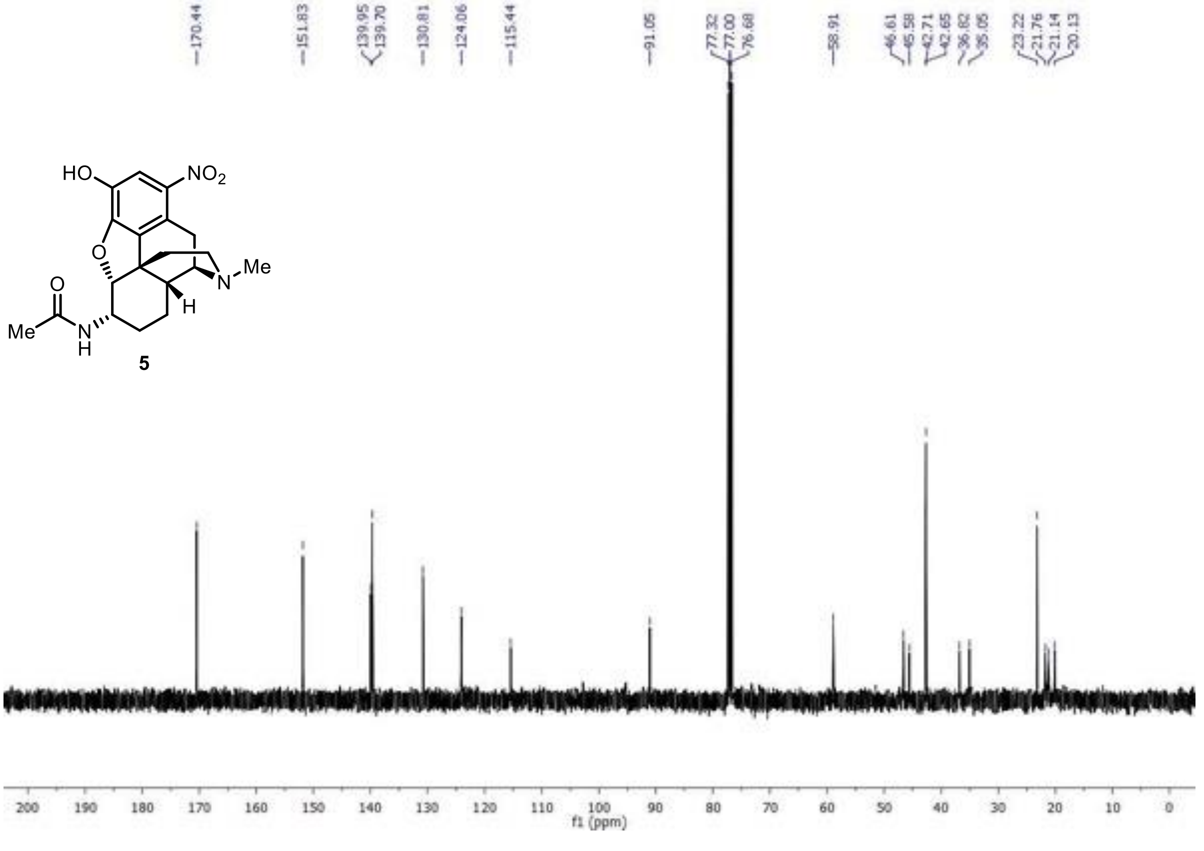
170.44
151.83
139.95
139.70
130.81
124.06
115.44
91.05
77.32
77.00
76.68
58.91
46.61
45.58
42.71
42.65
36.62
35.05
23.22
21.76
21.14
20.13
HO
NO_2
O
N
Me
H
O
Me
N
H
5
200 190 180 170 160 150 140 130 120 110 100 90 80 70 60 50 40 30 20 10 0
f1 (ppm)

Compound **34**

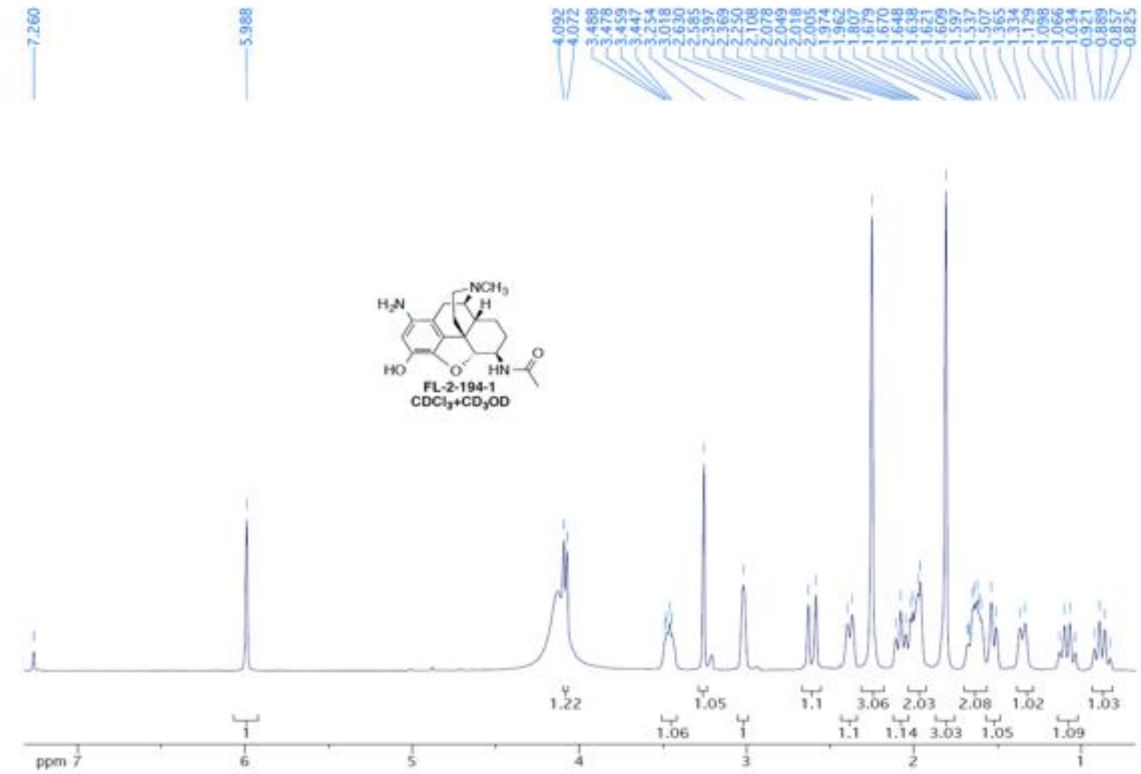
7.260
5.988
4.092
4.072
3.488
3.478
3.459
3.447
3.254
3.018
2.630
2.585
2.397
2.369
2.250
2.108
2.078
2.049
2.018
2.005
1.974
1.962
1.807
1.679
1.670
1.648
1.638
1.621
1.609
1.597
1.537
1.507
1.365
1.334
1.129
1.098
1.066
1.034
0.921
0.889
0.857
0.825
H$_2$N
NCH$_3$
H
HO
O
HN
O
FL-2-194-1
CDCl$_3$+CD$_3$OD
1.22
1.05
1.1
3.06
2.03
2.08
1.02
1.03
1
1.06
1
1.1
1.14
3.03
1.05
1.09
ppm 7
6
5
4
3
2
1

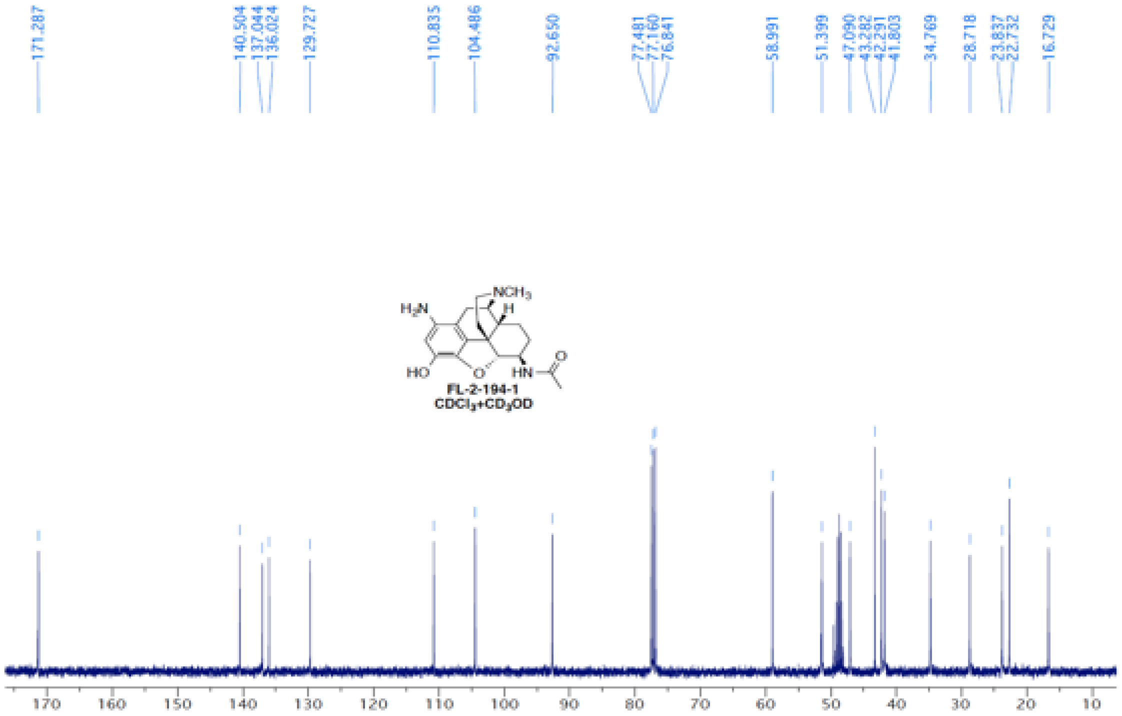
171.287
140.504
137.044
136.024
129.727
110.835
104.486
92.650
77.481
77.160
76.841
58.991
51.399
47.090
43.282
42.291
41.803
34.769
28.718
23.837
22.732
16.729
H$_2$N
NCH$_3$
H
HO
O
HN
O
FL-2-194-1
CDCl$_3$+CD$_3$OD
170
160
150
140
130
120
110
100
90
80
70
60
50
40
30
20
10

Table S1. Crystal data and structure refinement for compound **16**.

Identification code	knih109	
Empirical formula	$C_{20}H_{23}N_3O_5$	
Formula weight	385.41	
Temperature	150(2) K	
Wavelength	1.54178 Å	
Crystal system	Monoclinic	
Space group	$P2_1$	
Unit cell dimensions	a = 7.39130(10) Å	a= 90°.
	b = 9.4942(2) Å	b= 92.8700(10)°.
	c = 13.1498(3) Å	g = 90°.
Volume	921.62(3) $Å^3$	
Z	2	
Density (calculated)	1.389 Mg/m^3	
Absorption coefficient	0.836 mm^{-1}	
F(000)	408	
Crystal size	0.373 x 0.343 x 0.281 mm^3	
Theta range for data collection	3.365 to 68.217°.	
Index ranges	-8<=h<=8, -9<=k<=11, -15<=l<=15	
Reflections collected	5409	
Independent reflections	2429 [R_{int} = 0.0213]	
Completeness to theta = 67.679°	94.5 %	
Absorption correction	Semi-empirical from equivalents	
Max. and min. transmission	0.7530 and 0.7025	
Refinement method	Full-matrix least-squares on F^2	
Data / restraints / parameters	2429 / 1 / 257	
Goodness-of-fit on F^2	1.234	
Final R indices [I>2sigma(I)]	R_1 = 0.0394, wR_2 = 0.0913	
R indices (all data)	R_1 = 0.0394, wR_2 = 0.0913	
Absolute structure parameter	0.07(5)	
Extinction coefficient	0.083(4)	
Largest diff. peak and hole	0.298 and -0.283 e.$Å^{-3}$	

Table S2. Atomic coordinates (x 10^4) and equivalent isotropic displacement parameters ($Å^2$x 10^3) for compound **16**. U(eq) is defined as one third of the trace of the orthogonalized U^{ij} tensor.

	x	y	z	U(eq)
C(1)	1860(3)	5256(3)	7362(2)	25(1)
N(1)	1851(3)	3786(2)	7034(2)	29(1)
O(1A)	2391(3)	3511(2)	6188(2)	43(1)
O(1B)	1314(3)	2876(2)	7607(2)	43(1)
C(2)	1147(3)	5507(3)	8320(2)	25(1)
C(3)	1243(3)	6831(3)	8751(2)	24(1)
O(3)	686(2)	7201(2)	9684(1)	30(1)
C(3A)	-44(3)	6098(3)	10286(2)	35(1)
C(4)	2049(3)	7881(3)	8189(2)	23(1)
O(5)	2387(2)	9217(2)	8505(1)	26(1)
C(5)	3804(3)	9744(2)	7857(2)	25(1)
C(6)	5710(3)	9447(3)	8401(2)	25(1)
C(7)	6861(3)	8382(3)	7883(2)	31(1)
C(8)	6609(3)	7989(3)	6920(2)	32(1)
C(9)	4412(3)	7558(3)	5391(2)	29(1)
C(10)	3617(3)	6158(3)	5798(2)	28(1)
C(11)	2657(3)	6304(3)	6784(2)	24(1)
C(12)	2664(3)	7614(3)	7237(2)	23(1)
C(13)	3453(3)	8955(3)	6845(2)	24(1)
C(14)	5103(3)	8567(3)	6242(2)	26(1)
C(15)	2059(3)	9723(3)	6138(2)	31(1)
C(16)	1562(3)	8861(3)	5190(2)	35(1)
N(17)	3190(3)	8382(3)	4700(2)	34(1)
C(17)	2754(4)	7678(4)	3734(2)	46(1)
N(18)	5561(2)	9024(2)	9454(1)	27(1)
C(19)	4918(3)	9925(3)	10134(2)	28(1)
O(19)	4717(3)	11182(2)	9938(2)	36(1)
C(20)	4418(4)	9297(3)	11129(2)	36(1)

Table S3. Bond lengths [Å] and angles [°] for compound **16**.

C(1)-C(11)	1.400(3)	C(1)-C(2)	1.409(3)
C(1)-N(1)	1.460(3)	N(1)-O(1B)	1.226(3)
N(1)-O(1A)	1.229(3)	C(2)-C(3)	1.380(3)
C(2)-H(2A)	0.9500	C(3)-O(3)	1.359(3)
C(3)-C(4)	1.393(3)	O(3)-C(3A)	1.434(3)
C(3A)-H(3AA)	0.9800	C(3A)-H(3AB)	0.9800
C(3A)-H(3AC)	0.9800	C(4)-O(5)	1.355(3)
C(4)-C(12)	1.376(3)	O(5)-C(5)	1.471(2)
C(5)-C(13)	1.538(3)	C(5)-C(6)	1.573(3)
C(5)-H(5A)	1.0000	C(6)-N(18)	1.451(3)
C(6)-C(7)	1.506(3)	C(6)-H(6A)	1.0000
C(7)-C(8)	1.324(4)	C(7)-H(7A)	0.9500
C(8)-C(14)	1.495(3)	C(8)-H(8A)	0.9500
C(9)-N(17)	1.473(3)	C(9)-C(14)	1.540(3)
C(9)-C(10)	1.559(4)	C(9)-H(9A)	1.0000
C(10)-C(11)	1.515(3)	C(10)-H(10A)	0.9900
C(10)-H(10B)	0.9900	C(11)-C(12)	1.379(3)
C(12)-C(13)	1.503(3)	C(13)-C(14)	1.533(3)
C(13)-C(15)	1.538(3)	C(14)-H(14A)	1.0000
C(15)-C(16)	1.521(4)	C(15)-H(15A)	0.9900
C(15)-H(15B)	0.9900	C(16)-N(17)	1.466(3)
C(16)-H(16A)	0.9900	C(16)-H(16B)	0.9900
N(17)-C(17)	1.457(3)	C(17)-H(17A)	0.9800
C(17)-H(17B)	0.9800	C(17)-H(17C)	0.9800
N(18)-C(19)	1.342(3)	N(18)-H(18A)	0.8800
C(19)-O(19)	1.229(3)	C(19)-C(20)	1.501(3)
C(20)-H(20A)	0.9800	C(20)-H(20B)	0.9800
C(20)-H(20C)	0.9800		
C(11)-C(1)-C(2)	123.4(2)	C(11)-C(1)-N(1)	120.93(19)
C(2)-C(1)-N(1)	115.5(2)	O(1B)-N(1)-O(1A)	122.4(2)
O(1B)-N(1)-C(1)	119.28(19)	O(1A)-N(1)-C(1)	118.29(19)
C(3)-C(2)-C(1)	120.6(2)	C(3)-C(2)-H(2A)	119.7
C(1)-C(2)-H(2A)	119.7	O(3)-C(3)-C(2)	126.5(2)
O(3)-C(3)-C(4)	116.8(2)	C(2)-C(3)-C(4)	116.57(19)
C(3)-O(3)-C(3A)	116.62(19)	O(3)-C(3A)-H(3AA)	109.5
O(3)-C(3A)-H(3AB)	109.5	H(3AA)-C(3A)-H(3AB)	109.5
O(3)-C(3A)-H(3AC)	109.5	H(3AA)-C(3A)-H(3AC)	109.5
H(3AB)-C(3A)-H(3AC)	109.5	O(5)-C(4)-C(12)	112.8(2)
O(5)-C(4)-C(3)	125.73(19)	C(12)-C(4)-C(3)	121.4(2)
C(4)-O(5)-C(5)	105.43(16)	O(5)-C(5)-C(13)	103.97(17)
O(5)-C(5)-C(6)	108.86(17)	C(13)-C(5)-C(6)	114.48(18)
O(5)-C(5)-H(5A)	109.	C(13)-C(5)-H(5A)	109.8
C(6)-C(5)-H(5A)	109.8	N(18)-C(6)-C(7)	108.5(2)

Table S3. (continued).

N(18)-C(6)-C(5)	112.01(17)	C(7)-C(6)-C(5)	115.3(2)
N(18)-C(6)-H(6A)	106.8	C(7)-C(6)-H(6A)	106.8
C(5)-C(6)-H(6A)	106.8	C(8)-C(7)-C(6)	124.5(2)
C(8)-C(7)-H(7A)	117.8	C(6)-C(7)-H(7A)	117.8
C(7)-C(8)-C(14)	122.2(2)	C(7)-C(8)-H(8A)	118.9
C(14)-C(8)-H(8A)	118.9	N(17)-C(9)-C(14)	106.8(2)
N(17)-C(9)-C(10)	115.7(2)	C(14)-C(9)-C(10)	113.48(18)
N(17)-C(9)-H(9A)	106.8	C(14)-C(9)-H(9A)	106.8
C(10)-C(9)-H(9A)	106.8	C(11)-C(10)-C(9)	114.6(2)
C(11)-C(10)-H(10A)	108.6	C(9)-C(10)-H(10A)	108.6
C(11)-C(10)-H(10B)	108.6	C(9)-C(10)-H(10B)	108.6
H(10A)-C(10)-H(10B)	107.6	C(12)-C(11)-C(1)	113.48(19)
C(12)-C(11)-C(10)	117.4(2)	C(1)-C(11)-C(10)	128.9(2)
C(4)-C(12)-C(11)	124.4(2)	C(4)-C(12)-C(13)	107.9(2)
C(11)-C(12)-C(13)	127.49(18)	C(12)-C(13)-C(14)	107.88(19)
C(12)-C(13)-C(15)	110.46(19)	C(14)-C(13)-C(15)	109.12(18)
C(12)-C(13)-C(5)	99.64(17)	C(14)-C(13)-C(5)	117.44(19)
C(15)-C(13)-C(5)	111.77(19)	C(8)-C(14)-C(13)	111.53(18)
C(8)-C(14)-C(9)	114.5(2)	C(13)-C(14)-C(9)	106.35(18)
C(8)-C(14)-H(14A)	108.1	C(13)-C(14)-H(14A)	108.1
C(9)-C(14)-H(14A)	108.1	C(16)-C(15)-C(13)	111.6(2)
C(16)-C(15)-H(15A)	109.3	C(13)-C(15)-H(15A)	109.3
C(16)-C(15)-H(15B)	109.3	C(13)-C(15)-H(15B)	109.3
H(15A)-C(15)-H(15B)	108.0	N(17)-C(16)-C(15)	110.9(2)
N(17)-C(16)-H(16A)	109.5	C(15)-C(16)-H(16A)	109.5
N(17)-C(16)-H(16B)	109.5	C(15)-C(16)-H(16B)	109.5
H(16A)-C(16)-H(16B)	108.0	C(17)-N(17)-C(16)	112.0(2)
C(17)-N(17)-C(9)	113.4(2)	C(16)-N(17)-C(9)	112.66(18)
N(17)-C(17)-H(17A)	109.5	N(17)-C(17)-H(17B)	109.5
H(17A)-C(17)-H(17B)	109.5	N(17)-C(17)-H(17C)	109.5
H(17A)-C(17)-H(17C)	109.5	H(17B)-C(17)-H(17C)	109.5
C(19)-N(18)-C(6)	120.4(2)	C(19)-N(18)-H(18A)	119.8
C(6)-N(18)-H(18A)	119.8	O(19)-C(19)-N(18)	121.5(2)
O(19)-C(19)-C(20)	122.5(2)	N(18)-C(19)-C(20)	116.1(2)
C(19)-C(20)-H(20A)	109.5	C(19)-C(20)-H(20B)	109.5
H(20A)-C(20)-H(20B)	109.5	C(19)-C(20)-H(20C)	109.5
H(20A)-C(20)-H(20C)	109.5	H(20B)-C(20)-H(20C)	109.5

Table S4. Anisotropic displacement parameters (Å^{2}x 10^3) for compound **16**. The anisotropic displacement factor exponent takes the form: $-2p^2[h^2a^{*2}U^{11} + ... + 2 h k a^* b^* U^{12}]$

	U^{11}	U^{22}	U^{33}	U^{23}	U^{13}	U^{12}
C(1)	26(1)	20(1)	28(1)	-1(1)	3(1)	2(1)
N(1)	32(1)	22(1)	33(1)	-1(1)	6(1)	0(1)
O(1A)	61(1)	28(1)	41(1)	-11(1)	23(1)	-3(1)
O(1B)	68(1)	21(1)	43(1)	3(1)	15(1)	-6(1)
C(2)	24(1)	25(1)	27(1)	3(1)	5(1)	0(1)
C(3)	21(1)	28(1)	25(1)	-1(1)	8(1)	1(1)
O(3)	37(1)	29(1)	26(1)	-1(1)	15(1)	-2(1)
C(3A)	40(1)	35(2)	29(1)	4(1)	16(1)	-2(1)
C(4)	20(1)	24(1)	25(1)	0(1)	3(1)	2(1)
O(5)	28(1)	22(1)	28(1)	-4(1)	10(1)	-1(1)
C(5)	30(1)	19(1)	27(1)	1(1)	8(1)	-1(1)
C(6)	30(1)	21(1)	26(1)	1(1)	4(1)	-3(1)
C(7)	25(1)	36(2)	32(1)	2(1)	4(1)	3(1)
C(8)	26(1)	37(1)	32(1)	-1(1)	10(1)	5(1)
C(9)	34(1)	31(1)	24(1)	1(1)	9(1)	1(1)
C(10)	34(1)	26(1)	25(1)	-2(1)	10(1)	1(1)
C(11)	26(1)	23(1)	23(1)	0(1)	4(1)	2(1)
C(12)	22(1)	21(1)	25(1)	3(1)	4(1)	2(1)
C(13)	27(1)	20(1)	25(1)	2(1)	7(1)	1(1)
C(14)	29(1)	27(1)	24(1)	1(1)	9(1)	-2(1)
C(15)	36(1)	25(1)	31(1)	5(1)	2(1)	4(1)
C(16)	37(1)	37(2)	31(1)	5(1)	-1(1)	3(1)
N(17)	42(1)	38(1)	23(1)	2(1)	3(1)	-1(1)
C(17)	57(2)	55(2)	25(1)	-1(1)	2(1)	-5(1)
N(18)	34(1)	22(1)	25(1)	1(1)	3(1)	1(1)
C(19)	28(1)	28(1)	28(1)	-4(1)	-2(1)	-2(1)
O(19)	48(1)	21(1)	39(1)	-5(1)	2(1)	-1(1)
C(20)	42(1)	40(2)	26(1)	-2(1)	0(1)	-1(1)

Table S5. Hydrogen coordinates (x 10^4) and isotropic displacement parameters (Å^{2}x 10^3) for compound **16**.

	x	y	z	U(eq)
H(2A)	597	4759	8671	30
H(3AA)	-318	6469	10956	52
H(3AB)	844	5334	10369	52
H(3AC)	-1156	5735	9943	52
H(5A)	3647	10780	7745	30
H(6A)	6392	10357	8410	30
H(7A)	7835	7964	8273	37
H(8A)	7410	7319	6651	38
H(9A)	5484	7297	4996	35
H(10A)	2752	5770	5271	34
H(10B)	4615	5470	5902	34
H(14A)	5549	9444	5915	31
H(15A)	953	9910	6510	37
H(15B)	2564	10641	5934	37
H(16A)	834	8034	5379	42
H(16B)	815	9441	4704	42
H(17A)	2176	8350	3254	68
H(17B)	1924	6894	3843	68
H(17C)	3868	7318	3455	68
H(18A)	5896	8172	9648	32
H(20A)	4465	10028	11656	54
H(20B)	5273	8543	11324	54
H(20C)	3189	8909	11058	54

Table S6. Torsion angles [°] for compound **16**.

C(11)-C(1)-N(1)-O(1B)	172.4(2)	C(2)-C(1)-N(1)-O(1B)	-2.9(3)
C(11)-C(1)-N(1)-O(1A)	-7.5(3)	C(2)-C(1)-N(1)-O(1A)	177.2(2)
C(11)-C(1)-C(2)-C(3)	-1.4(3)	N(1)-C(1)-C(2)-C(3)	173.8(2)
C(1)-C(2)-C(3)-O(3)	-176.8(2)	C(1)-C(2)-C(3)-C(4)	0.7(3)
C(2)-C(3)-O(3)-C(3A)	1.2(3)	C(4)-C(3)-O(3)-C(3A)	-176.40(19)
O(3)-C(3)-C(4)-O(5)	2.7(3)	C(2)-C(3)-C(4)-O(5)	-175.1(2)
O(3)-C(3)-C(4)-C(12)	179.77(19)	C(2)-C(3)-C(4)-C(12)	2.0(3)
C(12)-C(4)-O(5)-C(5)	-18.1(2)	C(3)-C(4)-O(5)-C(5)	159.3(2)
C(4)-O(5)-C(5)-C(13)	30.4(2)	C(4)-O(5)-C(5)-C(6)	-92.1(2)
O(5)-C(5)-C(6)-N(18)	-12.0(3)	C(13)-C(5)-C(6)-N(18)	-127.9(2)
O(5)-C(5)-C(6)-C(7)	112.7(2)	C(13)-C(5)-C(6)-C(7)	-3.1(3)
N(18)-C(6)-C(7)-C(8)	145.1(2)	C(5)-C(6)-C(7)-C(8)	18.5(4)
C(6)-C(7)-C(8)-C(14)	-0.8(4)	N(17)-C(9)-C(10)-C(11)	-90.0(2)
C(14)-C(9)-C(10)-C(11)	34.0(3)	C(2)-C(1)-C(11)-C(12)	-0.6(3)
N(1)-C(1)-C(11)-C(12)	-175.6(2)	C(2)-C(1)-C(11)-C(10)	174.0(2)
N(1)-C(1)-C(11)-C(10)	-0.9(4)	C(9)-C(10)-C(11)-C(12)	-3.1(3)
C(9)-C(10)-C(11)-C(1)	-177.5(2)	O(5)-C(4)-C(12)-C(11)	173.1(2)
C(3)-C(4)-C(12)-C(11)	-4.3(3)	O(5)-C(4)-C(12)-C(13)	-2.4(3)
C(3)-C(4)-C(12)-C(13)	-179.87(18)	C(1)-C(11)-C(12)-C(4)	3.4(3)
C(10)-C(11)-C(12)-C(4)	-171.8(2)	C(1)-C(11)-C(12)-C(13)	178.1(2)
C(10)-C(11)-C(12)-C(13)	2.8(3)	C(4)-C(12)-C(13)-C(14)	143.53(19)
C(11)-C(12)-C(13)-C(14)	-31.8(3)	C(4)-C(12)-C(13)-C(15)	-97.3(2)
C(11)-C(12)-C(13)-C(15)	87.4(3)	C(4)-C(12)-C(13)-C(5)	20.4(2)
C(11)-C(12)-C(13)-C(5)	-154.9(2)	O(5)-C(5)-C(13)-C(12)	-30.1(2)
C(6)-C(5)-C(13)-C(12)	88.5(2)	O(5)-C(5)-C(13)-C(14)	-146.2(2)
C(6)-C(5)-C(13)-C(14)	-27.5(3)	O(5)-C(5)-C(13)-C(15)	86.6(2)
C(6)-C(5)-C(13)-C(15)	-154.74(19)	C(7)-C(8)-C(14)-C(13)	-30.3(3)
C(7)-C(8)-C(14)-C(9)	-151.1(2)	C(12)-C(13)-C(14)-C(8)	-67.6(3)
C(15)-C(13)-C(14)-C(8)	172.4(2)	C(5)-C(13)-C(14)-C(8)	43.9(3)
C(12)-C(13)-C(14)-C(9)	58.0(2)	C(15)-C(13)-C(14)-C(9)	-62.1(2)
C(5)-C(13)-C(14)-C(9)	169.42(19)	N(17)-C(9)-C(14)-C(8)	-169.98(17)
C(10)-C(9)-C(14)-C(8)	61.3(3)	N(17)-C(9)-C(14)-C(13)	66.4(2)
C(10)-C(9)-C(14)-C(13)	-62.4(2)	C(12)-C(13)-C(15)-C(16)	-63.8(2)
C(14)-C(13)-C(15)-C(16)	54.7(3)	C(5)-C(13)-C(15)-C(16)	-173.73(19)
C(13)-C(15)-C(16)-N(17)	-50.4(3)	C(15)-C(16)-N(17)-C(17)	-174.1(2)
C(15)-C(16)-N(17)-C(9)	56.8(3)	C(14)-C(9)-N(17)-C(17)	166.5(2)
C(10)-C(9)-N(17)-C(17)	-66.1(3)	C(14)-C(9)-N(17)-C(16)	-65.0(3)
C(10)-C(9)-N(17)-C(16)	62.4(3)	C(7)-C(6)-N(18)-C(19)	166.7(2)
C(5)-C(6)-N(18)-C(19)	-64.8(3)	C(6)-N(18)-C(19)-O(19)	-12.0(3)
C(6)-N(18)-C(19)-C(20)	166.4(2)		

Table S7. Hydrogen bonds for compound **16** [Å and °].

D-H...A	d(D-H)	d(H...A)	d(D...A)	<(DHA)
C(3A)-H(3AA)...O(1B)#1	0.98	2.46	3.417(3)	166.2
C(5)-H(5A)...O(1B)#2	1.00	2.63	3.504(3)	145.5
C(9)-H(9A)...O(1A)#3	1.00	2.54	3.348(3)	137.3
N(18)-H(18A)...O(19)#4	0.88	2.02	2.825(3)	150.8

Symmetry transformations used to generate equivalent atoms:
#1 -x,y+1/2,-z+2   #2 x,y+1,z   #3 -x+1,y+1/2,-z+1
#4 -x+1,y-1/2,-z+2

Structures of Naloxone and Naltrexone

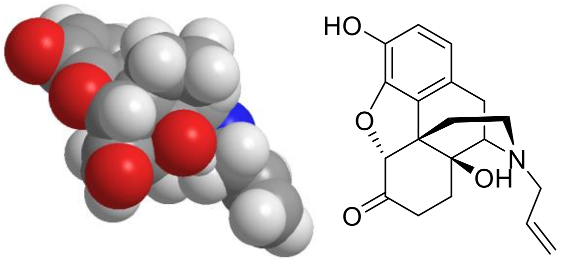

Naloxone

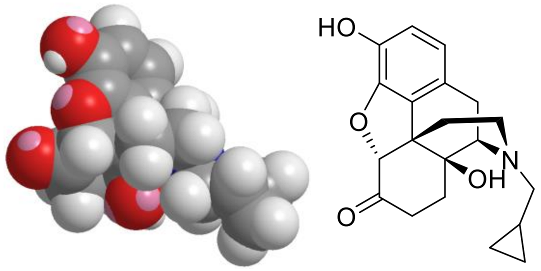

Naltrexone

3D models of the haptens and target drugs. Structures were constructed and geometry optimized using the built-in MM2 method in ChemDraw 18.1. Structures were presented in a similar orientation as in Fig. 6 (main text) to facilitate comparison.
